# Magnetism, dynamo action and the solar-stellar connection

**DOI:** 10.1007/s41116-017-0007-8

**Published:** 2017-09-26

**Authors:** Allan Sacha Brun, Matthew K. Browning

**Affiliations:** 1 Laboratoire AIM, DRF/IRFU/Département d’Astrophysique, CEA-Saclay, 91191 Gif-sur-Yvette France; 2 Department of Physics and Astronomy, University of Exeter, Stocker Road, Exeter, EX4 4QL UK

**Keywords:** Stellar magnetism, Dynamo, Sun: magnetic fields, rotation, Stars: magnetism, rotation, wind, Convection, Magnetohydrodynamics (MHD), Methods: numerical

## Abstract

The Sun and other stars are magnetic: magnetism pervades their interiors and affects their evolution in a variety of ways. In the Sun, both the fields themselves and their influence on other phenomena can be uncovered in exquisite detail, but these observations sample only a moment in a single star’s life. By turning to observations of other stars, and to theory and simulation, we may infer other aspects of the magnetism—e.g., its dependence on stellar age, mass, or rotation rate—that would be invisible from close study of the Sun alone. Here, we review observations and theory of magnetism in the Sun and other stars, with a partial focus on the “Solar-stellar connection”: i.e., ways in which studies of other stars have influenced our understanding of the Sun and vice versa. We briefly review techniques by which magnetic fields can be measured (or their presence otherwise inferred) in stars, and then highlight some key observational findings uncovered by such measurements, focusing (in many cases) on those that offer particularly direct constraints on theories of how the fields are built and maintained. We turn then to a discussion of how the fields arise in different objects: first, we summarize some essential elements of convection and dynamo theory, including a very brief discussion of mean-field theory and related concepts. Next we turn to simulations of convection and magnetism in stellar interiors, highlighting both some peculiarities of field generation in different types of stars and some unifying physical processes that likely influence dynamo action in general. We conclude with a brief summary of what we have learned, and a sampling of issues that remain uncertain or unsolved.

## Introduction

A star’s life is shaped partly by its magnetism. In its infancy and youth, magnetic fields help mediate the collapse of molecular clouds and, later, the accretion of material through a protoplanetary disk; during its main-sequence lifetime, they regulate spindown through a stellar wind; as it approaches the end stages of its evolution, they may transport angular momentum, influencing the spin rate of the interior and in turn its ultimate fate. Throughout the star’s life, its surface and interior may crackle with activity induced by the magnetic fields. Like gravity, magnetism can sculpt processes on the largest of scales; but whereas the gravitational force exerted by a star depends mainly on one parameter (its mass), its magnetism depends on a host of factors (including mass, rotation rate, stratification, and in some cases the past history of the object).

In many cases the magnetism is built by the action of a dynamo, a process that converts kinetic energy into magnetic and sustains it against resistive decay. In some others, observed fields are probably inherited from earlier stages of the star’s life, encoding (in principle) information about the interaction of various magnetohydrodynamic (MHD) instabilities acting cumulatively over aeons. In neither case do we yet have a truly comprehensive theory of the magnetism—i.e., one that would allow us to predict the magnetic field strength and geometry of a given star at a given point in its evolution. But we have many clues, derived from observation, basic theory, and numerical simulations. This paper seeks partly to review those clues.

Many of the strongest constraints on stellar magnetism have come from close study of the Sun. Our nearest star has a cyclical large-scale magnetic field, pervasive and variable smaller-scale fields, sunspots that exhibit remarkable spatial and temporal organization—and also exhibits flares, coronal mass ejections, and mass loss that are all ultimately linked to the magnetism. These features, now being probed in exquisite detail by a variety of space-based and ground-based instruments, are described in Sect. 2. Some aspects of the Sun’s magnetism can be traced (albeit indirectly) for millennia, and sunspots have been observed directly for centuries, so observational constraints abound. In this sense, the Sun is an extraordinary laboratory for plasma astrophysics—but it is a laboratory with no accessible controls. To describe how the dynamo process depends on basic parameters like stellar rotation rate or mass, we must also turn to observations (and theory) of other stars.

Observations of magnetism on other stars (described mainly in Sect. 4) also have a long history, but have lately been revolutionized by new observational instruments and techniques. Extraordinarily precise photometry has allowed fine probing of surface activity and even (through asteroseismology) provided some windows into interior dynamics as well; spectropolarimetry has begun to enable inferences of the field morphology; large surveys increasingly constrain the prevalence of magnetism and its dependence on other stellar properties.

This review focuses on Solar and stellar magnetism: how it is measured, what is found by the measurements, and how the fields are built and shaped by various physical processes. More generally, we highlight some of the ways in which close study of the Sun has informed our view of other stars, and vice versa. One of our basic premises is that while Solar observations can tell us about the Sun’s present, and to some extent its past, observations of *other* stars (and theoretical modeling) offer the best chances to understand its future. Section 2 contains an overview of observations of the Sun’s magnetism specifically, and Sect. 3 quickly summarizes some aspects of stellar evolution that are particularly influenced by (and therefore may trace) the magnetism. Sect. 4 summarizes observations of magnetic fields in other stars. In Sect. 5 we turn to a discussion of how the fields are built and maintained; that section is in essence a review of convection and dynamo theory, together with some related MHD processes. Sect. 6 describes the burgeoning role of numerical simulations in understanding stellar convection and magnetism. We close in Sect. 7 with a summary of some of the principal findings, a discussion of lingering uncertainties, and an analysis of future prospects in this area.

## The Sun: dynamics and magnetism over time

The Sun is emblematic of magnetic stars, exhibiting a large range of magnetic phenomena such as sunspots, intense flares, an extended corona and wind and a regular magnetic activity. The period of this prominent magnetic activity cycle is 22 years on average, as observed for the last 400 year and inverted for almost 600 years through the study of $^{10}$Be in ice cores (Beer et al. 1998). The overall activity level can be reconstructed over about 10,000 years through ice core techniques or by studying $^{14}$C abundances in tree rings (Miyahara et al. 2010), see review in Steinhilber et al. (2012). The 22 year magnetic cycle is formed of two consecutive sunspot cycles of about 11 years each. The activity is found to increase over 3 to 5 years and then to decline over 6 to 8 years depending on the cycle strength, stronger cycles rising faster (see, e.g., Clette and Lefèvre 2012). Being so close to our host star, we have been able to observe it continuously with increasing spatial and temporal resolutions. Many of the observed surface phenomena are directly related to the presence of intense and evolving magnetic fields.

**Fig. 1:**
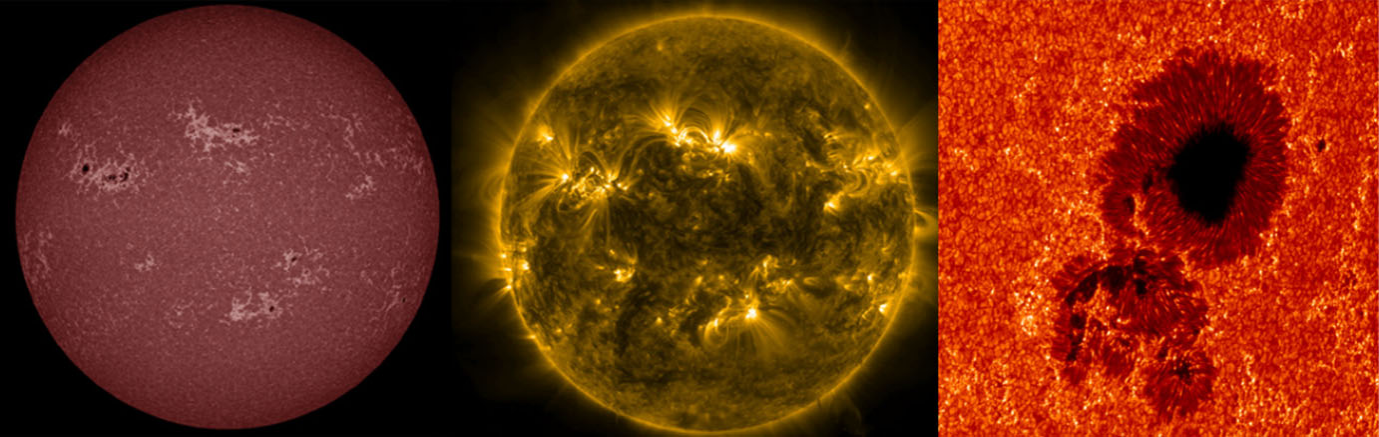
Images of the Sun from the SDO (Pesnell et al. 2012) and Hinode (Kosugi et al. 2007) satellites. *Left* continuum at 1700 Å; 10,000 K chromosphere; *Middle* Fe ix at 171 Å; 1,000,000 K corona (AIA instrument Lemen et al. (2012)); *Right* zoomed view of solar surface granulation and an active region with the SOT instrument (Tsuneta et al. 2008). Figure made using Helioviewer.org solar software interface

**Fig. 2:**
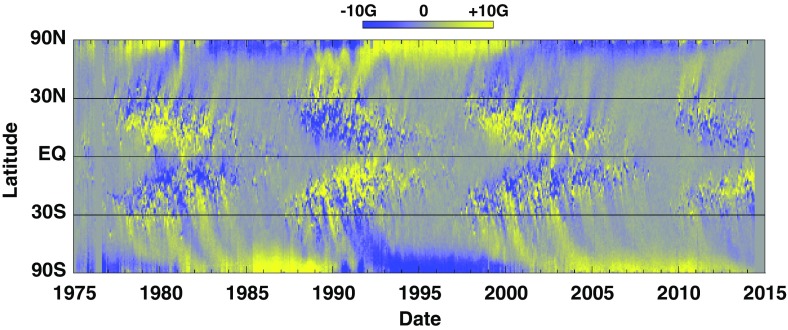
Solar butterfly diagram showing the longitudinally averaged line of sight component of the magnetic field. Notice the equatorial and polar branches of solar activity during the last 4 cycles and the reversal of polarity from one sunspot ‘11-year’ cycle to the next. Image reproduced by permission from Hathaway (2015), copyright by the author

In Fig. 1 we show typical examples of the magnetic surface of our star obtained by modern space instruments on board the SDO and Hinode satellites. In the left panel we show a global view of the Sun and of its $\sim$10,000 K chromosphere. Sunspot groups composed of dark features and bright faculae are evident and the surface seems covered by convection patterns of a typical size of 30,000 km, the so-called super-granules (see Rieutord and Rincon 2010). In the center we display a global view of the hot solar corona at about 1 MK with bright active regions associated to many closed magnetic loop-like structures, clearly located where the sunspot groups on the left image were situated. Dark coronal holes, where the magnetic field lines are open and connect to the interplanetary space, are also seen near the polar caps with few of them extending to lower latitudes. Finally on the right, we zoom in to a large sunspot group, to see its detailed structure composed of a dark umbra, a penumbra, and many bright small-scale magnetic field bundles around it. Also noticeable is the granular convection of typical size of 1000 km that paves the whole solar surface (see Living Reviews by Nordlund et al. 2009; Rieutord and Rincon 2010).

**Fig. 3:**
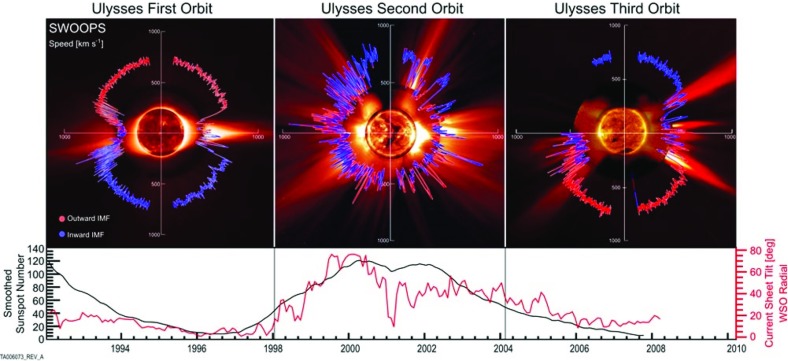
Solar wind modulation during Ulysses mission. Shown on the top panel are the fast and slow streams of the solar wind (reaching up to 800 km/s) during the activity cycle. On the bottom panel, the sun spot number (SSN) and the current sheet tilt angle during the mission are also illustrated. Image reproduced by permission from McComas et al. (2008), copyright by AGU

Such a turbulent surface convective envelope, coupled to fierce magnetism, must be partly responsible for the observed variability of solar irradiance, for large scale flows, and for the wide range of dynamical surface phenomena. But characterizing how disparate physical processes (like convection and magnetism) interact nonlinearly, and assessing how the 11 year cycle period of solar magnetic activity or the butterfly diagram of equatorward propagation of sunspots (see, e.g., Fig. 2) are established, has been a great challenge over the last century. As of today, there is a broad consensus in the community that a fluid dynamo mechanism is acting at the base and in the bulk of the convective envelope, but the details of this—including its impact on the modulation of the solar large scale flows and irradiance—are still subjects of intense research.

Observations of the surface magnetic field do not usually provide direct constraints on the possible existence of more deeply-seated magnetic fields in the radiative interior, which may nonetheless interact with the near-surface field in subtle ways. At present, the dynamics occurring in the deeper interior are probed only by helioseismology, allowing in principle the inference of links between deep-seated phenomena and surface ones (see Living Review by Gizon and Birch 2005). Such intense magnetism, and its associated complex and time varying geometry, lead to a complex coronal structure, many eruptive events (flares, coronal mass ejection [CMEs]), and shapes the solar wind and the heliosphere (see Living Reviews by Cranmer 2009; Owens and Forsyth 2013). Over an 11-year cycle various measures with satellites like Ulysses, IBEX, Voyager I & II or ground based radio observations have demonstrated that the space environment surrounding the Sun drastically changes. Close to solar maximum many fast and slow wind streams get mixed up and lead to a complex interplanetary magnetic field (see Living Review by Wood 2004, and Fig. 3).

Associated with the solar wind, there is mass and angular momentum loss (see Sect. 5.6). This has a direct consequence on the evolution of the Sun, which otherwise would only be controlled and influenced, on secular time scales, by nuclear energy generation deep inside its core and the internal structural change that this leads to. Instead the Sun also changes in time due to the action of its thermally-driven wind, going from an active rapidly rotating fully convective star during the pre-main sequence to a slowly rotating, less active old star (see Living Review by Güdel 2007). This trend linking the solar age and its magnetic and rotation state is called *gyrochronology* (Barnes 2003) and *magnetochronology* (Vidotto et al. 2014a); these concepts and what they imply for dynamo action and internal dynamics will be discussed below when we describe the various stellar phases both observationally and theoretically.

## Aspects of stellar evolution

The overall arc of a star’s life is set mostly by its mass, and the broad outlines of its evolution (on and off the main sequence) are well known to all astronomers (see, e.g., Kippenhahn et al. 2013, for a review). But on a more detailed level, the properties of stars also depend on their rotation rate and their chemical composition, all of which can be functions of time, and all of which may be affected by the presence of a stellar magnetic field. Some of these properties, most notably the rotation rate, in turn influence the magnetism. We very briefly review here some of these “second-order” effects of stellar evolution, which serve partly to motivate our lengthy consideration of stellar magnetism in the remainder of the review. Our discussion here is mostly limited to pointers to other texts and reviews where the subject is treated in greater detail. In particular, we have omitted any discussion whatsoever of chemical evolution, and refer the reader to other reviews (e.g., Michaud and Charbonneau 1991; Spite et al. 2012, and references therein) for information on that topic.

### Mass loss

Stars lose mass over time: winds and impulsive outflows are common across broad swaths of the H-R diagram, and have surely figured in the Sun’s evolution as well. For a review of mass loss in Solar-like stars, see Living Review by Wood (2004); for a broad review of winds from hotter stars, covering both observational background and theory, Kudritzki and Puls (2000). The “Sun in time” review of Güdel (2007) also contains much useful background. Various aspects of the Solar wind and Solar mass-loss specifically are reviewed in Bruno and Carbone (2013), Ofman (2010), Marsch (2006), Owens and Forsyth (2013), as well as in the textbooks by Priest (2014) and Aschwanden (2005). We have drawn on these for the brief summary below.

Today, the Sun loses mass at a rate of order a few times $10^{-14}$ solar masses per year, as measured in-situ by a series of satellites over the past few decades (beginning with the Soviet Luna-1 satellite in 1959, and culminating with the comparatively recent Ulysses, ACE, STEREO, and SOHO spacecraft). The Sun’s wind strength today varies somewhat with the solar cycle (e.g., Lazarus and McNutt 1990; McComas et al. 2008): it is actually weaker at solar maximum than at solar minimum, reflecting a dependence on the global dipole magnetic field (which is likewise weakest at solar maximum) rather than active-region-scale fields. The wind speed, density, and temperature vary with position and time, but “typical” velocities at 1 AU are hundreds of km s$^{-1}$, with proton densities of a few particles per cm$^{3}$ and temperatures of order 10$^5$ K. Mass loss via the solar wind is thus, at the present day, only of the same order as (and in fact somewhat less than) the mass lost by the Sun’s radiation ($\dot{M} \sim L_{\odot }/c^2 \approx 7 \times 10^{-14}$ $M_{\odot }$ per year).

One motivation for examining mass loss in other Solar-like stars is the well-known “faint young Sun” problem (Sagan and Mullen 1972): stellar evolution models suggest that the Sun was significantly fainter a few Gyr ago, but this would (in the absence of other changes) have implied that Earth’s radiative equilibrium temperature would be too cold for surface liquid water, in conflict with the geological and paleontological record (e.g., Kasting and Toon 1989; Sackmann and Boothroyd 2003). If the Sun’s mass loss was once much greater than it is today, for example, this would in turn imply that the Sun was more massive (and hence brighter) several Gyr ago, eliminating or reducing the faint young sun problem. (By contrast, the mass loss rate measured today, if constant over time, would imply negligible changes in the Sun’s mass, $\sim$0.05% at the ZAMS; see, e.g., Minton and Malhotra (2007).) Direct constraints on the Sun’s own mass loss over time are difficult to come by (though some constraints can be derived from, e.g., analysis of lunar rocks—see Geiss and Bochsler 1991), so we may turn again to observations of other (younger) Solar-like stars for insight.

**Fig. 4:**
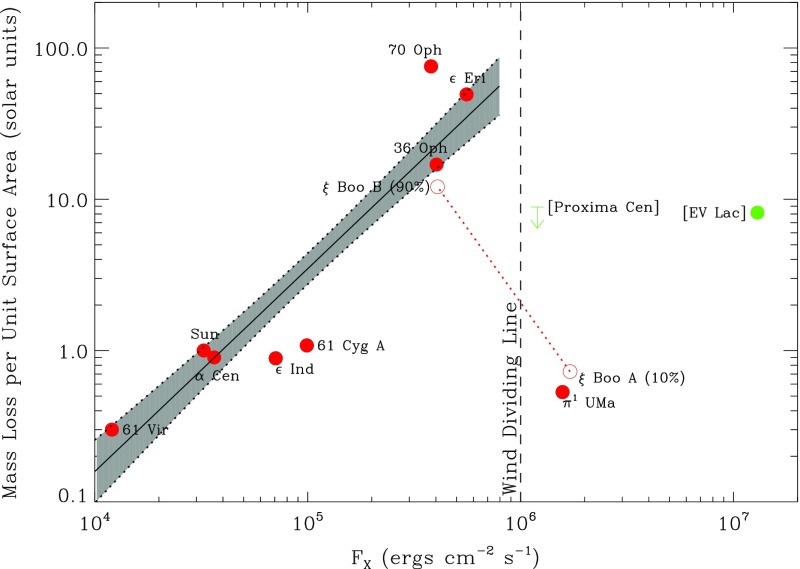
Mass loss as a function of X-ray flux, both expressed per unit stellar surface area, for main sequence stars with winds measured via the astrospheric method. For large flux the simple scaling law does not hold. Image reproduced by permission from Linsky and Wood (2014)

Unfortunately, observations of mass loss in Solar analogues are extraordinarily difficult. In principle the wind could be detected by radio (Lim and White 1996; Gaidos et al. 2000; Zarka 2007; Grießmeier et al. 2007) or X-ray emission (Wargelin and Drake 2001), but currently there are only upper limits for these measurements—excluding winds orders of magnitude stronger than that of the present-day Sun (e.g., Gaidos et al. 2000). At present, the most interesting constraints come from a somewhat indirect method, measuring essentially the interaction between the stellar wind and the surrounding interstellar medium; for a review, see Wood (2004). The wind collides with the surrounding medium, resulting in a shock and a build-up of HI gas, which can then be detected via Ly$\alpha$ absorption (see, e.g., Wood et al. 1996). Hydrodynamic calculations (e.g., Gayley et al. 1997) indicate that the amount of absorption should scale with the wind ram pressure, which in turn depends on the wind’s velocity and the mass loss rate (see Wood and Linsky 1998; Wood et al. 2002, 2005; Linsky and Wood 2014). Thus, given an assumption for the wind velocity (usually taken to be equal to the solar wind speed), measuring the astrospheric Ly$\alpha$ absorption allows inference of the mass loss rate. Though some of the steps involved in this measurement might be questioned—e.g., the assumption of solar-like wind velocities—the basic trend, namely that more Ly$\alpha$ absorption implies greater mass loss, is probably robust. Sample measurements using this technique are showcased in Fig. 4, taken from Linsky and Wood (2004). Broadly, the mass loss rate for stars on the main-sequence ($\dot{M}$) is found to correlate with stellar surface X-ray flux $F_x$,

1 $$\begin{aligned} \dot{M} \propto F_x^{1.34 \pm 0.18}, \end{aligned}$$

but this power-law relation breaks down for some of the most active stars. (The latter show $\dot{M}$ between 10 and 100 times the solar value, whereas extrapolation of the above formulae would suggest mass loss rates of up to a thousand times the Solar rate.) These measurements suggest for example that the young Sun probably had a stronger wind than it does today, but the total mass loss over its history has still been fairly small ($\sim$0.03 solar masses; see Minton and Malhotra 2007)—in particular, too small to solve (by itself) the “faint young Sun” problem.

In comparison, mass loss rates from more massive stars, and from some stars that have evolved off the main sequence, are much more amenable to direct measurement—see Kudritzki and Puls (2000) for review. Above a luminosity of $\sim$10$^{4} \, L_{\odot }$, the winds from main-sequence stars can be directly observed via spectral lines or spectral energy distributions; shocks within the wind (e.g., Lucy and White 1980; Cassinelli and Swank 1983) can also lead to further observables (in particular, ubiquitous X-ray emission from O-stars). Indeed, the signatures of such winds can be identified even in the light from distant galaxies (Steidel et al. 1996). The winds are driven mainly by radiation from the luminous central star (Castor et al. 1975). Because these winds are, both in their observed properties and their likely driving mechanisms, strikingly different from that in the Sun (and solar-like stars), we regard them as outside our “Solar-stellar connection” focus and will not discuss them further here.

We defer most discussion of the theory and modeling of winds to Sect. 5. For now, we note only that mass loss in low-mass stars is, at some level, an inevitable consequence of the presence of a hot coronae embedded in a low-pressure medium, as realized by Parker (1958). As such, we might generally expect winds whenever coronae are present; these in turn are prevalent only in certain regions of the H-R diagram (see, e.g., Linsky and Haisch 1979; Rosner et al. 1995). But many other processes—including acceleration of the wind by phenomena associated with the magnetism (see, e.g., Cranmer et al. 2007; Cranmer 2009; Réville et al. 2015b)—likely play roles in determining the wind properties.

Finally, we note that in the Sun, the overall rate of (non-radiative) mass loss is dominated by the solar wind (e.g., Howard et al. 1985); but in much more active stars—e.g., on the pre-main-sequence—it is possible that transient mass loss events, associated with flares/coronal mass ejections, could play a more significant role. Various aspects of this topic are explored in, e.g., Aarnio et al. (2012), Drake et al. (2013), Osten and Wolk (2015). In particular, Osten and Wolk (2015) argue that some active low-mass stars may lose up to $\sim$10$^{-11} \, M_{\odot }\, {\mathrm{yr}}^{-1}$ by transient events, hundreds of times the (current) solar wind rate.

### Rotational evolution

Stars spin down over time: as they lose mass, they must also lose angular momentum. The angular momentum of the departing matter is set by its angular velocity and density as a function of position; the velocity in turn must transition from co-rotation with the stellar surface out to its asymptotic value at greater distances. This in turn is influenced by the magnetic field strength and geometry: crudely, we may imagine the plasma as co-rotating with the stellar surface out to some distance $r_A$, beyond which the magnetic field is no longer strong enough to enforce this, so that the “lever arm” for angular momentum loss is $r_A$, and the angular momentum loss rate is proportional to $\dot{M} r_A$. It is important to note, though, that even in the simplest models (e.g., Parker 1958) the plasma does not actually rotate uniformly out to $r_A$, and transition to zero angular velocity outside of that; rather, the transition is smooth, but can yield a total rate of angular momentum loss that is equivalent to that of the simpler (co-rotating) model. Various theoretical aspects of angular momentum loss are discussed in Sect. 5; here, we briefly note some observational data on stellar spindown that constrain this work, and provide crucial clues regarding the interaction between rotation and magnetism.

Measurements of the spin rates of stars have a long history. Many early measurements relied on the rotational (Doppler) broadening of spectral lines (see, e.g., Shajn and Struve 1929), and variants of this technique are still in wide use today. Many others have drawn on analysis of periodic variability in a photometric lightcurve, in turn taken to arise from the rotational modulation of dark spots on the stellar surface—and these, in particular, have experienced a great renaissance in the last few years, spurred on by the increasing number of stars for which high-precision lightcurves (e.g., from the *Kepler* and COROT spacecraft) are now available. (These measurements are discussed in more detail in Sect. 4.) Substantial reviews can be found in, e.g., (Herbst et al. 2007; Irwin and Bouvier 2009; Bouvier et al. 2014); we here summarize only a few points regarding the dependence of rotation rate on age and mass.

Young solar-like stars (i.e., stars in clusters with ages of only a few Myr) have a range of spin periods *P*, typically ranging from less than a day to $\sim$10 days. These spin periods do not appear to evolve much in the early-pre-main-sequence phase (i.e., within the first $\sim$5 Myr), but as the PMS accretion phase ends and the evolving stars contract towards the main sequence, the lower envelope of the period distribution moves to even shorter periods (faster rotation), while the slowest rotators continue to have $P \sim 10$ days. On average, stars with disks appear to be somewhat slower rotators than diskless stars, with some authors interpreting this as evidence of “disk-locking” (Koenigl 1991; Rebull et al. 2004); but there is substantial overlap of spin periods between the two populations, and theoretical interpretation remains controversial.

Once stars are on the main sequence, they spin down over time in a mass-dependent fashion. Stars like the Sun quickly (i.e., within less than a Gyr) begin to follow the classic Skumanich relation (Skumanich 1972), with the stellar angular velocity decreasing with time, ${\varOmega }(t) \propto t^{-1/2}$. But the time it takes stars to “latch on” to the Skumanich relationship is clearly a function of mass: at 1 Gyr, for example, solar-type stars have spun down but lower-mass stars still exhibit substantial scatter in spin rates (e.g., Agüeros et al. 2011). At still later ages, a non-trivial fraction of low-mass M-dwarfs in the field (i.e., having ages presumed to be several Gyr) still rotate rapidly (see, e.g., Barnes 2003; Delfosse et al. 1998; Mohanty and Basri 2003; Browning et al. 2010; Irwin et al. 2011; Reiners et al. 2012; McQuillan et al. 2014; West et al. 2015; Newton et al. 2015), suggesting that the spindown time for these stars can be very long indeed. The lowest-mass stars in these samples also appear to spin down ultimately to longer periods than more-massive stars (Irwin et al. 2011; Newton et al. 2015).

**Fig. 5:**
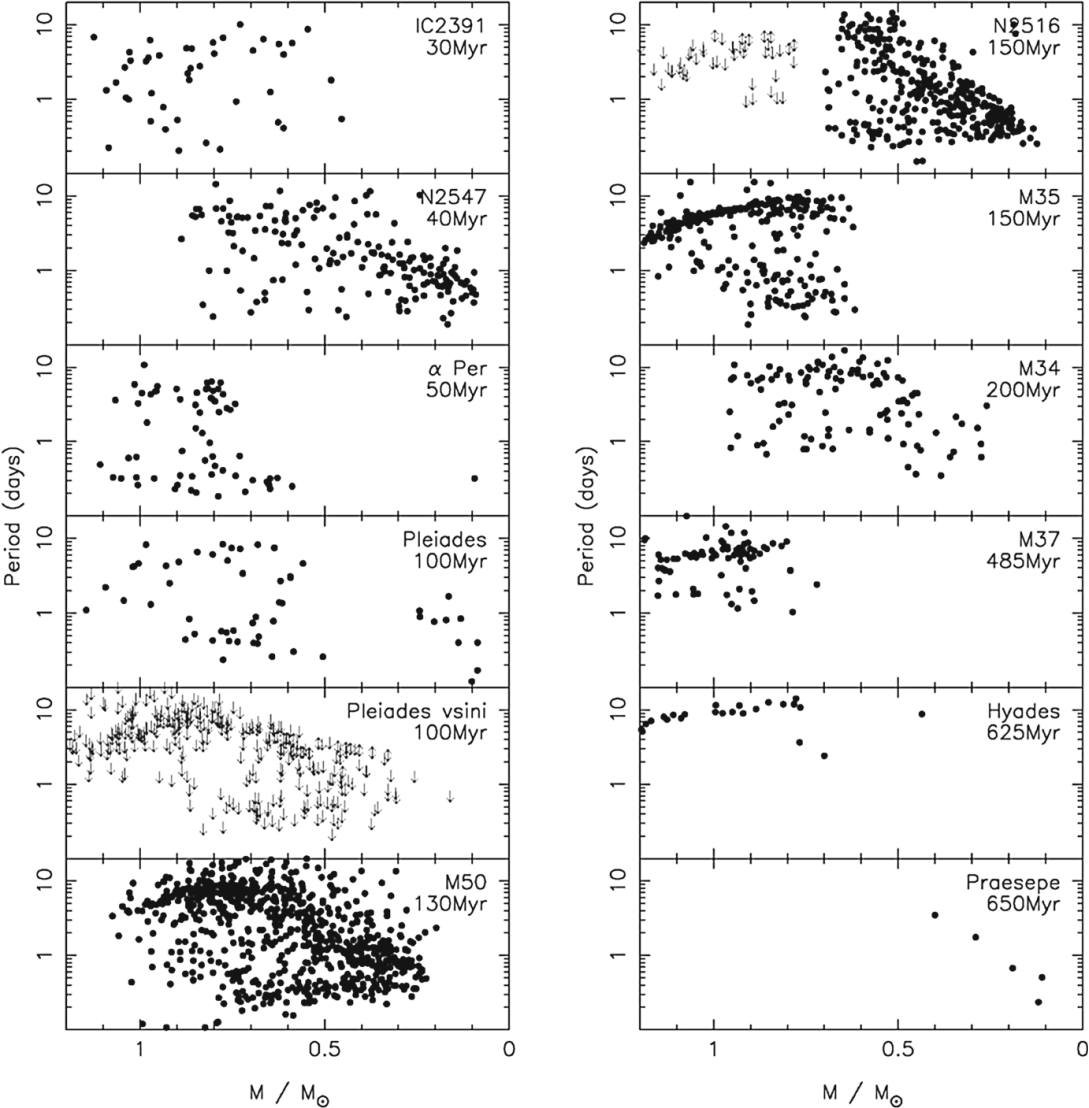
Open cluster rotational evolution. Shown are rotation periods as a function of stellar mass, for stars with masses less than $1.2 \, M_{\odot }$ in young clusters with ages ranging from 1 Myr to 0.6 Gyr. Image reproduced by permission from Irwin and Bouvier (2009)

We display some examples of these trends in Fig. 5, taken from Irwin and Bouvier (2009), which shows rotation periods for thousands of stars in young clusters of varying ages. The overall trends noted above—namely, a spread in rotation periods at all masses in the youngest clusters, and a mass-dependent convergence to a more narrow spread at later times—are clearly visible. By the age of the Hyades (bottom right panel), all stars with masses more than about $0.7 \, M_{\odot }$ have converged to spin periods of around 10 days, whereas the lowest-mass objects still have a range of periods (including some rotating much more rapidly).

At some masses, the spread in rotation periods at a given age is thought to be very narrow, so that measurement of the rotation rate gives an estimate of the age—the idea of *gyrochronology* (Barnes et al. 2001; Barnes 2003, 2007; Mamajek and Hillenbrand 2008; Barnes and Kim 2010; Epstein and Pinsonneault 2014). It should be clear from the discussion above that the age range for which this method ought to be reliable is a function of mass, and requires some calibration (i.e., a sample of stars of known age and rotation period) at each mass. For a recent example, see Meibom et al. (2015), who have extended and calibrated the gyrochronology relations of Barnes (2007) using observations of the 2.5 Gyr-old cluster NGC 6819; they conclude that age estimates with a precision $\sim$10% are possible for cool stars at this age. But see also Davies et al. (2015) and van Saders et al. (2016), who show discrepancies between the gyrochronology relations and asteroseismically-determined ages for old field stars.

For a summary of theoretical interpretations of these findings, see discussion in Sect. 5 and the reviews noted above (e.g., Bouvier et al. 2014; Brun et al. 2015b).

## Diversity of stellar dynamics and magnetism

In the Sun, observational constraints on the magnetism abound. As reviewed in Sect. 2, observations of sunspots, chromospheric and coronal activity, and long-term Earth-based proxies all help constrain the current properties and past behavior of the cyclical magnetism. In this section, we turn to observations of magnetism in other stars. Such observations are of course coarser—lacking the spatial resolution and sensitivity available for the Sun, we must often turn to various proxies of the magnetism—but nonetheless provide powerful constraints on the nature of magnetism as a function of stellar mass, rotation rate, and other properties. Thus, albeit indirectly, they provide a window into the Sun’s past and future magnetic field as well. We begin here by outlining some of the main observational techniques in use today (Sect. 4.1), before discussing key results from pre-main-sequence stars (Sect. 4.2), solar-like stars (Sect. 4.3), lower-mass stars (Sect. 4.4), and more massive stars (Sect. 4.5) (where in both cases the implicit mass comparison is to the Sun).

### Main observational techniques

In the case of the Sun, signatures of stellar activity are occasionally hard to miss: the largest active regions, for example, are visible to the naked eye. With other stars we are not so lucky. Below, we highlight four main techniques that have been widely used, describe briefly how they work, and point to more detailed reviews where appropriate: *photometric variability*, *proxies* (e.g., chromospheric and coronal heating), *Zeeman signatures and spectropolarimetry*, *seismic tracers*, and *direct imaging* (via interferometry).

**Fig. 6:**
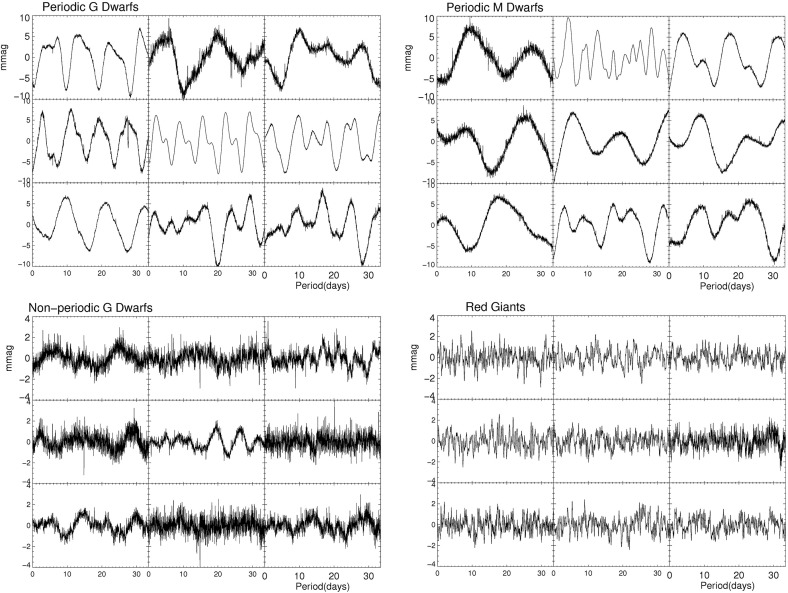
A sampling of stellar photometric variability from *Kepler*, showing representative periodic G dwarfs (*top left panels*), periodic M dwarfs (*top right*), non-periodic G dwarfs (*bottom left*), and red giants (*bottom right*). Image reproduced by permission from Basri et al. (2011), copyright by AAS

**Fig. 7:**
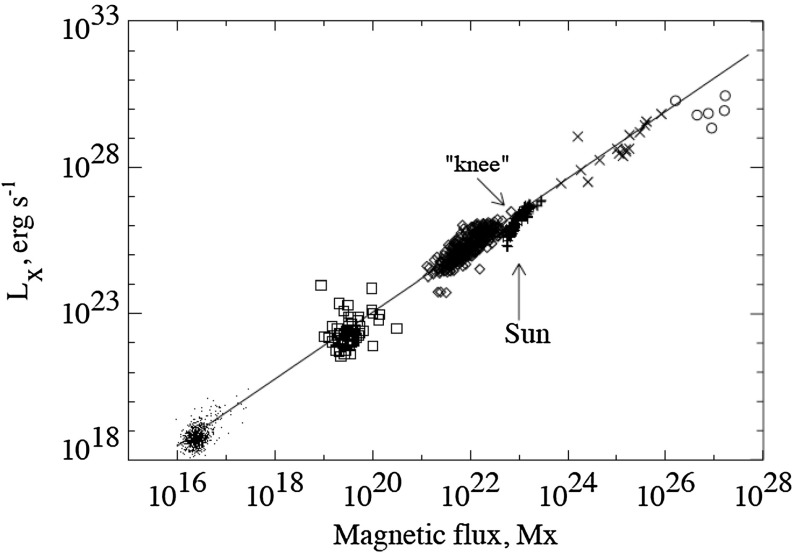
The relationship between total unsigned magnetic flux measurements (x-axis) and X-ray luminosity on different regions of the Sun. Shown are quiet Sun, X-ray bright points, active regions, and the integrated disk. Image reproduced by permission from Pevtsov et al. (2003), copyright by AAS

**Fig. 8:**
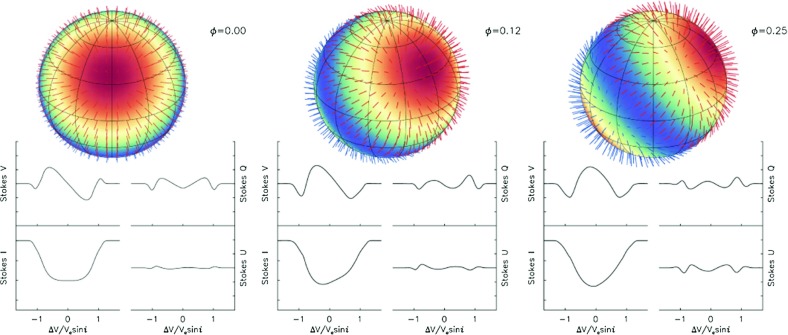
Variation of the four Stokes parameter profiles of a magnetically sensitive spectral line owing to an oblique dipolar magnetic field (sampled here at three times in the phase curve). Image adapted from Kochukhov (2016)

**Fig. 9:**
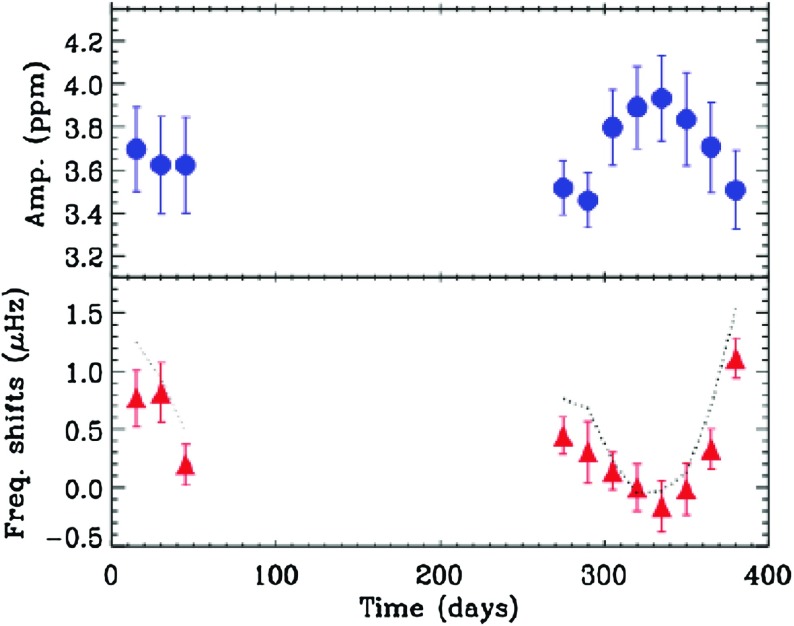
Seismic proxy for magnetic activity on HD49933. A frequency shift of the acoustic modes due to a change of magnetic activity level is observed. Image adapted from García et al. (2010)

**Fig. 10:**
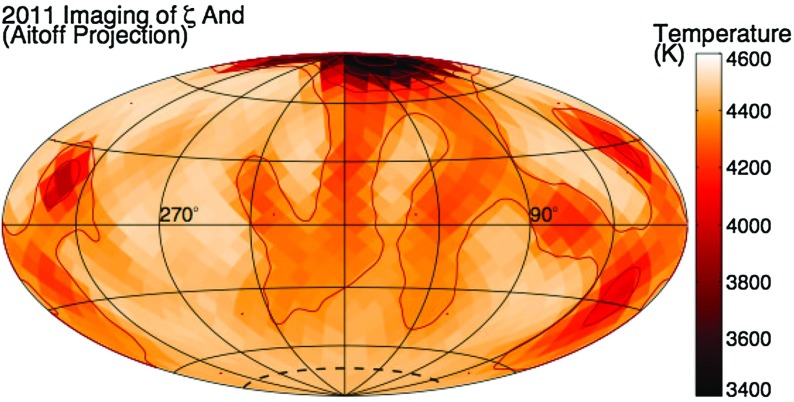
Interferometric map of surface temperature distribution on the star $\zeta$ Andromedae with CHARA. The *dark regions* are presumed to be associated with magnetic spots. Image reproduced by permission from Roettenbacher et al. (2016), copyright by Macmillan

- **Photometric variability**
  Measurements of the photometric variability of distant stars have a long history, dating back to at least the 17th century (see, e.g., review in Strassmeier 2009). Although the link to surface magnetism was not established until much later Hale (1908), we now understand that in many cases the periodic brightening and dimming of other stars can provide a wealth of information regarding the distribution of dark spots on the surface, and that often these are caused by surface magnetic fields (which are locally strong enough to affect heat transport in the plasma). The effect of activity on brightness can sometimes be counterintuitive: the Sun is brightest at solar maximum, because the darkness of the spots is more than offset by an increase in other regions (e.g., faculae and small-scale fields) that tend to appear brighter (e.g., Fröhlich 2012). The balance of these effects surely changes as a function of stellar activity (and other parameters), but a generic expectation is that at some point the spot signal probably becomes dominant (see, e.g., Lockwood et al. 2007; Hall et al. 2009; Shapiro et al. 2014).
  With the advent of space-based photometry, notably including the *Kepler* (and now K2) and COROT missions, it has lately become possible to monitor stellar variability with extraordinary precision (e.g., down to a few parts per million in some stars with *Kepler*; see Caldwell et al. 2010; Borucki 2016). Indeed, stellar activity at this level complicates the search for transiting Earth-sized planets—the photometric signature of a large active region, for example, is frequently as large as that of a planet (or larger), though the two signals can often be distinguished by their different temporal behavior; at a smaller level, granulation, faculae, and pulsations can all contribute to variability as well (see, e.g., Lanza et al. 2009; McQuillan et al. 2012). The result has been a tremendous increase in the quality and number of variability studies. A representative example is displayed in Fig. 6, taken from Basri et al. (2011), which shows both periodic and irregular variability in stars observed with *Kepler*. The periodic signatures of spots are clearly evident in such data, and provide estimates of the rotation period (e.g., Walkowicz and Basri 2013; McQuillan et al. 2014); moreover, by examining the evolution of such features with time, some aspects of the spot distribution and surface differential rotation can be inferred (e.g., Reinhold and Reiners 2013; Lanza et al. 2014; Davenport et al. 2015; Reinhold and Gizon 2015), though with considerable uncertainties (Aigrain et al. 2015). Empirically, microvariability in the light curve may also be used to estimate the stellar surface gravity (Bastien et al. 2013, 2016), though the properties of this “flicker” are not yet thoroughly understood theoretically.
- **Proxies: atmospheric heating** Although the exact mechanisms by which the solar upper atmosphere is heated remain controversial (see, e.g., discussion in Parnell and De Moortel (2012), the existence of a link between heating and magnetism is not in general dispute. Indeed, some authors *define* a stellar chromosphere/corona by the presence of non-radiative heating, which in many cases can probably only be produced in the required quantities by magnetic processes (acoustic emission being generally too small); see Linsky and Haisch (1979), and the reviews of Hall (2008), Güdel (2002, 2004). On a more detailed level, observations of the Sun suggest a clear relation (in this regime at least) between magnetic flux and coronal emission; this correlation is sampled in Fig. 7, taken from Pevtsov et al. (2003). Hence in other stars, we may turn to the presence and magnitude of such heating as a proxy for the presence of magnetism.
  These measurements, too, have a long history, as reviewed in detail by Hall (2008). The famous Mt Wilson survey of (chromospheric) Ca ii H and K emission, which operated from 1966 to 2003, provides the most comprehensive and long-running view (see summaries in, e.g., Duncan et al. 1991; Baliunas et al. 1995). Many seminal results from that survey feature in our discussion throughout this review, and many later authors have also turned to the Ca lines as measures of activity (e.g., Wright et al. 2004). The H$\alpha$ line is also commonly employed as a diagnotic of heating (e.g., Robertson et al. 2013), and in many cases correlates with Ca emission, though the link between these different tracers and the magnetism can be complex (see, e.g., Cram and Mullan 1979; Walkowicz et al. 2004; Walkowicz and Hawley 2009).
- **Zeeman signatures and spectropolarimetry** In some cases the presence of magnetism can be inferred more directly. In general, magnetic fields can affect both the spacing of energy levels (the Zeeman effect, and at higher energies the Paschen–Back effect) and the propagation of radiation. Zeeman broadening of unpolarized spectral lines can be detected in some stars (e.g., Valenti and Johns-Krull 2004), while in others (namely low-mass M-dwarfs) the magnetism gives rise to changes in the FeH molecular band (Valenti et al. 2001; Reiners and Basri 2007). These overall broadenings are sensitive to the field energy (the energy levels do not know which direction you are observing them from), and so pick up contributions from magnetism on a broad range of scales.
  The magnetism can also induce polarization of spectral lines, and by measuring these lines one can infer both the strength and some features of the geometry of the field. In particular, by constructing a time series of such spectropolarimetric measurements of a rotating star, one can infer some aspects of the surface magnetic field distribution. An extensive review can be found in Donati and Landstreet (2009); we note also (Donati et al. 1997, 2006b), and reviews in Piskunov and Kochukhov (2002) and Berdyugina (2005) as providing relevant background. A pictorial example of this is displayed in Fig. 8: as a gross generalization, the method relies on the fact that (through the Doppler shift induced by rotation), features on different regions of the star are mapped to different regions in wavelength space. Generally speaking, the Zeeman-induced polarization signal is likely to be sensitive only to fairly large-scale fields; contributions from the smallest-scale (tangled) fields tend to cancel out. Further, the field “map” reconstructed from the measurements is not apt to be unique; other (more complex) field distributions may be possible. Nonetheless, spectropolarimetric techniques—and specifically Zeeman Doppler Imaging—are virtually the only source of information about field *geometries* on stars other than the Sun. In our discussions below regarding field properties across the H-R diagram, we draw repeatedly on these measurements.
- **Seismic tracers** With the arrival of *Kepler* and COROT, the extraordinary promise of *asteroseismology* has at last begun to be realized. The surfaces of stars crackle with the signatures of acoustic and gravity waves that propagate within the interior; these modes are sensitive to various properties of the regions where they propagate, and so by observing enough of them one can constrain, for example, the internal density and rotation rate. Comparing the astereoseismic signal to other measurements obtained at the surface allows further constraints. We will defer discussion of how these constraints are derived to other very recent reviews: see, e.g., Brun et al. (2015b) for a discussion in the solar-stellar context, Chaplin and Miglio (2013) for a more focused review, the textbook by Aerts et al. (2010), and earlier review by Brown and Gilliland (1994) for background. Here, we simply note that these asteroseismic signals exist, and allow (in some cases) measurements of stellar interior rotation rates (e.g., Beck et al. 2012; Deheuvels et al. 2012), indirect probes of interior magnetic field strengths (Stello et al. 2016), and constraints on stellar radii and densities (e.g., Metcalfe et al. 2010b; Huber et al. 2013). An example of a particular seismic proxy for magnetic activity in the star HD49933, taken from (García et al. 2010), is shown in Fig. 9: here, modulations in these seismic signatures are taken to reveal an activity cycle akin to that of the Sun.
- **Interferometric imaging** Finally, it has very recently become possible to resolve the disks of a small number of stars using interferometry. As of this writing, few results have emerged, mostly involving direct measurements of the radii of stars (e.g., Huber et al. 2012; Boyajian et al. 2015; Johnson et al. 2014), but the technique holds extraordinary promise. One illustrative example is shown in Fig. 10, taken from Roettenbacher et al. (2016): here, the actual surface of the star is resolved (i.e., the resolution element is considerably smaller than the apparent radius of the star), allowing a coarse image of brightness distributions. Roettenbacher et al infer the presence of dark regions, presumed to be associated with magnetism, both at the poles and nearer the equator.

### Pre main sequence stars

**Fig. 11:**
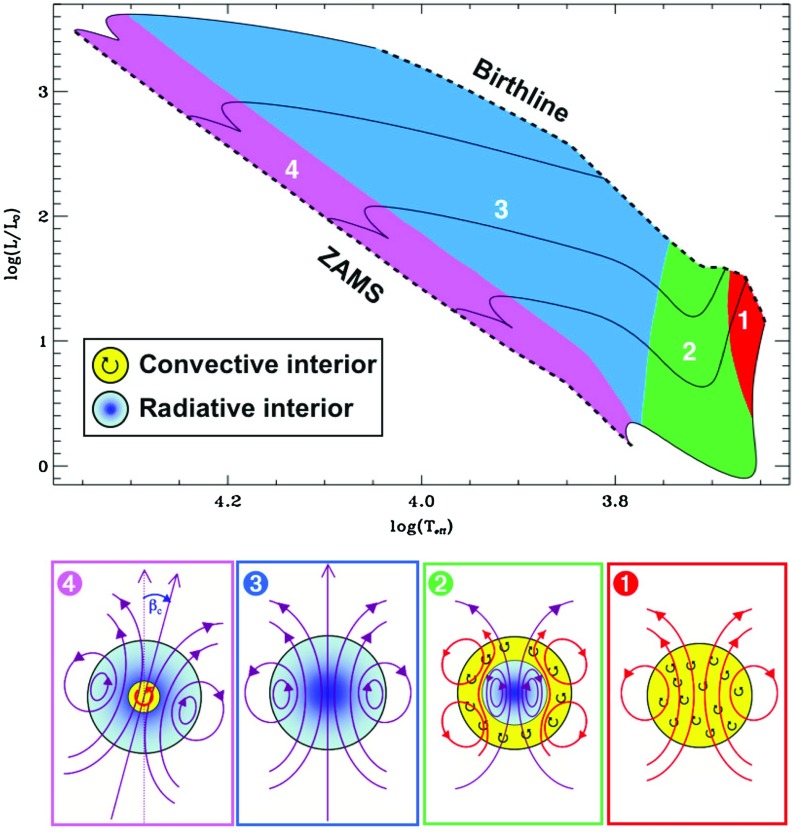
*Top* pre-main sequence stellar evolution track (*solid lines*) for respectively 1.2, 2, 3, 5 and 8 $M_{\odot }$ stars (using the Ceasam code Morel 1997), adapted from Behrend and Maeder (2001) to define the birthline. The *colored areas* correspond to the stellar structure shown on the *bottom part* of the figure. Note that lower-mass stars are not shown in this figure. Image reproduced by permission from Alecian (2014), copyright by the authors

As they descend the Hayashi track, from the birthline toward the zero age main sequence (ZAMS), stars undergo drastic changes in many aspects: among them size, internal structure, rotation rate, magnetic activity, and connection to their surroundings (see Fig. 11). Stars with a final mass close to that of the Sun ($0.3< M_* < 1.2\, M_{\odot }$) go first through a fully convective phase; then a radiative core appears and grows in size. For more massive progenitor stars up to $\sim$4 $M_{\odot }$, the growing radiative core “takes over” the whole star; as they reach the ZAMS, a convective core has formed, yielding an internal structure opposite to that of solar-like star (i.e., convective core - radiative envelope vs radiative interior and convective envelope). Even more massive stars form as radiative stars and arrive on the ZAMS having a convective core as well. Young massive stars are named Herbig stars and differ significantly from T-Tauris (Alecian 2014). Changes in the interior structure appear to be reflected in the surface magnetism as well: for example, Saunders et al. (2009) found that as the radiative core grows, the number of periodically variable T Tauri stars diminishes. Later, using surface magnetic maps of accreting T Tauri Stars (e.g., Donati 2013; Hussain and Alecian 2014), Gregory et al. (2012) found that stars with a massive radiative core possessed complex, non-axisymmetric surface fields with weak dipole components. In contrast, objects with smaller radiative cores ($0< M_{\mathrm{core}} < 0.4\, M_{\mathrm{star}}$) had less complex, more axisymmetric field geometries (though the dipole component was still typically weak).

One may further wonder what magnetic trace, or primordial field, is left from the initial intense formation phase of the star. Given the different structural evolution that these progenitor stars undergo as a function of their mass, it is expected that the rotation and magnetic field that they harbor will also vary. For massive stars, there is the well-known A-gap: only about 10% or so of these stars possess a magnetic field on the main sequence; these are named CP stars (Donati and Landstreet 2009). This field is intense, often oblique with respect to their rotation axis (see Mestel 1999); it is generally thought that a fossil origin for this field is most likely (Moss 2003), as discussed further in Sect. 5.4. These massive stars also probably possess intense dynamo action in their core (Browning et al. 2004; Brun et al. 2005; Augustson et al. 2016), but it may be hidden by their extensive radiative envelope (see, e.g., MacDonald and Mullan 2004). For less massive stars, going through the T-Tauri phase, the situation is different. As the star undergoes an intense fully convective phase, the primordial field captured by the star as it contracts and forms has been reprocessed so efficiently that it is likely forgotten (Moss 2003), see also Emeriau-Viard and Brun (2017). On the main sequence these stars show a contemporary magnetic field that is continuously generated by dynamo action (see Sect. 4.3).

Young solar-like stars tend to be fast rotators and this has a direct impact on the level of their magnetic activity (Feigelson and Montmerle 1999). Using spectropolarometric techniques (Petit et al. 2008) (and later work, see Morgenthaler et al. 2011; See et al. 2015) have further shown that along with more intense magnetic field amplitude, the geometry of the stellar magnetic field also changes.

**Fig. 12:**
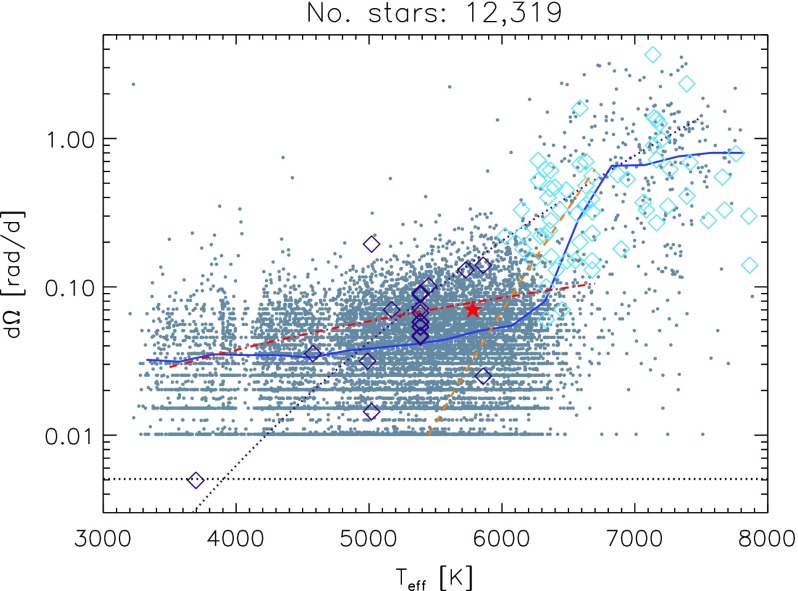
Differential rotation ${\varDelta } {\varOmega }$ in *Kepler*’s field stars. We note the systematic increase of angular velocity contrast with the effective temperature. *Grey dot* used *Kepler*’s light curves whereas *blue diamonds* used the fft technique of Reiners 2002. Some theoretical trends are superimposed (as in Küker et al. 2011) and show a positive slope with possibly a stronger dependency beyond 6000 K as in global stellar convection simulations. Image reproduced by permission from Reinhold and Gizon (2015), copyright by ESO

### Main sequence solar-like stars

By comparing the Sun to other solar-like stars, we hope to distinguish between behavior that is generic (i.e., common to all solar-like stars at different stages in their evolution) and that which is peculiar to the Sun. Observations of the rotation and magnetism of solar type stars, in particular, have revealed many interesting trends that inform us about the underlying physical mechanisms at work and how they are coupled. Of course, such trends share common properties with those discussed in the previous section regarding young solar-type stars.

As a prominent example of such trends, surface differential rotation is found to increase with $T_{\mathrm{eff}}$ as shown in Fig. 12; i.e., F-stars possess a larger latitudinal contrast than K-stars (Barnes et al. 2005; Reinhold and Reiners 2013; Reinhold and Gizon 2015):

$$\begin{aligned} {\varDelta } {\varOmega } \propto T_{\mathrm{eff}}^{8.92\pm 0.31}\, \end{aligned}$$

This suggests that the energy input at the base of the stellar convection zone as well as the thickness of the convective surface layer must both play a role in the way angular momentum is being redistributed in stars, as more luminous stars with shallower convection zones exhibit larger contrasts. Similar trends are found in numerical simulations and mixing length theory as explained in (Brun et al. 2017).

Another obvious dependency one could expect for the angular velocity contrast is its sensitivity to the rotation rate of the star ${\varOmega }_*$. Surprisingly, there is no overall agreement on the dependency of ${\varDelta } {\varOmega }$ with rotation rate as of today. Traditionally such dependency is written ${\varDelta } {\varOmega } \propto {\varOmega }_*^n$, with *n* a positive exponent. Indeed, in Donahue et al. (1996), Messina and Guinan (2003), Saar (2011), $n\sim 0.6{-}0.7$, in Hall (1991) and Henry et al. (1995), $n=0.24\pm 0.06$ and in Barnes et al. (2005) and Collier Cameron (2007), $n=0.15\pm 0.1$. Recent studies using asteroseismic data, have found a value in between $n\sim 0.3$ (Reinhold and Gizon 2015). One explanation for this spread could be that *n* depends on stellar spectral type as discussed in Balona and Abedigamba (2016). What we can conclude from these observational studies is that the relative contrast of differential rotation ${\varDelta } {\varOmega }/{\varOmega }_*$ in stars is expected to decrease with rotation rate, as *n* is always lower than 1.0, and to increase with stellar mass.

Not only the amplitude of the angular velocity is expected to change but also its profile. As we will discuss in Sects. 5 and 6.3, various states of differential rotation are likely to exist in the convective envelope of solar like stars (Brun et al. 2017). Recent observational attempts have tried to distinguish between prograde (solar) and retrograde (anti-solar) states of differential rotation (Reinhold and Arlt 2015).

Similarly, rotation-activity relationships have been observed for decades. As described in Sect. 4.1, many of the most significant results on long-term stellar activity have emerged from the decades-long Mount Wilson Observatory (MWO) Calcium (Ca) H+K Project (Wilson 1978; Noyes et al. 1984a; Baliunas et al. 1985; Hall 1991; Soon et al. 1993; Baliunas et al. 1995; Hempelmann et al. 1995; Pizzolato et al. 2003; Wright et al. 2004; Böhm-Vitense 2007; Mamajek and Hillenbrand 2008; Wright et al. 2011; García et al. 2014; Oláh et al. 2016). As noted earlier, emission in these lines is a proxy for the non-thermal heating of the chromosphere, and so long-term variation of the Ca H+K index is related to variability in the stellar magnetic fields (see review in Hall 2008). Complementary surveys, notably including Lowell Observatory’s Solar-Stellar Spectrograph program, have provided further insights (Hall et al. 2007; Hall 2008; Hall et al. 2009, see also Mamajek and Hillenbrand 2008). Measurements of the coronal X-ray flux (also described in Sect. 4.1) likewise reveal strong correlations between rotation and activity (Hempelmann et al. 1996; Micela and Marino 2003; Güdel 2004). For recent surveys of activity in solar-like stars, see for example Wright et al. (2004), Giampapa et al. (2006), Saar (2011), Marsden et al. (2014) or do Nascimento et al. (2014) and references therein.

Broadly, it is found that more rapidly rotating stars possess a higher level of magnetic activity, as already discussed in Sect. 4.2 in the context of young stars. Such observations, often based on X-ray luminosity and normalized to the bolometric luminosity of the stars, reveal that there is systematic increase of $L_X$ up to a rotation rate threshold (and/or Rossby number) beyond which it levels off, forming a “saturation” plateau. The Rossby number ($\sim$0.1–0.3) at which this plateau occurs seems almost independent of the mass of the solar-like star (given plausible assumptions about the bulk convective overturning time within stars of different masses); if viewed in terms simply of rotational velocity instead, stars of different masses “saturate” at different rotational velocities. The mechanism behind this leveling-off of activity is still being debated; plausibly, it could arise either due to the coverage by many or large starspots on the stellar surface, or through the field amplitude itself through a saturation of the dynamo mechanism, or both (Gondoin 2012; Reiners et al. 2014; Brun et al. 2015b).

**Fig. 13:**
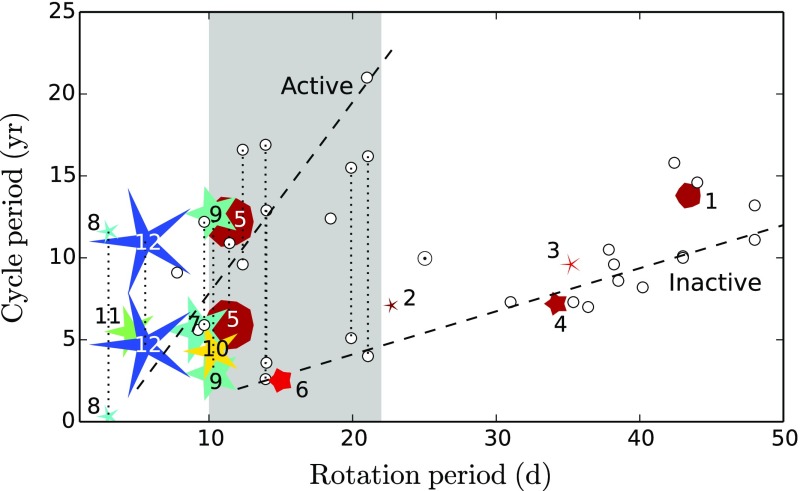
Magnetic cycle period versus rotation period in solar-like stars. Two sequences are defined by Saar and Brandenburg (1999), Böhm-Vitense (2007) as relatively young, active A sequence (*upper dashed line*) and the older, less active I sequence (*lower dash line*). *Dotted vertical line* connected stars with 2 identified cycles. Image reproduced by permission from See et al. (2016), copyright by the authors

A more difficult question to answer is the existence of a simple relation between stellar rotation period and magnetic cycle period. For decades, such a relation between magnetic cycle and rotation periods has been searched for. In the HK survey (Wilson 1978; Noyes et al. 1984a; Baliunas et al. 1995; Lockwood et al. 2007), it is found that:

$$\begin{aligned} P_{\mathrm{cyc}} \sim P_{\mathrm{rot}}^k, \end{aligned}$$

with $$k \sim 1.0\pm 0.25$$.

A more sophisticated analysis reveals that at least two branches/scaling relations have been identified: an active one corresponding to relatively young stars and an inactive one corresponding to slow rotators (Saar and Brandenburg 1999; Saar 2002; Böhm-Vitense 2007; Hall et al. 2009; Oláh et al. 2009; Metcalfe et al. 2010a; Saar 2011; See et al. 2016). There are illustrated in Fig. 13 and can be interpreted in various ways, for instance as the proof of multiple cycles in stars or as different state of activity level (normal vs grand minima phases). Recent studies have reanalyzed the HK survey and incorporated new surveys of long term monitoring of stellar magnetism, and question whether the relationship between $P_{\mathrm{cyc}}$ and $P_{\mathrm{rot}}$ and the existence of two distinct activity branches are robust Reinhold et al. (2017), Egeland (2017). While chromospheric activity is a well known proxy for assessing magnetic activity levels some evidence (such as with the Sun) indicate that it may not be as good for determining magnetic cycle period. Differences between chromospheric and magnetic cycle periods could however be due to different temporal sampling of the activity (See et al. 2016). Note also that activity variability and intensity in stars may depend on whether the stellar surface is spot or faculae dominated (see e.g., Shapiro et al. 2014).

One of the main difficulties here is that useful information about stellar magnetic cycles can only be obtained through long-term (e.g., multi-decadal) observations of stellar activity. Direct detections of spots on stellar surfaces have only recently become available; thus, no systematic analysis has yet been possible of the dynamics of these spots over periods of time long enough or a sample of stars large enough to constrain cyclical behavior see (Berdyugina 2005) for a first account of the results obtained with such methods). Nevertheless, proxies of magnetic activity cycles (or their absence) can be derived through different observational techniques, as discussed in Sect. 4. The most commonly used methods for assessing the existence of stellar cycles have been photometric and synoptic stellar chromospheric activity observations, together in some cases with stellar coronal X-ray variability data. Over the last decade, asteroseismology—via the influence of magnetism on acoustic mode frequency—has also became a very useful and complementary techniques to do so (see below and García et al. 2010; Brun et al. 2015b).

**Fig. 14:**
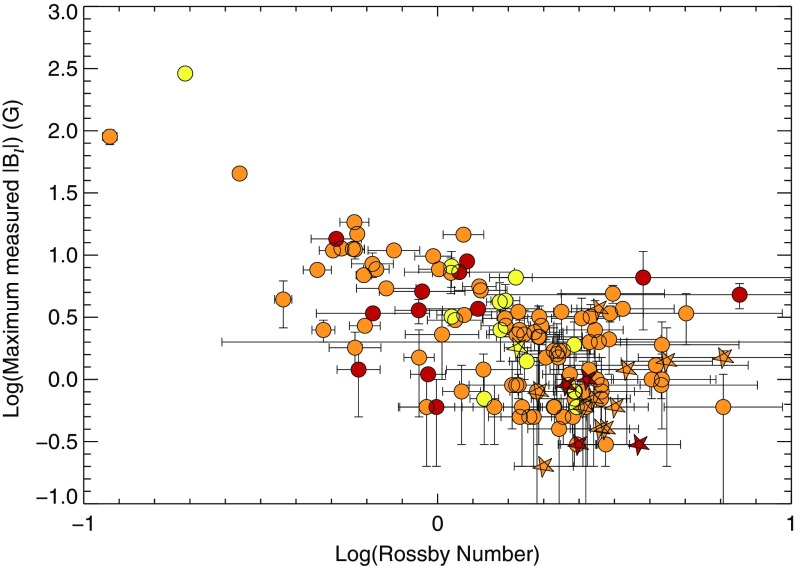
Magnetic field amplitude ($\log 10$) as a function of Rossby number ($\log 10$) in solar-like stars and subgiants as obtained with the Bcool survey. Stars respectively in *red* have $T_{\mathrm{eff}} < 5000\,\hbox {K}$, in *orange* have $5000 \le T_{\mathrm{eff}} \le 6000\,\hbox {K}$ and in yellow have $T_{\mathrm{eff}} > 6000\,\hbox {K}$. *Filled circles* represent dwarf stars and stars represent subgiants. Image reproduced by permission from Marsden et al. (2014), copyright by the authors

More recently, various authors—including the “Bcool” collaboration—have begun to map, and to follow over many years, the surface magnetism of solar-like stars (Marsden et al. 2014) as shown in Fig. 14, using the techniques of spectropolarimetry. Solar analogues have shown interesting trends in terms of field amplitude and geometry versus age (see Petit et al. 2008). These observations suggest that the faster the star rotates, the more its field geometry is dominated by its toroidal component and (as with young stars) the more intense is the field amplitude (See et al. 2015; Folsom et al. 2016).

In some stars, such as 61 cygni A, magnetic field polarity reversals have been observed in the polar cap region (Morgenthaler et al. 2011) and over the whole surface Boro Saikia et al. (2016). As with rotation (Barnes 2003; Barnes and Kim 2010; García et al. 2014), magnetic field intensity can be used to have a first estimate of stellar ages, e.g., the so-called *magnetochronology* (Vidotto et al. 2014a; Folsom et al. 2016). In this work the authors propose that $|B_v| \propto t^{-0.655\pm 0.045}$ or $|B_v| \propto R_{os}^{-1.38\pm 0.14}$ (Saar (2001), finds a slightly smaller exponent $-1.2$). This scaling is compatible with Skumanich’s law.

However, over the last couple of years there has been some debate regarding the existence of such scaling dependency of rotation with age for stars older than the Sun (Meibom et al. 2015; van Saders et al. 2016), the latter advocating that the Skumanich-style spin down law breaks down. This work is based on detailed analysis of *Kepler* light curves and asteroseismic age determinations (Metcalfe et al. 2014). The origin of this break is argued to be due to a change of properties of stellar magnetism resulting in a less efficient wind braking (see Sect. 5.6).

Using asteroseismology techniques on high cadence stellar photometric light curves from COROT and *Kepler* satellites, it has been possible to develop magnetic activity proxies (García et al. 2010). Since then, these have been calibrated and used jointly with activity *S* index to constrain magnetic-activity modulations in solar-like stars (Mathur et al. 2013; Salabert et al. 2016a) (see also Saar (2011), Salabert et al. (2016b)). Metcalfe et al. (2016) using Kepler photometric light curve advocate for a change of dynamo regime near the solar Rossby number. Reinhold et al. (2017) are finding new trends for the magnetic cycle-rotation period relationship. Likewise, super flares have also been detected on solar-like stars by detailed analysis of the *Kepler* data by Maehara et al. (2012) see also Living Review by Shibata and Magara (2011).

Stellar activity of solar-like stars can also exhibit well identified activity clusters. Swift 180$^\circ$ change of longitude, known as the flip-flop phenomenon have been observed (Berdyugina 2005). This effect appears to be more prominent in young active stars than on moderately active ones such as the Sun.

In Sect. 6.3, we will discuss how in a classical $\alpha -\omega$ dynamo (and in the equivalent flux transport Babcock–Leighton alternative) there exists a simple link between the Rossby number and the Dynamo number *D*, that can explain the observed positive linear scaling between rotation period and magnetic cycle period (e.g., Durney and Latour 1978; Noyes et al. 1984b; Baliunas et al. 1996; Tobias 1998; Montesinos et al. 2001; Jouve et al. 2010). We will also discuss a subset of recent simulations that show that the magnetic cycle length could also increase rather than decrease with shorter rotation period (Jouve et al. 2010; Strugarek et al. 2017).

### Lower-mass stars

#### Introduction

Most stars in our galaxy are smaller than the Sun. About 70% by number are M-dwarfs, stars ranging in mass from about 0.5 to around 0.08 solar masses (on the main sequence) and in luminosity from $\sim$0.1 $L_{\odot }$ to less than $10^{-3}\, L_{\odot }$ (e.g., Chabrier and Baraffe 1997; Reid and Hawley 2005). From an astronomical perspective, these stars are interesting partly because they are so common: in their spatial distribution we can discern the influence of dynamical heating in the Galactic disk (e.g., West et al. 2006); in their signatures in the integrated light of other galaxies, some authors have suggested evidence of variations in the initial mass function (van Dokkum and Conroy 2010). These stars have also become major targets in the search for “Earth-like” exoplanets (see, e.g., Tarter et al. 2007; Scalo et al. 2007; Berta et al. 2012). With this has come an appreciation of the magnetic activity in such stars: because, for example, the “habitable zone” in these stars is likely to be comparatively close in (e.g., Pierrehumbert 2010; Haswell 2010), it is conceivable that magnetic activity in these stars would exert an especially great influence on the environment of any orbiting planets (e.g., Lammer et al. 2007; Walkowicz et al. 2008).

From a dynamo theorist’s perspective, these stars also hold special interest: the convection zone in main sequence stars deepens with decreasing stellar mass, and by a mass of about 0.35 $M_{\odot }$, stars are thought to be convective throughout their interiors (e.g., Kippenhahn et al. 2013; Chabrier and Baraffe 1997). (This transition occurs at a spectral type of about M3-M4.) Because the Sun’s global-scale magnetic field has long been thought to be built partly at the interface between the convection zone and the radiative interior (see Sect. 5), M-dwarfs may thus provide a powerful constraint on theories of the field generation. If the presence of such an interface is crucial in establishing the strength and character of a star’s magnetism, one would expect stars without such an interface to show markedly different magnetism than stars that possess one. Indeed, it was once common to assume this would be the case (e.g., Durney et al. 1993). In this section, we review observations of magnetism in low-mass stars, aiming partly to assess whether and how this differs from what is realized in stars like the Sun. A more detailed summary is provided in the recent review by Reiners (2012).

#### Observational challenges and summary

First, though, it is worth noting why observations of magnetism in M-dwarfs are comparatively difficult. Most obviously, they are dim: a fully convective M-star emits at most about a hundredth as much light as the Sun, so capturing (for example) a high-resolution spectrum that could be examined for some of the signatures of magnetic activity described above (e.g, the Ca ii H and K lines) can require long integrations even on the world’s largest telescopes (e.g., Delfosse et al. 1998; Marcy and Chen 1992; Browning et al. 2010; Reiners et al. 2012). Furthermore, other tracers can be difficult to interpret in M-stars: e.g., with increasing magnetic activity the H$\alpha$ line can appear first in absorption, then display an emission core, and only at higher activity appear as a strong emission line (see, e.g., Cram and Mullan 1979; Reid and Hawley 2005). Hence, low-resolution spectra that do not show H$\alpha$ can reflect either no activity or a moderate amount (i.e., enough to have an emission core in the absorption line).

Despite these difficulties, many interesting and surprising result on M-star magnetism have emerged in recent years. Broadly, many of these stars are *highly active*, they appear (at some masses) to show evidence of a *rotation-activity correlation* similar to that in Sun-like stars, and there is evidence that the *spatial structure* of the field is different in stars with and without a radiative core. Below, we summarize each of these findings in turn.

**Fig. 15:**
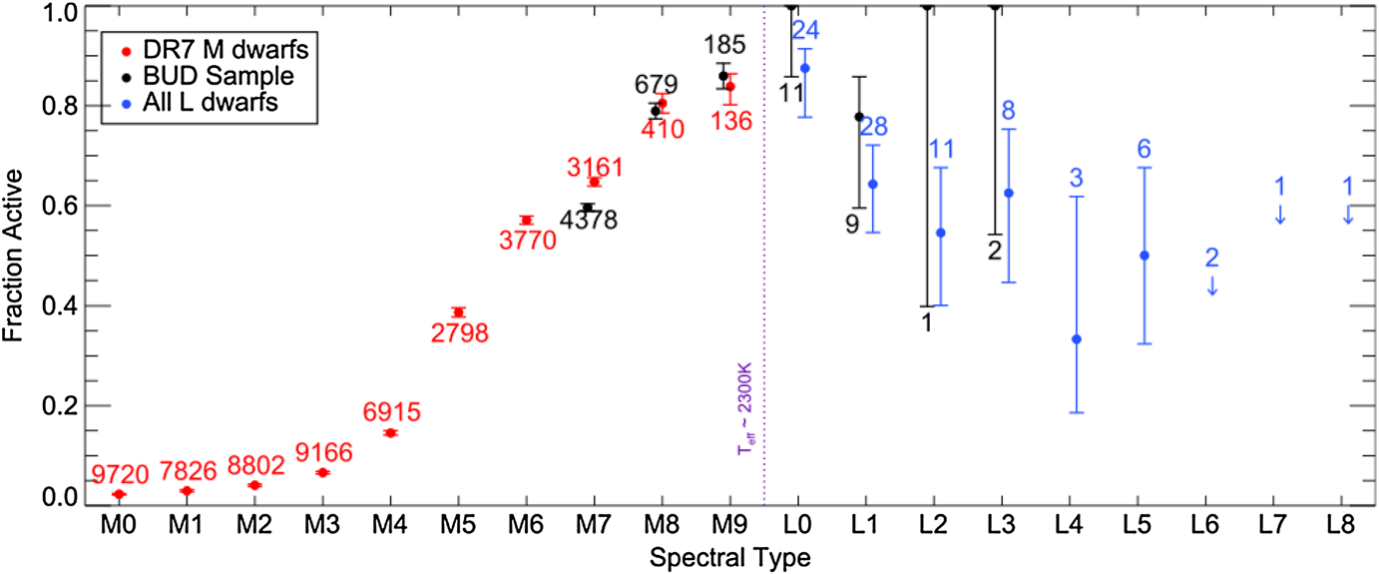
Fraction of low-mass stars and brown dwarfs showing chromospheric activity, versus spectral type. Measurable activity persists even to remarkably late types, and is extremely common in the fully convective (mid-M) regime. Image reproduced by permission from Schmidt et al. (2015), copyright by AAS

#### Many fully convective stars are very active

**Fig. 16:**
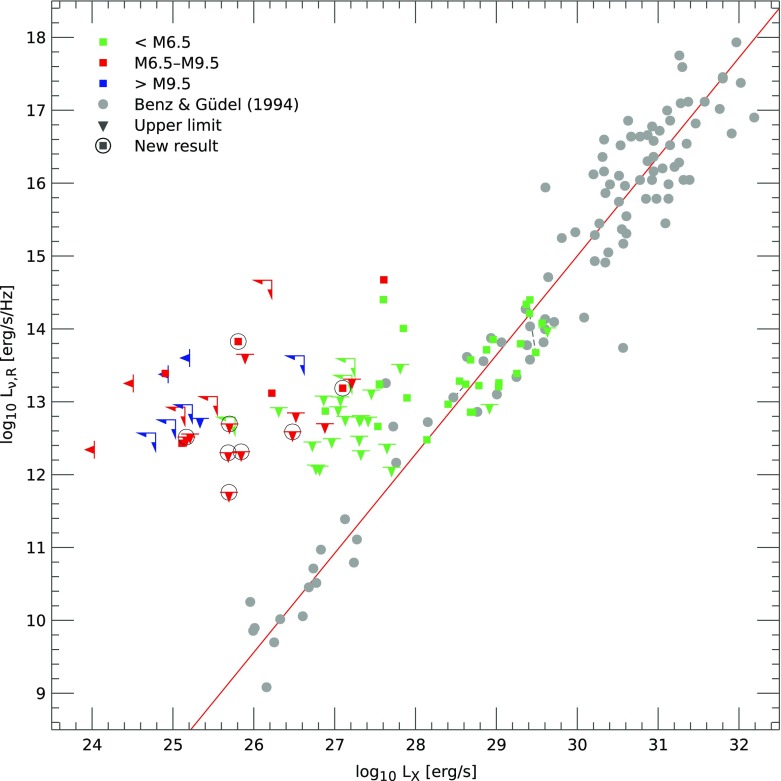
Radio versus X-ray luminosity in a sample of low-mass objects, showing the breakdown of the Güdel–Benz relationship. Image reproduced by permission from Williams et al. (2014), copyright by AAS

Zeeman broadening measurements have long suggested that the average surface field strength in some fully convective stars must be remarakbly high, of order a few kG (e.g., Johns-Krull and Valenti 1996, 2000; Reiners et al. 2009). The fraction of stars in this mass regime showing measurable H$\alpha$ emission increases with decreasing stellar mass, with (for example) 80–90% of late-M dwarfs exhibiting activity (Schmidt et al. 2015). Although the overall *level* of activity (as measured by traditional indicators like $L_{H \alpha }/L_{\mathrm{bol}}$, the ratio of the luminosity in the $\hbox {H}\alpha$ line to the bolometric luminosity) declines with decreasing mass below about spectral type M7, measurable activity persist to very low masses (late types). Many studies have suggested that the activity *fraction* also declines in the late-M/early-L regime (e.g., Gizis et al. 2000; West et al. 2004); for a time there was an especially pleasing concordance between these observations and theoretical models of ultracool atmospheres (Allard et al. 2000; Mohanty et al. 2002), which indicated that activity should fall off in the late-M regime, essentially because the outer layers of these stars become so cool (and hence probably poorly ionized), that they can no longer support magnetic stresses that ultimately drive chromospheric activity. The view today is slightly more complicated, but it still seems fair to say that magnetic fields and chromospheric emission do not trace each other as well in this regime because of the growing atmospheric neutrality. Some previous estimates of the activity fraction were influenced by the difficulty of detecting weak H$\alpha$ emission in these objects; the latest results (Schmidt et al. 2015), as sampled in Fig. 15 suggest that chromospheric emission persists to very low masses indeed: fully half of early L-dwarfs in this sample show emission. Ultracool dwarfs also exhibit strong radio emission (Hallinan et al. 2008; Berger et al. 2010; Williams et al. 2014), likewise indicating that strong surface magnetic fields persist even when obvious chromospheric or coronal emission does not. The radio emission is in some cases vastly stronger than would be anticipated on the basis of their coronal activity (as encapsulated by the Güdel–Benz relation, Guedel and Benz 1993; Benz and Guedel 1994), as displayed in Fig. 16 (taken from Williams et al. 2014). Some ultracool dwarfs exhibit periodic, bright radio pulses (e.g., Hallinan et al. 2006, 2008; Berger et al. 2009). This led Hallinan et al. (2006) and other authors to suggest that these objects host electron cyclotron maser emission, arising from low-density regions in the magnetospheres of these objects and more akin to Jupiter’s decametric radio emission than to classical stellar chromospheric activity. On the theory side, it is also increasingly clear that the ionization of ultracool stellar atmospheres (which in turn influences the degree to which magnetic fields can drive activity) is affected by a variety of different processes, which may contribute to maintaining ionized electrons even when surface temperatures are very cool; see the series of papers by Helling and collaborators for much more detail (Helling et al. 2011b, a, 2013; Rimmer and Helling 2013; Stark et al. 2013; Bailey et al. 2014), and the recent review by Helling and Casewell (2014).

#### Some correlations between rotation and activity persist

**Fig. 17:**
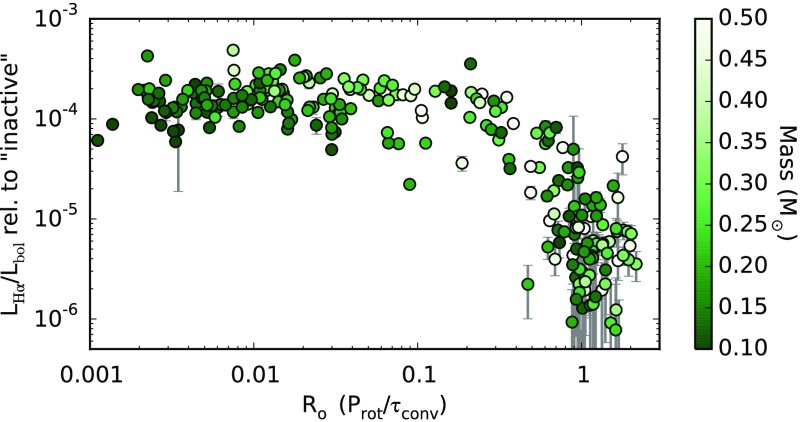
Luminosity in H$\alpha$ (a measure of chromospheric activity) versus Rossby number (an estimate of rotation rate; more rapid rotation rate is to the left) in a sample of M-dwarfs, exhibiting a rotation-activity relation. The H$\alpha$ luminosities are normalised to the bolometric luminosity, using equivalent widths measured relative to the maximum absorption level for M-dwarfs of the same mass. Image reproduced by permission from Newton et al. (2017), copyright by AAS

In solar-type stars, the well-established link between magnetic activity and rotation rate provides a profound constraint on dynamo models (see, e.g., Sect. 3 and Noyes et al. 1984a). Many studies have attempted to determine whether this relation persists in the fully convective regime (e.g., Mohanty and Basri 2003; Reiners and Basri 2008; Reiners et al. 2009; Browning et al. 2010; Reiners and Basri 2010; Reiners et al. 2012; McLean et al. 2012). In portions of this mass regime, this analysis is complicated by the fact that the “rising” part of the rotation-activity correlation would, if it occurred at the same Rossby numbers as in more massive stars, correspond to rotational velocities below those that can usually be detected by Doppler broadening of spectral lines. Put another way, rotation is probably dynamically strong in any M-dwarf whose rotation is measurable via spectroscopy. (See Sects. 5, 6 for a discussion of why this is so.) Because of this, some of the best constraints have come from studies incorporating *photometric* rotation periods, since in principle these can probe even very slow rotation rates. Broadly, we would summarize these observations as indicating that rotation continues to be linked to activity well into the fully convective (mid/late-M) regime (e.g., Reiners et al. 2009; Reiners 2012; West et al. 2015; Wright and Drake 2016; Newton et al. 2017). One view of this is provided by Fig. 17, from Newton et al. (2017), which presents estimates of chromospheric activity (using the H$\alpha$ line) as a function of rotation in a sample of M-dwarfs. There is a clear rise in activity with increasing rotation rate in the slower rotators, and a “plateau”, just as in solar-like stars, at more rapid rotation. Complementary views using other proxies of magnetic activity can be found elsewhere—see, e.g., the review of Reiners (2012) for an example using the FeH bands, and the recent paper of Wright and Drake (2016) for X-ray measurements—with broadly equivalent results.

#### Spatial structure of the fields

The spatial distribution of the magnetism can be probed to some extent using the technique of Zeeman Doppler Imaging (as described above and in, e.g., Donati et al. 1997 and the review of Donati and Landstreet 2009), and by comparing the results from this method to measurements of traditional Zeeman broadening (e.g., Johns-Krull and Valenti 1996; Reiners and Basri 2009). Prominent results include those presented in Donati et al. (2006a, 2008), Morin et al. (2008, 2010), Rosén et al. (2015), and a recent review is provided by Linsky and Schöller (2015). Broadly, the Zeeman Doppler Imaging results suggest that some fully convective stars possess large-scale fields of remarkable ($\sim$kG) strength, whose measurable structure is evidently (in the mid/late-M regime) mostly axisymmetric and poloidal. The ZDI measurements show a fairly abrupt transition from predominantly toroidal (azimuthal) fields to poloidal ones at around the same as the transition to full interior convection. Comparison of the signed flux measured in circular polarization data (i.e., the net signal surviving after cancellation of oppositely-oriented fields) and the unsigned flux (as measured by magnetic broadening of spectral lines) suggests, though, that small-scale magnetism (largely unprobed by ZDI) accounts for the vast majority of the magnetic energy (see, e.g., Reiners and Basri 2009). Whereas in more massive stars, the ZDI-inferred geometry of the field appears to depend only on stellar mass and rotation rate (i.e., stars of the same *M* and ${\varOmega }$ seem to give similar results), this may no longer be true in the lowest-mass objects: e.g., Morin et al. (2010) found that some late-M stars had strong, axisymmetric dipolar fields whereas others (at similar masses and rotation rates) hosted weaker, non-axisymmetric fields. Some have interpreted this as evidence for “bistability” in the dynamo process, described in more detail in Sect. 6 (e.g., Morin et al. 2011; Gastine et al. 2013a), though others have suggested that cyclical modulations between strong and weak-field states may be occurring (Kitchatinov et al. 2014).

#### Possible impact of magnetism on structure

As a final twist, we briefly note that there is evidence (from measurements of radii in eclipsing binaries) that some active M-dwarfs have larger radii than standard 1-D models would predict (see, e.g., Torres and Ribas 2002; López-Morales 2007; Morales et al. 2008; Stassun et al. 2012; Torres 2013). Several authors have examined the possibility that this might arise partly from the influence of strong magnetic fields, which could modify the convective heat transport; see summary in Browning et al. (2016). For example, Mullan and MacDonald (2001) explored the possibility that some of the observed properties of these stars might arise if the interior was not fully convective but instead possessed a small stable core, arising from the stabilizing influence of a sufficiently strong magnetic field. The fields required to completely stabilize the interior are up to 100 MG; somewhat less extreme fields have been examined in several later papers (e.g., Chabrier et al. 2007; MacDonald and Mullan 2012, 2013, 2014, 2015; Feiden and Chaboyer 2012, 2013, 2014), modeled using various forms of mixing-length theory (accounting for the influence of magnetism either by simply reducing the mixing-length parameter $\alpha$ or through more complex prescriptions). Within the mixing-length prescriptions adopted by MacDonald and Mullan (2014) or Feiden and Chaboyer (2014), the fields required to yield significant radius inflation would be quite strong, typically 1 MG or more at some regions within the interior; Feiden and Chaboyer (2014) ultimately find such fields to be unlikely, whereas MacDonald and Mullan (2014) are more sanguine about their prospects. Clearly such fields are far in excess of the value that would be in equipartition with the convection (typically a few kG, assuming MLT estimates for the convective velocity are roughly correct), though they could plausibly be in equipartition with, for example, the kinetic energy of internal shear flows that are difficult to constrain observationally (as argued by MacDonald and Mullan 2015). Recently Browning et al. (2016) have argued that, even with fairly generous assumptions about the efficacy with which fields can be regenerated, no internal fields stronger than $\sim$800 kG are consistent with the combined constraints of both magnetic buoyancy and Ohmic dissipation. Stronger fields tend to rise via buoyancy more rapidly than they could plausibly be regenerated (or “pumped” downwards by the convection), unless they are structured on very small spatial scales; but fields on such small scales would necessarily be accompanied by large current densities (the current density *j* scales roughly as *B*/*a*, with *a* a characteristic spatial scale of the magnetism), and the Ohmic dissipation associated with this would, in extreme cases, greatly exceed the luminosity of the star. The maximum “allowed” field scales with stellar mass, since it is essentially a multiple (set by the conductivity of the object and its luminosity) of the equipartition field. A principal limitation of the Browning et al. (2016) model is its assumption that a variety of basic results derived within the so-called “thin flux tube approximation” carry over to more general field configurations as well; clearly this is only an approximation, the boundaries of which have yet to be tested. What this all implies for the “inflated” radii of low-mass stars is not yet clear: plausibly (assuming the measured radii are accurate) either other phenomena must act to increase the radii, or perhaps weaker magnetism (coupled with rotation or tidal effects) is sufficient to affect the convective transport and hence the radius. As of this writing the issue is still unresolved.

### More massive main-sequence stars

Just as M-dwarfs dominate the stellar mass of our Galaxy, stars more massive than the Sun dominate its light. (The number of stars per unit mass interval increases with decreasing mass according to a power law—see, e.g., Chabrier 2003—but meanwhile the luminosity varies with mass even more steeply, $L \propto M^{3.5}$ or so in this mass range.) It is these stars that are largely responsible for the chemical evolution of galaxies with time: many O and B stars have come and gone since the first Population III stars, enriching the ISM with each passing generation; in contrast, not a single M-dwarf has yet passed off the main sequence. The evolution of these stars, and in particular the end stages of their lives, may be profoundly affected by rotation and magnetism. Some especially luminous supernovae, for example, may be powered partly by radiation from magnetars (e.g., Woosley 2010), neutron stars with magnetic fields >10$^{14}$ G, which in turn are generally thought to result partly from rapid rotation in the collapsing iron cores of massive stars (Duncan and Thompson 1992; Thompson and Duncan 1993). These end stages of stellar evolution are outside the scope of this review; we mention them here only because they lend special vibrancy to the study of the massive stars that are the progenitors of these objects. In this section, we briefly summarize some major observational findings regarding rotation and magnetism in such stars. A more comprehensive review focusing on magnetism in this mass range can be found, for example, in Walder et al. (2012), and we again refer frequently to (Donati and Landstreet 2009) as well.

#### Convective cores, radiative envelopes and the presence of coronae

A basic result of stellar structure theory is that with increasing stellar mass, the convective envelope becomes shallower: while the convection zone of the Sun occupies about the outer 30% of the star by radius, stars of 2 solar masses (i.e., mid-A-type stars) have outer convection zones of negligible extent and even more negligible mass. In some massive stars there are multiple thin convective envelopes, driven partly by the opacity peaks of different elements: see, e.g., Wolff (1983) for a review specific to A-type stars, and Cantiello et al. (2009) for recent calculations of the properties of convection driven by the iron opacity peak in more massive stars. (It should perhaps be noted that “thin” is a relative term here: the Fe convection zone in the models of Cantiello et al. 2009, for example, extends for a significant fraction of a solar radius! But this is in the context of a overall stellar radii of order 10–20 $R_{\odot }$.) As the convective envelope shrinks in extent, though, a convective core develops: by the time surface convection zones have nearly disappeared in the mid-A stars (e.g., Robrade and Schmitt 2009), the convective core occupies the inner $\sim$15% of the star by radius. These changes in structure are a consequence of changes in the nuclear energy generation and opacity of the material at varying temperature and density (as discussed in Sect. 5).

The disappearance of prominent surface convection zones has observable consequences for the magnetism of these stars. Stellar coronae and transition regions fade away in this mass range, as probed by UV and X-ray observations (see, e.g., Vaiana et al. 1981; Pallavicini et al. 1981; Schmitt et al. 1985; Rosner et al. 1985; Güdel 2004; Robrade and Schmitt 2009). The interpretation is that at spectral types between roughly B8 and A7, there is not enough non-radiative heating to heat the atmosphere to the temperatures required for such emission; such heating in solar-type stars is linked to surface convection and magnetism, so its absence is consistent with the disappearance of prominent near-surface convection zones. The O and B stars often show X-ray emission as well, but this is thought to arise primarily from shocks forming in the massive, radiatively driven winds from these hot stars (see, e.g., Owocki et al. 1988; Lucy and White 1980; Townsend et al. 2007).

#### The Ap/Bp phenomenon

**Fig. 18:**
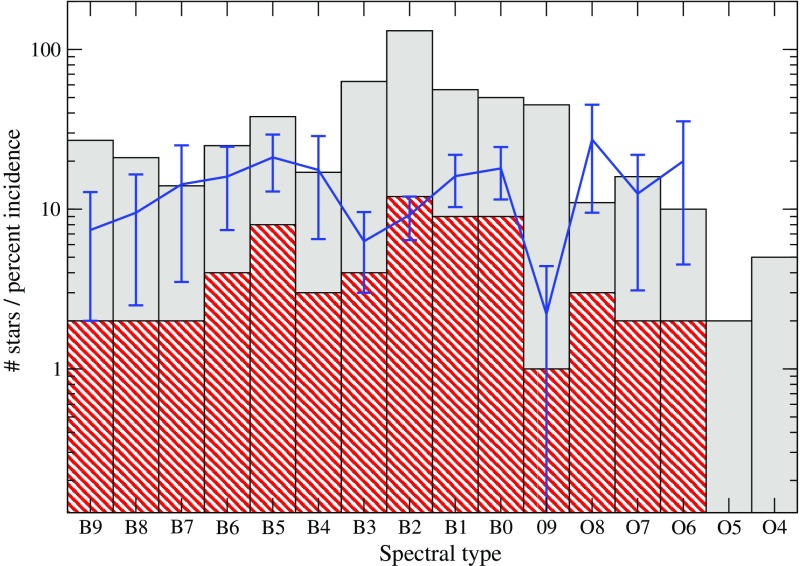
MiMeS survey of magnetic field occurrence in massive stars, showing the percentage in each mass bin for which magnetic fields were detected, together with numbers of stars in each bin. Image reproduced by permission from Wade et al. (2014), copyright by IAU

It has been known for more than a century that some A-type and B-type stars exhibit chemical “peculiarities”, typically involving unusual abundances of Si or rare earth elements (see, e.g., Wolff 1983 for an extensive review). Since the initial discovery of a magnetic field in one of these stars (Babcock 1947), it has become clear that all stars showing these chemical anomalies, known as Ap or Bp stars, also appear to be magnetic (see, e.g., Landstreet 1992, and again the review by Donati and Landstreet 2009), whereas measurable magnetic fields are absent in “normal” main-sequence A and B stars. The fraction of stars showing these abundance anomalies and magnetism varies somewhat with spectral type (e.g., Power et al. 2008); in all cases, less than 10% of A/B stars exhibit these phenomena. Broadly similar incidence rates are found in the (more massive) O stars as well. As an example of this, in Fig. 18 (taken from Wade et al. 2014) we display the number (and incidence rate) of O and B stars in the “MiMeS” (Magnetism in Massive Stars) survey displaying observable magnetism, as measured using spectropolarimetry.

**Fig. 19:**
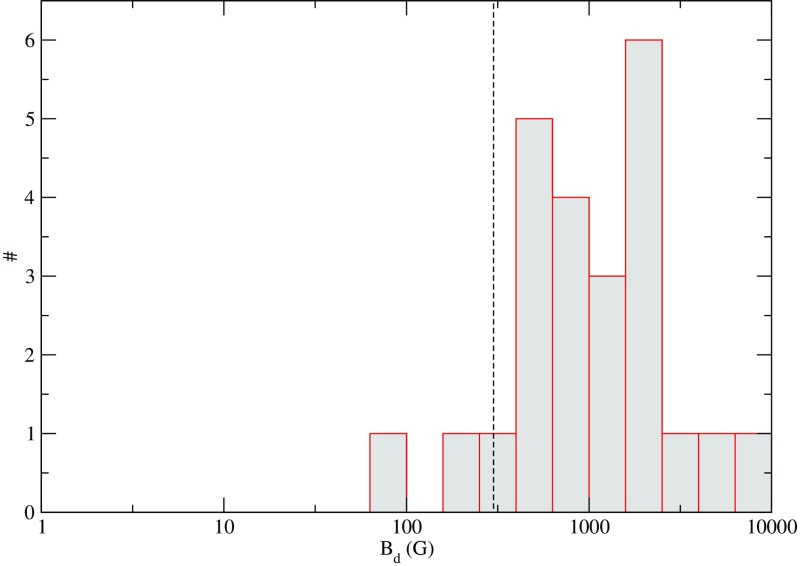
Histogram of measured best-fit dipole magnetic field intensities in a sample of 28 Ap/Bp stars, taken to suggest a magnetic “desert” at weak field strengths. Image reproduced by permission from Aurière et al. (2007), copyright by ESO

The magnetic fields measured in Ap/Bp stars possess several striking properties that have likewise been the subject of observational scrutiny for decades, and which distinguish them from magnetism in less massive stars. Many more details can be found in, e.g., Mestel (1999), Borra and Landstreet (1980). First, the magnetism in some cases is remarkably strong: fields of more than 30 kG have been observed (e.g., Babcock 1960; Kochukhov 2006), with typical field strengths of order 2 kG. Remarkably, there appears to be a weak-field cutoff at around 300 G: i.e., no Ap star with a surface field weaker than this has been found, even though the detection threshold in some studies has been significantly lower than this (Aurière et al. 2007). The separation between the detection threshold and the 300 G lower-limit in some studies suggests that the low fraction of observably magnetic stars is not simply a selection effect. (See Sect. 5.4 for discussion of the physical significance of this result.) This is apparent in Fig. 19, taken from Aurière et al. (2007), which displays the number of detected stars at various field strengths in a sample of 27 Ap/Bp stars for which spectropolarimetric measurements were obtained using the MuSiCoS and NARVAL instruments. Second, the magnetism in these objects is, at a coarse level, often reasonably well-described by comparatively simple, large-scale field morphologies (see, e.g., discussions in Wolff 1983; Landstreet 1982). This is sometimes encapsulated in the “oblique rotator” model, which supposes that the observed field results from viewing a simple magnetic dipole of given strength, tilted at some angle with respect to the stellar rotation axis. A sketch of this model can be found in Stibbs (1950). As measurements have become more sophisticated, however, it has become clear that higher-order multipoles contribute to the field geometry as well (e.g., Bagnulo et al. 2001; Kochukhov et al. 2004). Third, the magnetic Ap stars *typically* rotate significantly more slowly than normal stars, with some exhibiting rotational variations on timescales of decades; that said, some Ap stars still rotate quite rapidly (in excess of 100 km s$^{-1}$). Within the subset of stars showing magnetism, there is no evident correlation between rotation and magnetic activity, in striking contrast to the “rotation-activity correlation” seen in solar-like stars (and discussed above). We defer a discussion of the interpretation of these remarkable features to Sect. 5.

## Origins of stellar activity

It is clear that stars possess the key ingredients needed for development of a dynamo (Weiss 1994). Turbulent motions are probably present in abundance, whether in convective regions or potentially also radiative ones (Spruit 2002); so too are large-scale shear (i.e., differential rotation), helicity imparted by rotation, and low diffusivity. All these attributes potentially favor the development of a magnetic field. The observable presence in the Sun and other stars of phenomena that are magnetically driven—including flares, spots, and coronal mass ejections—likewise suggests that magnetic fields are common in stars, and it is natural to suppose that in many cases these arise as a natural consequence of dynamo action. But many different types of activity are observed, and this might be taken to indicate the presence of different “types” of dynamo action. In this section, we discuss some of the main physical processes that give rise to stellar magnetism.

### Basics of convection and rotation

Central to the understanding of stellar magnetism is the description of turbulent rotating convection. This nonlinear physical process transports heat and energy, redistributes angular momentum to yield large scale flows such as differential rotation and meridional circulation, and ultimately creates, sustains and organizes magnetic fields on all scales (see Sects. 5.2–6). This subsection briefly reviews background material on rotating convection; readers who are already familiar with standard treatments of stellar or geophysical convection may skip this without detriment.

From intense surface convection envelopes in solar-like stars to deep convective cores in massive stars, convection plays a role in every main sequence star’s life. Hence in order to understand stars we must understand rotating convection. Convection is an instability occurring in a stratified fluid or plasma, which transports energy through the bulk displacement of a parcel of fluid. Many examples of convective flow are familiar from everyday life—the formation of large cumulo-nimbus clouds above the sea on a warm afternoon, or the motion induced in a pot of water set on a heat source. In these cases, as in stars, the central principle is that what is heavy (usually cooler) must come down, and what is light (usually hotter) goes up. The resulting overturning motion typically attenuates the vertical gradient of temperature in the bulk of the layer, e.g. between a hot source at the bottom and a cooler upper surface, by establishing thermal boundary layers to which most of the temperature variation is confined. In stars, which possess large overall density stratifications, the plasma’s entropy rather than its temperature is typically a more useful thermodynamic variable to consider for the characterization of convection efficiency: highly efficient convection tends to maintain a nearly adiabatic stratification.

These convective motions serve to carry out the nuclear energy generated in the core of a star. The location of the convection zone is strongly dependent on the stellar mass, driven by changes in the flux that must be carried, in the opacity, and in the ionisation state of the material. More specifically, solar type stars ($0.35< M_* < 1.8\, M_{\odot }$) have a radiative interior surrounded by a turbulent convective envelope, low mass stars (${<}0.35\, M_{\odot }$) are fully convective and hot stars (${>}1.8\, M_{\odot }$) have convective cores and an extended radiative envelope.

The integrated heat flux through the convection layer of a main sequence star is small relative to the whole thermal energy content of the plasma. Indeed, this high heat capacity is part of why only modest convective velocities and near-adiabatic stratifications prevail in most stellar convective zones: even these sorts of motions can carry a great deal of heat. Thermal energy may also be transported through conduction (heat flow from a hot region to an adjacent cold one, through microscopic collisions and electron flow) or radiation, though in stars the former is comparatively negligible. A useful way of characterizing the efficiency of convection is the Nusselt number, which essentially quantifies the convective transport relative to that by radiative diffusion or conduction; in stellar convection zones this number is generally “large”, with convection carrying most of the flux.

Given the large size of these celestial objects and the low atomic viscosity of their plasma, stars possess very high Reynolds numbers ($Re = UL/\nu$, with *U* a characteristic velocity, *L* a characteristic length, and $\nu$ the viscosity) even for weak characteristic velocities. (See discussion of this and other nondimensional numbers later in this section.) Hence their internal motions must be highly turbulent. Unfortunately, no comprehensive theory yet exists that can fully describe the complexity of turbulent nonlinear convective, rotating and magnetized systems (all stars rotate and most are magnetically active). Still, several approaches have been pursued to remedy the lack of a predictive theory for stellar convective zones (Spiegel 1971).

The approach used most in the community of stellar structure and evolution is the Mixing Length Theory (MLT), essentially as proposed by Böhm-Vitense (1958) (see also Cox and Giuli 1968; Schatzman and Praderie 1990; Hansen and Kawaler 1994), drawing partly on earlier work by (e.g.) Biermann (1932, 1945) and Prandtl (1925). It allows one to compute convective heat transport by assuming a simple hypothesis about convection. A convective blob (or parcel, eddy, cell, rising element) at an equilibrium position in a stratified atmosphere is displaced over a distance $\lambda _p$, the mixing length, before releasing its heat content. Usually $\lambda _p$ is assumed to be proportional to the local pressure scale height $H_p$, e.g., $\lambda _p = \alpha _{\mathrm{MLT}} H_p$, with $\alpha _{\mathrm{MLT}}$ the mixing length coefficient of order unity (not to be confused with the $\alpha$-effect of dynamo action discussed in Sect. 5.2). The appropriate value of $\alpha _{\mathrm{MLT}}$ depends on the specific variety of MLT adopted (see Gough and Weiss 1976, for discussion). In practice, accurate 1-D solar standard models are used to better determine its value (Brun et al. 2002; Turck-Chièze et al. 2004; Bahcall et al. 2005; Antia and Basu 2005, 2006; Asplund et al. 2009; Basu et al. 2015). Over the years several attempts have been made to calibrate $\lambda _p$ against 3-D numerical simulations of surface stellar convection (see for instance Abbett et al. 1997; Trampedach et al. 2014, and references therein). Typical mixing-length prescriptions do not take explicit account of rotation or magnetism, though efforts to include these effects in various ways have been made (e.g., Chabrier et al. 2007; MacDonald and Mullan 2015; Feiden and Chaboyer 2014).

In the inviscid limit ($\nu =\kappa =0$, no viscous nor thermal dissipative effects), the criteria for convective instability in a stratified atmosphere is well-known:

2 $$\begin{aligned} \left( \frac{d\ln T}{d\ln P}\right) _m > \left( \frac{d\ln T}{d\ln P}\right) _b + \frac{\varphi }{\alpha _t} \left( \frac{d\ln \mu _m}{d\ln P}\right) _m \end{aligned}$$

where $\alpha _p$, $\alpha _t$, and $\varphi$ are thermodynamic coefficients,^1^ $\mu _M$ is the mean molecular weight, and the subscripts *m* and *b* refer to the background medium and to a moving “blob” of fluid respectively. (Hansen and Kawaler 1994). Alternatively, using the classical gradient notation of stellar physics: $\nabla > \nabla _b + \frac{\varphi }{\alpha _t} \nabla _{\mu _M}$. This is the so-called Ledoux criteria for convective instability. If no variations of composition or ionization are assumed then the Ledoux criteria reduces to the Schwartzschild criteria: $\nabla > \nabla _b$, which in a stratified layer where energy is solely transported by radiation (e.g., $\nabla =\nabla _{\mathrm{rad}}$) and the fluid element is displaced adiabatically ($\nabla _b = \nabla _{\mathrm{ad}}$) becomes:

3 $$\begin{aligned} \nabla _{\mathrm{rad}} > \nabla _{\mathrm{ad}} \end{aligned}$$

In practice, even though atomic viscosity can be very small in a stellar plasma, the threshold to trigger convection will be higher than in the inviscid limit because diffusion will erode small perturbations. The instability criteria for a horizontal layer heated from below is described in detail by Chandrasekhar (1961) and many subsequent works have considered rotating spherical shells (Roberts 1968; Busse 1970; Gilman 1975). It is characterized by the so-called Rayleigh number *Ra*, which essentially measures buoyancy driving relative to viscous and thermal dissipation. A pedagogical way of deriving this non-dimensional number is as follows (e.g., Toomre 1993):

Consider two plates separated by a distance *d* and maintained at a temperature $T_{\mathrm{top}}=T_0$ and $T_{\mathrm{bottom}}=T_0 + {\varDelta } T$. Suppose we displace a fluid parcel less dense than the surrounding medium that rises vertically at a speed *w* but must compete against viscous drag: $\delta \rho g = \nu d^2 w/ dz^2 \sim \nu w/d^2$, with *z* the vertical coordinate, *g* the gravity and $\nu$ the kinematic viscosity. We deduce the following expression for the vertical velocity: $w = \delta \rho g d^2 / \nu$. For an ideal gas, one can relate density fluctuations to temperature variations ${\varDelta } T$ via the thermal expansion coefficient $\alpha _t$, e.g., $\delta \rho = \alpha _t {\varDelta } T$, such that $w = \alpha _t {\varDelta } T g d^2 / \nu$. While the parcel rises and since it is hot, it radiates away its heat. So in order to retain its buoyancy its rising time *d* / *w* must be faster than its thermal diffusion time $d^2/\kappa$, where $\kappa$ is the thermal diffusivity, e.g.: $d/w < d^2/\kappa$. This implies that:

4 $$\begin{aligned} 1 < \frac{\alpha _t {\varDelta } T g d^3}{\nu \kappa } = Ra \end{aligned}$$

Hence the Rayleigh number *Ra* must be greater than one for convective instability to develop (in this back of the envelope derivation). In reality, more sophisticated linear stability analysis (Chandrasekhar 1961) shows that, for example, the critical Rayleigh number for convective onset in a Cartesian layer heated from below depends on boundary conditions, with the value varying from $Ra_{\mathrm{crit}}=658$ for stress free boundary conditions to $Ra_{\mathrm{crit}}= 1708$ for no-slip boundary conditions. In stars the Rayleigh number is of order $10^{18}$, far in excess of the critical values determined for the simple plane-layer problem for any possible boundary conditions, so stellar convective motions are highly supercritical. The presence of rotation or intense magnetic field tends to modify the critical Rayleigh number (each individually making it larger). Chandrasekhar, however, demonstrated that the joint action of rotation and magnetism can yield surprising behavior, with the critical *Ra* for convection actually smaller in some cases in the presence of both rotation and magnetism.

While the simple prescriptions of convection discussed above have their merits for describing the one-dimensional, quasi-static structure of stars over secular time, such treatments of nonlinear convection lack several important physical properties. These include turbulent spatial and temporal energy distribution, velocity correlations, non-locality, and multi-scale convection to mention only a few. Fully characterizing these properties is essential for a modern understanding of the (magneto)-hydrodynamics of stars, and modern numerical simulations are one tool to do so, as we will show in Sect. 6. We also refer the reader to the following reviews, which cover different aspects of the problem in greater depth: see Living Reviews by Nordlund et al. (2009); Rieutord and Rincon (2010), the *Scholarpedia* article of Brun and Miesch (2008) and the recent nonlinear study of Featherstone and Hindman (2016a).

We now turn to discuss briefly the effect of rotation on stellar dynamics. This is a vast topic and we refer the reader to the following textbooks and articles for technical details: Pedlosky (1982), Zahn (1992), and Tassoul (2000). Rotation is ubiquitous in astrophysical bodies and plays a central role in shaping the secular evolution of stars as we discuss in Sects. 3.2 and 5.6. A first intuition on the influence of rotation on a stratified fluid system can be gained by observing Earth’s or Jupiter’s atmosphere: we notice the presence of cyclones, gyres, zonal jets, meridional cells of various intensity and orientation. We can deduce from this rich dynamics that rotation influences fluid flows, their turbulence and the transport of angular momentum within the system under study and can even trigger instabilities.

In a rotating frame, extra terms appear in the equation for conservation of momentum. Standard derivations (e.g., Pedlosky 1982) show that the acceleration of a fluid parcel in an inertial frame (denoted I) is related to that in a rotating frame (*R*, rotating with respect to the rotation vector ${\varvec{{\Omega }}}$) is

5 $$\begin{aligned} \left( \frac{d\mathbf{u}_I}{dt}\right) _I = \left( \frac{d\mathbf{u}_R}{dt}\right) _R+2{{\varvec{\Omega }}}\times \mathbf{u}_R+{{\varvec{\Omega }}}\times ({\varvec{\Omega }}\times \mathbf{r})+\frac{d{{\varvec{\Omega }}}}{dt}\times \mathbf{r}. \end{aligned}$$

So moving between the two frames *I* and *R* yields three extra terms: Coriolis acceleration $2{\varvec{\Omega }}\times \mathbf{u}_R$, centrifugal acceleration ${\varvec{\Omega }}\times ({\varvec{\Omega }}\times \mathbf{r})$ and an acceleration due to the variation of the rotation rate. This last term is relevant for stars on long secular time scales (see Sect. 5.6). For large rotation rates, centrifugal effects change the shape of the celestial body, making it flatter/prolate (e.g., its poles are closer to the center than equatorial regions). These effects are important when the star’s rotation reaches values comparable to the breakup velocity, at which point the star loses its internal coherence. We refer to the work of Espinosa Lara and Rieutord (2013, and references therein) for more details.

By contrast, the Coriolis force acts both on comparatively short^2^ dynamical time scales and makes its effect felt at much lower rotation rates than the centrifugal force. Below, we highlight two particularly important results that emerge as a consequence: namely, the Taylor–Proudman theorem and geostrophic balance.

**Fig. 20:**
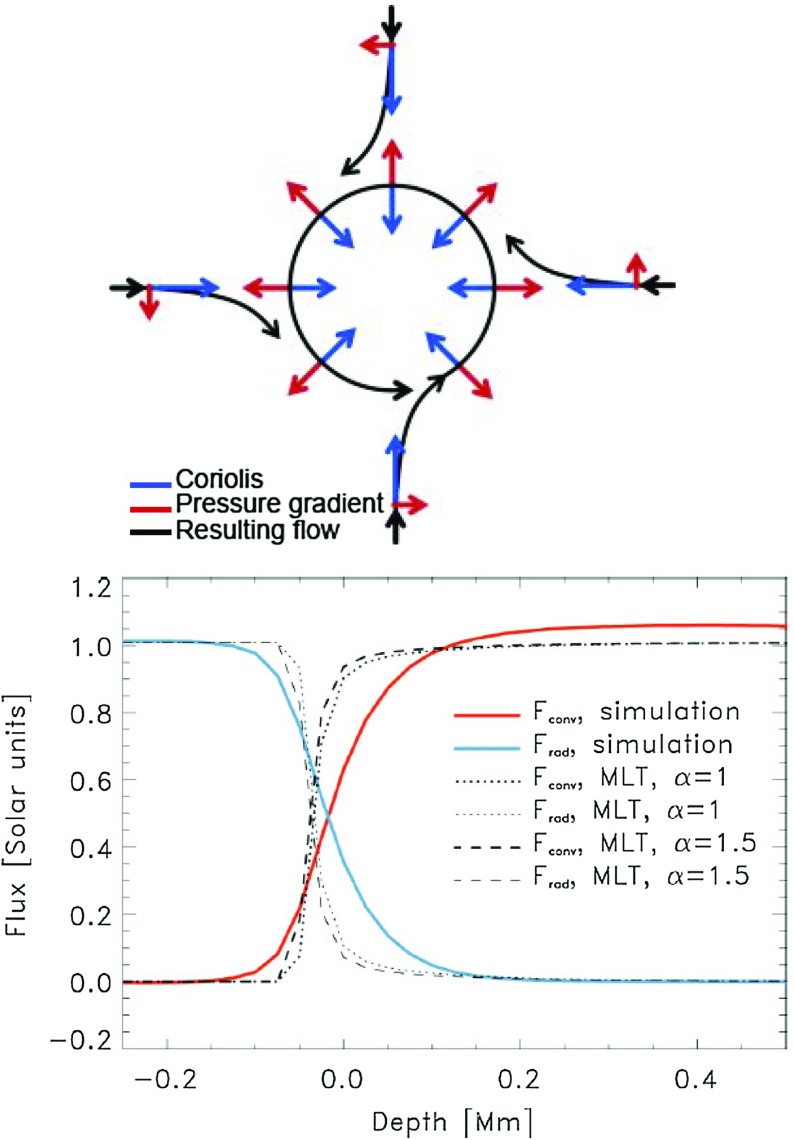
*Top* geostrophic balance and the influence of Coriolis pseudo force on fluid parcel motion. *Bottom* numerical simulation of the upper surface layer of the Sun compared to mixing length profiles using various $\lambda _p$ values. Image reproduced by permission from Abbett et al. (1997), copyright by AAS

Fluid parcels in a non-rotating system naturally tend to move from high pressure to low pressure areas, due to the action of the pressure gradient force. In a rotating system, as soon as fluid parcels begin to move they are acted upon by the Coriolis pseudo-force, which tends to deflect trajectories to the right in the northern hemisphere (or to the left in the southern). The limiting case where the pressure gradient force and the Coriolis force are equal and opposite is known as *geostrophic balance*.^3^ In this situation, the flow must ultimately be along isobars (rather than from high to low-pressure regions): otherwise there would be some component of the Coriolis force unbalanced by the pressure gradient (which is perpendicular to the isobars). As seen in Fig. 20 geostrophic balance is the reason why in the northern hemisphere, flow around a low-pressure system is counterclockwise, while that around a high-pressure system is clockwise; in the southern hemisphere the opposite situation prevails. Real systems are usually not in purely geostrophic states, with turbulence, inertia and thermal effect contributing to the global balance, as we will see in Sect. 6.

A related constraint can be derived by taking the curl of the Navier–Stokes equation to construct an equation for the vorticity ${\varvec{\omega }}$: in general this is modified by advection, by vortex stretching terms, by compressibility, and so forth, as laid out in detail in, e.g., Strugarek et al. (2011). In the useful limiting case of a stationary state ($\frac{\partial {\varvec{\omega }}}{\partial t} = 0$), within a nearly-adiabatic convection zone where both geostrophic balance and hydrostatic balance hold, the azimuthal component of the vorticity equation simplifies considerably to yield

6 $$\begin{aligned} \frac{\partial \langle v_{\phi }\rangle }{\partial z}=\frac{g}{2 {\varOmega }_0 r c_p}\frac{\partial \langle S\rangle }{\partial \theta }. \end{aligned}$$

Here $v_{\phi }$ is the longitudinal component of the velocity field $\mathbf{v}$ (with $\langle \rangle$ denoting temporal and azimuthal averages), the derivative $\displaystyle \frac{\partial }{\partial z}=\cos \theta \frac{\partial }{\partial r}-\frac{\sin \theta }{r}\frac{\partial }{\partial \theta }$ and other symbols take their usual meaning. This is a version of the so-called *thermal wind equation*; note that by assuming geostrophic and hydrostatic balance, we have assumed the influence of Reynolds stresses, magnetic fields, viscosity, etc, are all negligible (and in writing the equation in this form, we have also assumed that thermodynamic fluctuations away from an adiabatic background are small). If the flow is barotropic and density and pressure gradient are aligned, the right hand side of this equation is zero, and we recover the so-called *Taylor-Proudman constraint* that the flow become invariant along the axis parallel to rotation:: $\partial \langle v_{\phi } \rangle /{\partial z}=0$. Solid-body or cylindrical differential rotation profiles, for example, satisfy this constraint. (A corollary is that if the differential rotation profile is cylindrical, then the latitudinal entropy gradients must be negligible.) By contrast, if this term is non-zero then the flow is termed baroclinic, and a thermal wind contribution proportional to the entropy latitudinal variation is present. The nature of the internal thermal wind further changes in non-adiabatically stratified system where a term proportional to the entropy radial stratification emerges; see again Strugarek et al. (2011).

A further consequence of rotation, not immediately evident in the above analysis, is its potential effect on the properties of convective heat transport in the bulk of the domain. Some broad aspects are reviewed in Miesch and Toomre (2009), for example, so we mention only a few basic topics not covered there. In particular, we note that there has lately been renewed interest in the fundamental problem of determining the superadiabatic excess in rotating convection: though generically we expect this excess to be “small” (even a very small excess would correspond, in most models, to convection sufficient to carry the energy flux), in some cases even a small deviation from adiabaticity may have interesting consequences. Barker et al. (2014), for example, derive a version of rotating mixing-length theory that (in terms of its quantitative predictions) is functionally identical to that of Stevenson (1979); they argue that the temperature gradient in unstratified convection at mid-layer should scale as the rotation rate to the four-fifths power—i.e., more rapidly rotating systems require a stronger temperature gradient. They present numerical simulations (of rotating, Boussinesq convection in Cartesian domains) that bolster this view. Aurnou et al. (2015) summarize a variety of theoretical and laboratory work on the related topic of rapidly rotating convection in Earth and planetary cores; see also King et al. (2012), Stellmach et al. (2014), and Cheng et al. (2015) for analysis of the flow morphology and heat transport in various regimes. Separately, Julien et al. (2012) have conducted simulations of asymptotically reduced equations applicable to the rapidly rotating Rayleigh–Bénard problem, and find that while heat transport in the weakly-rotating limit is essentially set by the properties of the boundary layers, transport in the rapidly rotating regime is set by properties in the bulk. (Interestingly, the asymptotic theory of Julien et al. (2012) and the heuristic analysis of Barker et al. (2014), though derived in very different ways, appear to imply the same scaling for the bulk temperature gradient in some regimes; see discussion in Barker et al. (2014).)

In the discussions that follow, it will frequently be convenient to employ a series of nondimensional numbers that quantify the relative magnitudes of various terms in the Navier–Stokes equation. We have already mentioned the Reynolds number (which compares inertial terms $\mathbf{u} \cdot \nabla \mathbf{u}$ with viscous terms $\nu \nabla ^2 \mathbf{u}$), the Rayleigh number (equation 4 above, measuring buoyancy driving relative to dissipation), and the Rossby number (comparing inertial terms to Coriolis forces). Table 1 summarises these and a few others that are often employed in discussion of convection. Note that in a few cases different formulations of these numbers are used in different contexts: for example, the “classical” definition of the Rayleigh number quoted above (involving a temperature contrast ${\varDelta } T$) is most appropriate for classical Rayleigh–Bénard convection; in a stratified convection zone, not all of this temperature contrast is available to drive convection, so criteria involving the entropy change across the layer (${\varDelta } S$) are more relevant. Various local versions of these (involving for example the gradient of *S* at a point) are also in wide use. Similar comments apply to the Nusselt number, which generally is defined to be the ratio of total heat flux to that carried in the absence of convection; the version we quote here is again most appropriate for convection in a medium with constant thermal diffusivity $\kappa$ (and total flux *F*). Our summary in the Table is thus by no means exhaustive.

**Table 1:** A summary of nondimensional numbers commonly quoted in modeling of stellar fluid dynamics

Parameter	Definition	Meaning
Prandtl (*Pr*)	$\frac{\nu }{\kappa }$	Viscous/thermal diffusivities
magnetic Prandtl (Pm)	$\frac{\nu }{\eta }$	Viscous/magnetic diffusivities
Reynolds (*Re*)	$\frac{u L}{\nu }$	Inertia/viscous
Rossby (*Ro*)	$\frac{u}{2 {\varOmega } L}$	Inertia/Coriolis
Rayleigh (*Ra*)	$\frac{\alpha _t {\varDelta } T g d^3}{\nu \kappa }$	Buoyancy/dissipative
“Flux-based” *Ra*	$\frac{g F L^4}{c_p \rho T \nu \kappa ^2}$	“ ”
Ekman (*Ek*)	$\frac{\nu }{2 {\varOmega } L^2}$	Viscous/Coriolis
Taylor (*Ta*)	$\frac{4 {\varOmega }^2 L^4}{\nu ^2}$	Coriolis/viscous
Convective Rossby (Roc)	$\left( \frac{Ra}{Ta Pr}\right) ^{1/2}$	Buoyancy/Coriolis
Magnetic Reynolds (Rm)	$\frac{u L}{\eta }$	Induction/dissipation
Elsasser ($\varLambda$)	$\frac{B^2}{4 \pi \rho \eta {\varOmega }}$	Rm $\times$ Lorentz/Coriolis
Nusselt (*Nu*)	$\frac{F L}{\rho c_p \kappa {\varDelta } T}$	Total heat flux/conductive flux

See text for discussion and definitions

To summarize, we see that both the presence of turbulent convection and rotation in stars induces a rich array of interesting dynamical phenomena. These will be further assessed in Sect. 6 by means of 3-D non linear numerical simulations.

### Basics of dynamo theory

In a wide variety of astrophysical objects, magnetic fields are observed to evolve relatively quickly and/or to persist for relatively long times. By “quickly” or “long”, we refer to times that are very different from the characteristic decay time of magnetic fields in an imperfectly conducting medium, $\tau \sim L^2/\eta$ (where $\eta$ is the magnetic diffusivity, having units of length squared over time, and is related to the conductivity $\sigma$ by $\eta = c^2/(4 \pi \sigma )$).

A prominent example is the Sun, whose magnetism is evidently both persistent (we observe it today, after several Gyr of evolution) and variable (with the 22-year cycle of activity being the most prominent example); meanwhile the characteristic timescale for Ohmic decay would be many Gyr (e.g., Charbonneau 2010). Another example is the Earth, which likewise possesses a magnetic field that is too long-lived to be just remnant magnetization, and which is also observed to change polarity over irregular intervals of typically a few hundred thousand years (e.g., Laj and Channell 2007). These observations suggest the existence of a process that can dynamically build and maintain the magnetism (e.g., by converting kinetic energy to magnetic): a magnetic dynamo.

The literature on magnetic dynamos is vast; we note in particular the monograph by Moffatt (1978), and more recent reviews by Jones (2008), Ossendrijver (2003), and Brandenburg and Subramanian (2005), as examining the subject in more depth than we will attempt here. The recent review of Roberts and King (2013), though focused specifically on the Earth’s dynamo, also provides a good survey of many results from basic theory and simulation. We note in this subsection only a few of the most fundamental results of dynamo theory: why and when we might expect a dynamo to exist at all (and what equations describe its action), and the circumstances under which a dynamo is impossible. We also give a brief introduction to “mean field theory”, a method for solving analytically for the behavior of the large-scale field (usually as a function of the statistical properties of the velocity field) under certain assumptions, and comment briefly on when those assumptions are likely to apply. In the next section, we will move on to applying these ideas to solar and stellar magnetism specifically.

#### Dynamos in principle: equations, limits, and energetics

At its most basic, dynamo action in stars relies on the presence of an electrically conducting fluid (or plasma, or gas; we will use the terms interchangeably here). It is the currents associated with motion in that fluid that ultimately drive the dynamo. To see how this is possible in principle, recall that the induction equation of MHD (derived from Maxwell’s equations in the non-relativistic limit) is

7 $$\begin{aligned} \frac{\partial \mathbf{B}}{\partial t} = {\varvec{\nabla }}\times (\mathbf{v} \times \mathbf{B}) - {\varvec{\nabla }}\times (\eta {\varvec{\nabla }}\times \mathbf{B}) = {\varvec{\nabla }}\times (\mathbf{v} \times \mathbf{B}) + \eta \nabla ^2 \mathbf{B}, \end{aligned}$$

where the second equality holds if the diffusivity $\eta$ is independent of position. A derivation can be found in many textbooks and reviews; see, e.g., Kulsrud (2005), or Jones (2008). Expressed in this form, it relies essentially on Faraday’s law of induction, ${\varvec{\nabla }}\times \mathbf{E} = - \frac{\partial \mathbf{B}}{\partial t}$, on the relation between current density and magnetic field, $\frac{c}{4 \pi } {\varvec{\nabla }}\times \mathbf{B} = \mathbf{j}$ for non-relativistic material, and on the existence of some form of Ohm’s law in a moving medium, $\mathbf{j} = \sigma [ \mathbf{E} + \mathbf{v} \times \mathbf{B}]$.

#### When do we expect a dynamo?

Clearly if there is no motion at all ($\mathbf{v} = 0$), the field must decay away on a characteristic timescale $\tau _{\eta } = L^2 / \eta$. In the opposite limit of no diffusion (i.e., a perfectly conducting fluid), we have the “ideal MHD” limit. In this limit, it can be shown that the magnetic flux through any closed loop (i.e., the surface integral of **B** over that loop) remains constant as the loop moves around, a result known as *Alfvén’s theorem.* A practical consequence is that in this regime, magnetic field lines are “frozen in” to the fluid: they go where the plasma goes, they are compressed where the plasma is compressed and diluted where the plasma expands, and so forth. The problem of assessing field growth in this limit is substantially the same as that of assessing the trajectories of particles in a given flow field. Many relevant results are presented in the book by Childress and Gilbert (1995). A reasonable qualitative summary is that if the flow is sufficiently complex (as indicated, for example, by positive Lyapunov exponents; e.g., Manneville (2010)), then we may expect the energy in the magnetic field to grow (Tobias and Cattaneo 2008).

More generally, we might ask under what circumstances field growth is possible for finite values of the conductivity. It would be easy, but alas too simplistic, to suppose that as the conductivity gets high enough (i.e., $\eta$ gets small enough) the “induction” term ${\varvec{\nabla }}\times (\mathbf{v} \times \mathbf{B})$ must win out over the diffusive term $\eta \nabla ^2 \mathbf{B}$. The problem is that as the diffusivity is made smaller, the field may possess structure on finer scales, so the $\nabla ^2 B$ operator (which we might suppose scales like $B/l_d^2$, with $l_d$ some characteristic dissipative length) can get arbitrary large. Finding out which term “wins” is then a problem of some mathematical subtlety; determining the growth rate (rather than just whether it is positive) is harder still. Still, we might reasonably expect that dynamo action will be possible at some sufficiently high values of the magnetic Reynolds number $Rm = \frac{U L}{\eta }$ (with *U* and *L* characteristic velocities and lengths), and impossible at lower values. We will see in Sect. 6 that this qualitative expectation is born out by many numerical examples; here, we note only a few brief limits. The rate of change of the magnetic energy within a sphere of radius *a* matching to a decaying potential outside the sphere, $\partial E_m/\partial t$ (with $E_m = B^2/(8 \pi )$), is

8 $$\begin{aligned} \frac{\partial E_m}{\partial t} \le \left( \frac{ a u_{\mathrm{max}}}{\pi } - \eta \right) \int {| {\varvec{\nabla }}\times \mathbf{B} |^2 dV} \end{aligned}$$

with $u_{\mathrm{max}}$ the maximum value of $\mathbf{u}$ within the sphere (see, e.g., Jones 2008). Thus a working dynamo requires $Rm = \frac{a u_{\mathrm{max}}}{\eta } \ge \pi$. In practice, the minimum *Rm* needed for dynamo growth may be considerably higher! The critical *Rm* for growth is in general likely to be a function of other parameters; for example, it is clear that it is a function of the magnetic Prandtl number ($Pm = \nu /\eta$) in some regimes, and in particular that it can increase appreciably below $Pm \approx 1$ (see, e.g., Boldyrev and Cattaneo 2004; Schekochihin et al. 2005; Iskakov et al. 2007). Stars are generally characterized by very large values of *Rm*: as a rule of thumb, for non-degenerate conductivity by electrons, the magnetic diffusivity (in cm$^2$ s$^{-1}$) is of order $10^{4} T^{-1/2}$, so that for example a roughly person-sized object moving at walking speed ($u \sim 1$ m s$^{-1}$ and $L \sim 1$ m has $Rm \approx 1$ at a temperature of $10^6$ K. Hence, because the predominant scales of motion in stellar interiors are much larger than this, it is likely that in most cases *Rm* is (on some scales) greater than any plausible threshold value necessary for dynamo action.

Another well-known result was proven by Cowling (1933), who showed that no *axisymmetric* magnetic field vanishing at infinity can be maintained by dynamo action (now often called “Cowling’s theorem”). Note that this result does *not* imply that dynamos with axisymmetric $\mathbf {u}$ are impossible; see, e.g., discussion in Jones (2008), and specifically the example flows of Dudley and James (1989) or Ponomarenko (1973), for illustrations of dynamos with non-axisymmetric $\mathbf{B}$ (thus evading Cowling’s theorem) but axisymmetric $\mathbf{u}$. It is also possible to prove that no purely *toroidal* flow (i.e., one that can be written in the form $\mathbf{u} = {\varvec{\nabla }}\times T \hat{r}$) can act as a dynamo. This provides an important constraint on dynamo action in stars (or planets, for that matter): some poloidal flow (e.g., provided by convection or by some other instability) is essential to the dynamo’s operation.

#### Estimates of field strength

These constraints on whether there is a dynamo at all, or on its growth rate, can be addressed using *linear, kinematic* theory: i.e., by consideration of the induction equation alone. In general we would also like to know something about the fields that are ultimately built by dynamo action. How strong do they become, for example? What is their resulting spatial structure? In most cases we do not yet have definitive answers to these questions, but we can provide a few qualitative estimates. First, note that these estimates are beyond the purview of linear theory, which does not take into account the Lorentz force feedback of the (growing) magnetism on the flow (through the Lorentz force, $\mathbf{j} \times \mathbf{B}$). This feedback plays a crucial role in setting the equilibrated field strength in most instances, and we might also expect it to have a considerable impact on the field morphology (i.e., the spatial structure of the field might well be different in the kinematic phase and in the nonlinearly equilibrated one). So we must turn to some version of nonlinear theory.

The field strengths ultimately achieved are not easy to estimate, and typically we must resort to either simple heuristic models (as we will do here), to semi-analytical theories of how the field affects the flow (discussed in the next section), or to numerical simulations (as in Sect. 6). Quite generally, the field will equilibrate when its growth by induction is balanced by losses; usually the main “loss” mechanism is Ohmic dissipation, but in some contexts it could instead represent losses of magnetism through the open boundaries of the dynamo region (with an associated non-negligible Poynting flux). The problem is that both the induction and the dissipation depend sensitively on the properties of the flow field: the rate of creation of magnetic energy is related to $\mathbf{u} \cdot \mathbf{j} \times \mathbf{B}$, for example, but $\mathbf{u}$ and $\mathbf{B}$ are often nearly parallel, so that seemingly small changes in the flow can have a great influence on the dynamo. Although it is therefore not possible to predict a priori what balance of induction and dissipation will be achieved, it is instructive to consider a few important limiting cases.

**Fig. 21:**
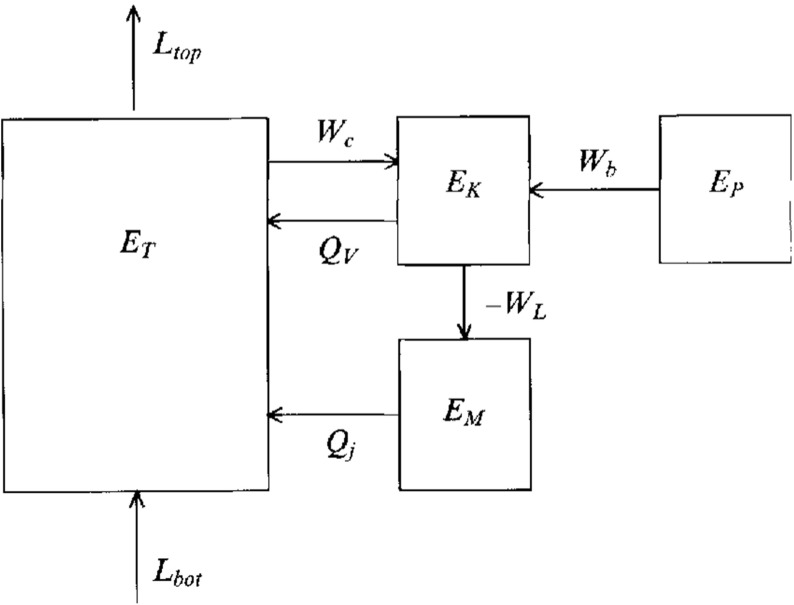
Cartoon of energy flows between reservoirs in stellar convective dynamos. $E_T$, $E_K$, $E_M$, $E_P$, $W_L$, $W_c$, $W_b$, $Q_V$ and $Q_j$ stands respectively for: thermal energy, kinetic energy, magnetic energy potential energy, Lorentz work, pressure work, buoyancy work, viscous dissipation and Ohmic loss. $L_{bot}$ and $L_{top}$ represent the star’s luminosity. Image reproduced by permission from Brandenburg et al. (1996), copyright by CUP

In astrophysics, one commonly employed estimate is that the magnetic energy density will equilibrate when it reaches “equipartition” with the kinetic energy density of some process that is playing a role in building the field. (Be warned that some authors instead use the term “equipartition” to refer to equality between the magnetic pressure and the gas pressure.) One motivation for this is that in a *closed* system with no dissipation, the sum of magnetic and kinetic energies is identically conserved—so a firm bound on the *final* magnetic energy is the *initial* kinetic energy. Note, though, that even in this very idealized system, the ratio of final magnetic energy to final KE could in principle exceed unity by an arbitrary amount. Furthermore, most astrophysical dynamos, far from being closed systems, are strongly *driven*: vast reservoirs of potential and internal energy in various forms are present, and these may at any time re-establish a velocity field whose energy was “stolen” by the magnetism. See Fig. 21 for an illustration of the possible flow of energy between these reservoirs, and (Starr and Gilman 1966; Hewitt et al. 1975; Brandenburg et al. 1996; Rempel 2005, 2006) for discussion of related issues. (Note, also, that there are varying conventions for defining the buoyancy work, pressure work, etc, as referenced here; see discussions in the Living Review of Nordlund et al. (2009) and in Viallet et al. (2013), for example.)

We might also estimate equilibrated magnetic energies by turning to the momentum equation, and asking what balances of forces are plausible in any given situation. For example, if the Lorentz force $\mathbf{j} \times \mathbf{B}$ is approximated simply by $jB \sim B^{2}/L,$ with *L* some characteristic spatial scale, and balances inertial terms, which are taken to scale as $\rho v \cdot \nabla v \sim \rho v^2 / L$, we recover $B^2 \sim \rho v^2$ (see, e.g., Roberts 2009)—i.e., equipartition with the kinetic energy density of the flow. But we could instead have imagined that the Lorentz forces would equilibrate with the Coriolis force, and arrived at a different estimate of the field strength. In the geophysical literature, for example, this is often expressed as an argument that the Elsasser number $\sim \sigma B^2/(\rho {\varOmega }) \approx 1$; more generally, it is often argued that in the geodynamo a balance between magnetic, buoyancy, and Coriolis forces (called “MAC balance”, with the “A” standing for “Archimedean”) may be reached, with inertial and viscous stresses playing essentially no role. Such estimates can suggest magnetic energies of order the kinetic energy divided by the Rossby number ($Ro \sim U/({\varOmega } R)$), implying that in rapidly rotating systems (which have *Ro* small) the magnetic energy might greatly exceed the convective kinetic energy. How these different limiting cases might connect to each other as the rotation rate of a dynamo is smoothly varied is not yet clear, but there are many conceptual models (e.g., Christensen et al. 2009; Davidson 2013; Oruba and Dormy 2014; Brun et al. 2015b). At more extreme field strengths, Browning et al. (2016) have argued that a limit on the field strengths achievable by any steady-state dynamo may arise from the joint action of magnetic buoyancy instability and Ohmic dissipation: strong, fibril fields would dissipate too much energy, while strong larger-scale fields would rise too quickly to be regenerated by convection. (They considered the case of M-dwarfs specifically, but similar considerations would apply to other objects.)

With these caveats firmly in place: in many contexts equipartition with the turbulent flow field is probably a reasonable estimate of the overall level of magnetic energy. In numerical simulations of the dynamo process, something of this order is a very common result (see Sect. 6). It also agrees (to order of magnitude) with what are sometimes called “flux-based” estimates of the field strength (Christensen and Aubert 2006; Christensen et al. 2009): to see this, note that the flux convection must carry, *F*, is related to the convective velocity in mixing-length theory (e.g., Hansen and Kawaler 1994) (or on dimensional grounds) by $F \sim \rho v_c^3$, so that equipartition ($B^2 \sim 4 \pi \rho v^2$) implies $B^2 \propto \rho ^{1/3} F^{2/3}$. These estimates in turn seem to agree tolerably well with observations of the surface magnetic field strength in objects ranging from planets to rapidly rotating stars (Christensen et al. 2009), though very recently there have been suggestions that some brown dwarfs may have significantly stronger fields than these relations would predict (Kao et al. 2015).

Assessing the spatial *structure* of the equilibrated field is harder, and we do not yet have a general heuristic theory. To assess the structure of the field in a somewhat quantitative way, we turn in the next section to the subject of *mean field dynamo theory*.

#### Mean field theory

Although constraints on the overall level of magnetic energy are useful, in many situations we would also like to know something about the magnetic field’s spatial structure and temporal variability. Not all spatial scales of the magnetism have equal influence on a star or planet’s evolution: the stellar wind torque, for example, is influenced mainly by the largest-scale global fields (i.e., the dipolar or quadupolar components, see Sect. 5.6), rather than by the turbulent, small-scale field (though the latter influences the level of chromospheric heating and mass loss). Mean-field theory is essentially a way of trying to solve for the evolution of these large-scale fields by *parameterizing* the combined effects of the smaller-scale flows and fields. The statistical properties of the small-scale flow, rather than its detailed character, together with bulk parameters like the rotation rate, then figure into an evolution equation for the large-scale field, which can be rigorously solved in certain limits. We will not describe the subject in great detail; it is summarized more completely in the reviews by Brandenburg and Subramanian (2005) and (with application to the solar dynamo specifically) Charbonneau (2010). We give only a brief outline of the method, its main strengths and limitations, and some key results. In the next section we outline some of the main conclusions drawn from such models, as applied to stars and planets.

We begin by splitting the magnetic and velocity fields into a mean part and fluctuations around that mean:

$$\begin{aligned} \mathbf{B}= & {} \langle \mathbf{B}\rangle + \mathbf{b}' \quad \text{ with } \quad \langle \mathbf{b'}\rangle =0,\\ \mathbf{v}= & {} \langle \mathbf{V}\rangle + \mathbf{v}'\quad \text{ with } \quad \langle \mathbf{v'}\rangle =0 \end{aligned}$$

where $\langle \, \rangle$ represents a suitable average: i.e., an ensemble average or an appropriate spatial or temporal average (see, e.g., Moffatt 1978, for discussion). By averaging the induction equation on an intermediate scale $l \ll \lambda \ll L$ we obtain an evolution equation for the mean field, which we will presume can be associated with large scales:

9 $$\begin{aligned} \frac{\partial \langle \mathbf{B}\rangle }{\partial t}={\varvec{\nabla }}\times (\langle \mathbf{V}\rangle \times \langle \mathbf{B}\rangle + \langle {{\varvec{\mathcal {E}}}}\rangle -\eta {\varvec{\nabla }}\times \langle \mathbf{B}\rangle ) \end{aligned}$$

where $\langle {{\varvec{\mathcal {E}}}}\rangle = \langle \mathbf{v}' {{\times }}\mathbf{b}'\rangle$ represents the mean electromotive force from small-scale turbulence. So far we have not made any simplifications (apart from assuming the existence of a sensible averaging procedure), but we also haven’t really made our lives any easier: since $\langle {{\varvec{\mathcal {E}}}}\rangle$ involves the fluctuating field (which is also unknown), we still can’t solve for $\langle \mathbf{B}\rangle$. To go further, we require a closure relation (or other model) linking the small scale field to the large scale field. In general, this relation doesn’t have to exist: e.g., if there is small-scale dynamo action, then the fluctuating field $\mathbf{b}'$ can grow even if $\langle \mathbf{B}\rangle$ is zero. But if such small-scale dynamo action is absent, or dominated by the fluctuations associated with the small-scale velocity acting on the large-scale field, then the induction equation for $\mathbf{b}'$ is linear in $\langle \mathbf{B}\rangle$, and we may anticipate that a Taylor expansion for $\langle {{\varvec{\mathcal {E}}}}\rangle$ around the mean field $\langle \mathbf{B}\rangle$ will converge:

10 $$\begin{aligned} {{{\varvec{\mathcal {E}}}}} = \mathbf {a} \cdot \langle \mathbf{B}\rangle + \mathbf {b} \cdot \nabla \langle \mathbf{B}\rangle +\cdots \end{aligned}$$

where $\mathbf {a}$ and $\mathbf {b}$ are tensors of rank two and three, respectively. Dividing these (and the derivative tensor $\nabla \langle \mathbf{B}\rangle$) into symmetric and antisymmetric parts, one can rewrite this as (see, e.g., Warnecke et al. 2016)

11 $$\begin{aligned} {{{\varvec{\mathcal {E}}}}} = {\varvec{\alpha }}\cdot \langle \mathbf{B}\rangle + {\varvec{\gamma }}\times \langle \mathbf{B}\rangle - {\varvec{\beta }}\cdot ({\varvec{\nabla }}\times \langle \mathbf{B}\rangle ) - {\varvec{\delta }}\times ({\varvec{\nabla }}\times \langle \mathbf{B}\rangle ) - {\varvec{\kappa }}\cdot ({\varvec{\nabla }}\langle \mathbf{B}\rangle )^{s} +\cdots \end{aligned}$$

where ${\varvec{\alpha }}$ and ${\varvec{\gamma }}$ are the symmetric and anti-symmetric parts of the tensor $\mathbf {a}$, ${\varvec{\beta }}$ and ${\varvec{\delta }}$ are the symmetric and anti-symmetric parts of $\mathbf {b}$, and ${\varvec{\kappa }}$ is a rank-3 tensor. Often, this is further rewritten in a coordinate-specific form, with the ${\varvec{\alpha }}$ and ${\varvec{\beta }}$ terms assumed dominant:

12 $$\begin{aligned} \mathcal{E}_i=\alpha _{ij} {<}B_j{>} + \beta _{ijk} \frac{\partial {<}B_j{>}}{\partial x_k} + \cdots , \end{aligned}$$

where $\delta _{ij}$ is the Kronecker symbol and $\epsilon _{ijk}$ the Levi-Cita symbol. This results in the induction equation for the mean field:

13 $$\begin{aligned} \frac{\partial \langle \mathbf{B}\rangle }{\partial t}={\varvec{\nabla }}\times (\langle \mathbf{V}\rangle \times \langle \mathbf{B}\rangle + \alpha \langle \mathbf{B}\rangle -(\eta +\beta ){\varvec{\nabla }}\times \langle \mathbf{B}\rangle ) \end{aligned}$$

where we have considered essentially the simplest possible case for the coefficients $\alpha _{ij} = \alpha \delta _{ij}$ and $\beta _{ijk} = \beta \epsilon _{ijk}$ (see, e.g., Moffatt 1978; Ossendrijver 2003). The first term in the above equation represents the transport and stretching of the field from large scale motions (whether meridional circulation or differential rotation, with the latter referred to as the ${\varOmega }$ effect); the second is called the $\alpha$ effect (whether linked to either cyclonic turbulence or tilted active regions); the last is diffusion with an effective diffusivity $\beta$ (which we might expect can greatly exceed the Ohmic diffusivity $\eta$). We also note that the ${\varvec{\gamma }}$ appearing in (some of) the equations here can be interpreted physically as “pumping” of the magnetic field by an effective velocity field.

Under the so called first order smooth approximation (FOSA; or other closure schemes), one can then solve for $\alpha$ in terms of the statistical properties of the turbulence; in the simplest case with FOSA, for illustration, the result is that

14 $$\begin{aligned} \alpha \approx - \frac{1}{3} \tau _{\mathrm{corr}} \langle \mathbf{v} \cdot {\varvec{\omega }}\rangle \end{aligned}$$

where ${\varvec{\omega }}= {\varvec{\nabla }}\times \mathbf{u}$ is the vorticity and $\tau _{\mathrm{corr}}$ is the correlation time of the turbulence. In general, other terms arise, and a frequently-employed result is that $\alpha$ has both the above, “kinetic” piece as well as a magnetic contribution, $-\langle \mathbf{j}' \cdot \mathbf{b}' \rangle /\rho$ with $\mathbf{j}'$ the fluctuating current density. See, e.g., (Pouquet et al. 1976). If the kinetic helicity is known, we can now express the electromotive force as a function of the large-scale *B*, and hence (substituting into the induction equation for the large-scale field above) obtain an evolution equation that can at last be solved for the large-scale field $\langle \mathbf{B}\rangle$.

### Applications to solar and stellar dynamos

#### Overview of mean field models

This mean field approach to dynamo theory has been used for decades to study the magnetism of stars, planets, and galaxies. A good overview for the solar case is provided in the review by Charbonneau (2010). In general, the solutions to the mean-field equations are classified according to which effects dominate the production of poloidal and toroidal field. An “$\alpha ^2$” dynamo would be one where the “$\alpha$-effect” (i.e., the $\alpha$ term in the above equations, arising from the cumulative effects of smaller-scale turbulence) generates both poloidal field from toroidal field, and vice versa. In an “$\alpha -{\varOmega }$” dynamo, the generation of poloidal field (from toroidal) is still dominated by the $\alpha$-effect, but the generation of toroidal field is mainly due to the ${\varOmega }$-effect (i.e., the linear winding of fieldlines by differential rotation). An “$\alpha ^2-{\varOmega }$” dynamo has both $\alpha$ and ${\varOmega }$ effects operating in comparable measure to produce the toroidal fields.

These models have been remarkably successful, in the specific sense that if one adopts qualitatively reasonable models for the $\alpha$-effect, ${\varOmega }$-effect, etc, solving the mean-field equations can yield patterns of magnetic field emergence and behavior that closely resemble what occurs on the Sun (Roberts 1972; Roberts and Stix 1972; Stix 1976). Until the mid-1980s, the most widely favored mean-field solutions for the solar dynamo problem involved a distributed $\alpha -{\varOmega }$ dynamo operating amid the solar convection zone. These models obey a relation often known as the Parker-Yoshimura sign rule, which relates the direction of propagation of dynamo “waves” to the properties of $\alpha$ and to the differential rotation: $\mathbf{s} = \alpha {\varvec{\nabla }}{\varOmega } \times \mathbf{e_{\phi }}$, with $\mathbf{s}$ the direction of propagation (Parker 1955a; Yoshimura 1975; Stix 1976). In such models, the product of $\alpha$ and $\partial {\varOmega }/\partial r$ must therefore be negative in the northern hemisphere in order to obtain an equatorward butterfly diagram. As noted above, the sign of $\alpha$ is related to that of the kinetic helicity, $H_k = \mathbf{v} \cdot ({\varvec{\nabla }}\times \mathbf{v})$, and $H_k$ in turn is typically negative in the northern hemisphere owing to the properties of the rotating convection (but see discussion in, e.g., Duarte et al. 2015, who point out circumstances where the opposite sign may prevail). One problem arose when helioseismology revealed that the differential rotation profile was nearly conical at mid-latitudes (e.g., Thompson et al. 1996), i.e. $\partial {\varOmega }/\partial r \simeq 0$ there, rather than cylindrical. Another came with the growing realization that strong magnetic fields should ultimately reduce the efficiency of the field generation by helical convection, the phenomenon now usually called $\alpha$-quenching (e.g., Ossendrijver 2003) and discussed briefly below. Partly motivated by these difficulties, and also by the fact that magnetic buoyancy instability might lead to the loss of fields amid the convection zone more rapidly than they are regenerated (see, e.g., Parker 1955a, 1975; Hughes and Proctor 1988; Fan 2009; Browning et al. 2016, for discussions), this distributed dynamo was largely supplanted by models in which the sites of poloidal and toroidal field generation were not distributed uniformly throughout the convection zone but accomplished in distinct regions. In particular, Spiegel and Weiss (1980) and Golub et al. (1981) suggested that strong toroidal fields might be concentrated into a boundary layer at the base of the convection zone; Parker (1993), motivated partly by the helioseismic inference of a tachocline of shear at the base of the solar convection zone, elaborated a model in which the sites of poloidal and toroidal field generation were segregated, in what is now called the *interface dynamo.* Later, Charbonneau and MacGregor (1997) developed a mean-field model incorporating all the elements that today form part of standard interface dynamo theory, including a solar-like differential rotation, a tachocline, and field generation occuring in spatially distinct regions (with toroidal field built mainly in the tachocline and poloidal field built in the convection zone). This model, suitably tuned, successfully reproduced several observed aspects of the solar cycle, including the phase relation between poloidal and toroidal fields, the 22-year cycle, and the butterfly diagram, though it is worth noting that it is quite difficult in practice to find classical $\alpha$-${\varOmega }$ solutions that agree with all these constraints. Many later papers have built on this basic idea, with various refinements.

#### Babcock–Leighton effects and flux transport

In our discussion so far, helical convection has been presumed to be the main physical mechanism behind the production of poloidal field from toroidal field with rising convective eddies stretching the field and systematically twisting it, as proposed by Parker (1955b). But other effects can build poloidal field from toroidal as well. As recognized by Babcock (1961) and explored by Leighton (1964, 1969), the decay of tilted active regions at the Solar surface is also a source term for poloidal field, and indeed there is now strong evidence that the reversal of the surface poloidal field is triggered by this decay (see, e.g., Wang et al. 1989a; Cameron and Schüssler 2015). For many years, this mechanism was largely ignored in comparison to the $\alpha$-effect associated with convective eddies; see, e.g., discussion in Charbonneau (2010). In the last three decades, however, there has been a resurgence in models incorporating this source term (now called the Babcock–Leighton effect) in some fashion Wang et al. (1991), Choudhuri et al. (1995), Durney (1995), (see also review by Charbonneau (2010) for a more recent description of such models). These have been partly driven by increasing realization of the strong links between emerging active regions and the reversal of the global field (see, e.g., Babcock 1961; DeRosa et al. 2012). They have also provided a way of circumvening some of the difficulties that “classical” MFT models (based on helical $\alpha$-effect) faced in matching the observational data. Almost simultaneously models also began to include the effects of meridional circulation: (e.g., Wang et al. 1991; Choudhuri et al. 1995; Dikpati and Charbonneau 1999, and references therein) computed Babcock–Leighton models with a single-celled meridional flow and a solar-like differential rotation. They showed that such a “Babcock–Leighton flux transport” (BLFT) dynamo model could successfully reproduce a number of key solar global magnetic properties. The meridional circulation plays a pivotal role in the behavior of this model and many others like it, by transporting poloidal field from regions near the surface to the bottom of the convection zone, where it is converted into toroidal field by shear (Jouve and Brun 2007b).

#### Open issues and overview

Despite decades of effort, there is still no universally accepted “model”, much less a truly predictive theory, for the operation of the global solar dynamo. At the most fundamental level, there is still uncertainty over the extent to which mean-field dynamo theory is applicable to the Sun at all, given that the former (in the incarnations usually adopted) formally assumes conditions that manifestly do not occur in the Solar interior (see, e.g., discussion in Cattaneo and Hughes 2009). It may be that the interaction between small-scale and large-scale fields, and the shear, cannot readily be described within the mean-field framework; see, for example, the notion of “essentially nonlinear” dynamos explored in Tobias et al. (2001). Much attention is now focused on the interaction between small-scale growing modes of the dynamo and large-scale ones, and the mediation of these by shear (see, e.g., Tobias and Cattaneo 2013). We will return to discussion of some of these issues in Sect. 6. Even within the specific framework of mean-field dynamo theory, however, central open questions involve the relative importance of the tachocline, meridional circulations, induction by helical convection, turbulent diffusivity, and magnetic pumping—which the astute reader will have noticed are most of the elements involved in the dynamo in the first place. Within the (even more restrictive) class of BLFT dynamos, for example, there is substantial active debate about the relative roles of advection (by the meridional flow) and “diffusion” (e.g., by turbulent convection), with different classes of models known as advection-dominated (e.g., Dikpati et al. 2006) or diffusion-dominated (e.g., Choudhuri et al. 2007). See also Yeates et al. (2008) and Muñoz-Jaramillo et al. (2011). It seems safe to say that at present, hybrid mean-field models incorporating all evident sources of poloidal field give the best agreement with observations (see, e.g., Dikpati et al. 2004). In light of the central role that meridional flows play in many of these models (e.g., Jouve and Brun 2007b; Guerrero and de Gouveia Dal Pino 2008; Brun and Rempel 2009; Nandy et al. 2011; Hazra et al. 2014), measurements of that flow are particularly crucial, and are an area of great current interest (see, e.g., Hathaway 2012; Zhao et al. 2013; Schad et al. 2013; Dikpati et al. 2014; Hung et al. 2015; Sun et al. 2015) and discussion in (Brun et al. 2015a). We will return briefly to these measurements in Sect. 7.

#### Application to stars other than the Sun

Many of the mean-field concepts developed for the solar dynamo have naturally been applied to the more general stellar dynamo problem as well. As in the solar case, there is little consensus regarding which effects are likely to be most important for which stars, so we note only a few broad points. First, the existence of a “rotation-activity correlation”, as highlighted in Sect. 4, clearly provides an important constraint on dynamo theory. So, too, does the evident link between surface magnetism (as traced by, e.g., coronal and chromospheric activity) and the presence of surface convection, likewise discussed in Sect. 4. Together, these suggest that for stars across a broad swath of the H-R diagram, both convection and rotation (possibly including internal differential rotation) play major roles in building the field, whether directly or indirectly. By directly or indirectly, we mean that in the language of MFT, convection could (for example) act as the predominant source of an $\alpha$-effect, or could instead just provide the turbulent diffusion (and sustain meridional circulations and differential rotation) that are essential for operation of Babcock–Leighton flux-transport dynamos. Major open questions involve how the field “saturates” at any given rotation rate, and how other parameters like the cycle period or the presence of “grand minima” likewise vary with stellar mass and rotation rate.

The strong, observed correlation between rotation rate and magnetic activity (Noyes et al. 1984a, and as reviewed above) provides one example of a prominent constraint on such models. The observational suggestion that cycle periods and rotation are linked (see, e.g., Saar and Brandenburg 1999, 2002; Böhm-Vitense 2007; do Nascimento et al. 2014) is another. Clearly the surface magnetism is sensitive to both convection and rotation at some level, but how these all conspire to yield the observed trends is not yet clear. For recent efforts, see for example Blackman and Owen (2016) and Blackman and Thomas (2015).

As an example of how these observations—coupled with theory and simulations—may help discriminate between models, note for example that in classic “flux transport” models, weaker meridional circulations imply a longer cycle period (e.g., Jouve et al. (2010)). Meanwhile numerical simulations (Ballot et al. 2007; Browning 2008; Augustson et al. 2012; Brun et al. 2017) suggest that meridional circulations tend to weaken as the model rotates faster. Together, these would suggest that more rapid rotation should imply *longer* cycle periods (if the flux transport dynamo were dominant) unless for instance the advection path is modified to be shorter by considering multi-cellular flows. So depending on the profiles of the various physical ingredients used in this class of mean field dynamo models different trends can be obtained that can be directly confronted to observational ones.

Finally, we note that it is of course possible—and indeed likely—that different classes of dynamo models may more closely approximate the behaviour that arises in stars of different masses and ages. For example, even if the Sun today is broadly describable in MFT terms as an $\alpha -{\varOmega }$ dynamo, one might suspect that stars with less differential rotation (e.g., because it is strongly “quenched” by magnetism) might be more akin to $\alpha ^2$ dynamos; similarly, even if today the Babcock–Leighton mechanism plays a crucial role in the Solar dynamo, it is by no means clear that this would always be so (for example, even at times in the Sun’s own past when few spots emerged, or in other stars). More generally, even if some stars are well-described within the confines of MFT, others may not be; for example (as discussed more thoroughly in Sect. 6.4) an early theoretical expectation (e.g., Durney et al. 1993) was that fully convective low-mass stars (which do not possess a shear layer akin to the Solar tachocline) might harbor only comparatively small-scale “turbulent” dynamos, failing to build large-scale ordered fields of any sort. (By contrast, Chabrier and Küker (2006), argued that such stars could effectively act as $\alpha ^2$ mean field dynamos; meanwhile the simulations discussed in Sect. 6.4 generally suggest that large-scale field generation is indeed likely in some regimes, whether describable in the language of MFT or not.) Finally, we note that few of the “beyond MFT”-type dynamos recently proposed in the Solar context—see, e.g., Tobias et al. (2011), Cattaneo and Tobias (2014), Tobias and Cattaneo (2013), and discussion in Sect. 6.1—have yet been seriously applied in the context of stars other than the Sun.

#### Summary of models and their observational attributes

In this section, we have discussed a variety of different dynamo mechanisms, including some that fit within the bounds of mean-field theory and others that do not. Ideally, we would be able to list observational features that clearly distinguish these models from one another. Key testable elements might include the rotational dependence of different models, their propensity to exhibit magnetic cycles (and the period of such cycles), and the strength and morphology of the magnetism. Unfortunately, the situation is not so clear cut. Many broad classes of models make similar predictions about the nature of the observable magnetism, or can be adjusted to do so; meanwhile some of the other conceptual models discussed above have not yet been developed to the point where they can really be compared to observations. Part of the problem is that the properties of the observable field may ultimately encode more information about the way in which the field *saturates* nonlinearly than the way it which it is *built* kinematically.

In the specific context of mean-field theory, the saturation mechanism is somewhat distinct from the dynamo “mode”. One can construct consistent $\alpha$-${\varOmega }$ or $\alpha ^2$ dynamos with different “quenching” scenarios for either the $\alpha$ or ${\varOmega }$ effects, so that it is not possible to state emphatically that, for example, an $\alpha ^2$ dynamo yields a stronger or weaker field than an $\alpha -{\varOmega }$ one. Often in mean field models either the differential rotation is assumed to be modified by Lorentz force feedbacks from large-scale fields (the “Malkus–Proctor effect”, after Malkus and Proctor 1975), or the small-scale motions producing the $\alpha$-effect are taken to respond to the growing magnetism, whether according to the “catastrophic quenching” formulae given above or in accord with more complex dynamical quenching expressions (e.g., Blackman and Brandenburg 2002). The character of the solutions depends to some extent on the nature of this quenching. Still, it is useful to summarize some of the broad features present in the models discussed so far. To that end, we present in Table 2 a slightly tongue-in-cheek analysis of some potentially observable features of different dynamo models—namely their rotational dependence, their temporal variability, and their spatial structure—together with a summary of the same elements for the Sun.

**Table 2:** A light-hearted summary of some observable features of selected dynamo models and objects

Type	Rotation dep?	Variability	Morphology
$\alpha ^2$	y	Typically steady	$\equiv$ large-scale (ls)
$\alpha -{\varOmega }$	y	Typically cyclic	ls
Interface $\alpha -{\varOmega }$	y	Typically cyclic	ls
“Essentially nonlin”	?	?	ls or ss
“Turbulent”	n	Chaotic	Small-scale
“Suppressed”	Varies	Varies	ls
MHD sims (global)	y (varies)	Varies	Both
The Sun	y	Cyclic	Both

Each line corresponds to a class of dynamo (or, in the final case, an observed object), and some notation about whether it exhibits rotational dependence, the nature of its *typical* temporal variability, and its spatial morphology (specifically “large-scale” or “small-scale”, denoted LS and SS respectively). See text for discussion and details

The entries in this table require some explanation. We have chosen seven representative models: the $\alpha$-${\varOmega }$, $\alpha ^2$, and “interface” $\alpha$-${\varOmega }$ listings refer to standard mean-field models as described above in their most “typical” form; many variants of these exist. For a thorough summary, we again refer to Brandenburg and Subramanian (2005) and Charbonneau (2010). We also list “essentially nonlinear” models, e.g., as described by Tobias et al. (2011), and the “turbulent small-scale dynamo”, as described, e.g., by Durney et al. (1993) or (in the Solar context) Cattaneo (1999). By the former, we really mean any model in which the nonlinear effect of strong fields is crucial in subsequent field evolution; the latter refers to the chaotic stretching of field lines described above. We have also listed “suppressed” dynamos, by which we refer to any model in which both large-scale and small-scale dynamo action are present, but in which the rapid growth of small-scale fields is suppressed by some effect, whether shear, nonlinearity, or diffusion (as discussed in Cattaneo and Tobias 2014; Pongkitiwanichakul et al. 2016). Finally, for comparison we also summarize what is found in 3D global MHD simulations (as described in Sect. 6) and in the Sun and other solar-like stars. For each entry, we have indicated whether the model typically exhibits clear rotational dependence, we give some indication of the temporal variability that is usually found (steady, cyclical, or chaotic), and we give an indication of whether the magnetism is structured on “large” (i.e., system-sized) scales or small ones. We have not listed the strength of the nonlinearly equilibrated field, partly because this depends (for the mean field models) primarily on the “quenching” adopted for the $\alpha$ and ${\varOmega }$ effects. As one consequence, the “interface” dynamo listings are here just identical to the standard $\alpha$-${\varOmega }$ ones: the former can be regarded as a subset of the latter, in which the quenching mechanism is physically well-motivated. (Namely, catastrophic quenching is avoided in these models by having the strongest toroidal field built in a layer that is spatially distinct from the region where the $\alpha$ effect operates.)

Clearly, in a summary of this form some important details are lost, so the next few paragraphs provide some clarifications about the spatial structure, rotational dependence, and temporal variability of these models. First, consider the spatial structure of the fields. By definition, all the mean field models listed produce “large-scale” (mean) fields. The classic “small-scale turbulent dynamo”, also by definition, produces energy on the scale of the eddies building the field, with only a small contribution expected at larger scales (essentially the random sum over uncorrelated eddies). The global-scale simulations and the Sun both exhibit power on a broad range of scales; in simulations, as described in Sect. 6, the fraction of the field on “large” scales typically depends on both physical parameters (like rotation rate) and on numerical ones (like the overall resistivity or resolution of the simulation). The spatial structure of the “suppressed” and “essentially nonlinear” models could likewise vary, but in practice the focus of these models has largely been as a way to allow large-scale field growth to “win” over the action of small-scale turbulent dynamo action.

Next, consider the listed rotational dependence. Most of the models are sensitive to rotation at some level - either because they depend on the kinetic helicity of the convection, which itself varies with the rotation rate, or because they rely on some level of rotational shear. We can illustrate how this works explicitly in the classic $\alpha$-${\varOmega }$ dynamo, following (e.g.) Durney and Latour (1978), Noyes et al. (1984b), Baliunas et al. (1996), Montesinos et al. (2001): if $\alpha \approx - \tau _c \mathbf{u} \cdot \nabla \times \mathbf{u}$, with $\tau _c = L/u$ a large-scale convective overturning time, and if the helicity itself is proportional to $u {\varOmega }$, we have $\alpha \propto {\varOmega } L$. We can define an effective Reynolds number measuring the $\alpha$ effect, $N_{\alpha } = \alpha R / \eta$, involving a turbulent diffusivity $\eta = L^2 / \tau _c$. Meanwhile production of toroidal field via the ${\varOmega }$-effect may similarly be quantified by $N_{\omega } = {\varDelta } {\varOmega } R^3 / \eta$, with ${\varDelta } {\varOmega } \sim {\varOmega } /L_{\mathrm{shear}}$ the angular velocity gradient across a layer $L_{\mathrm{shear}}$. The behavior of the $\alpha$-${\varOmega }$ solutions is then characterized by

15 $$\begin{aligned} D = N_{\alpha } N_{{\varOmega }} \propto ({\varOmega } \tau _c)^2 (R/L)^4, \end{aligned}$$

(assuming $L_{\mathrm{shear}} = L$) which is called the “dynamo number”. Growing solutions to the kinematic dynamo problem exist when *D* exceeds a certain critical value; further, the cycle period in the kinematic solutions is $P_{\mathrm{cyc}} \propto D^{-1/2} \propto {\varOmega }^{-1}$. Even in this simple model, the nonlinear cycle period may be different than this kinematic estimate (Noyes et al. 1984b). Estimates of the properties of the nonlinear state depend on which of the different “quenching” prescriptions is used; see, e.g., discussion in Moss and Brooke (2000). In the case of the “suppressed” and “essentially nonlinear” models, solutions with or without rotational dependence are probably possible, but for example in the models of Pongkitiwanichakul et al. (2016), rotation again enters the problem both through the shear and through the helicity of the flow. In the classic “turbulent” dynamo, by contrast, the field is taken to be more or less uninfluenced by rotation; this is physically well motivated in the case of near-surface Solar convection, for which the convective turnover times are so short that Coriolis forces probably are unimportant, but is less likely to be a reasonable model when applied to deep convection with much longer turnover times.

Finally, we have listed some measure of the temporal variability that “typically” occurs in these models. The simplest $\alpha ^2$ dynamo models admit steady solutions, and typical $\alpha$-${\varOmega }$ models admit cyclical ones, but we caution that exceptions to both these rules exist. For example, $\alpha ^2$ models with spatially variable $\alpha$-effects can also produce cycles in some circumstances (see discussion in Rüdiger et al. 2003). The prototypical “turbulent” dynamo exhibits irregular polarity fluctuations; the “essentially nonlinear” and “suppressed” models could in principle exhibit a variety of behavior, but again practical interest has largely focused on cases that exhibit cyclical dynamo waves. Global simulations exhibit an enormous variety of behavior, with cyclical, steady, or chaotic solutions all possible (see Sect. 6). The Sun, of course, exhibits a regular magnetic cycle with large-scale patterns of field emergence and propagation—though it also possesses small-scale magnetism that contains enormously more energy than that in the large-scale field. It also possesses longer-term behavior, with modulations of cycle amplitude and “grand minima” both well-documented; see, e.g., Usoskin (2013) and Hathaway (2015). Such very low activity states have been explained in the litterature mainly by two different approaches, either via stochasticity of the dynamo sources (such as the $\alpha$-effect) (Ossendrijver and Hoyng 1996; Ossendrijver 2003) or by deterministic non-linear dynamo models. In the later type, small values of the magnetic Prandtl number *Pm* yield more time variable solutions with grand minima period where the amplitude of the magnetic field is significantly depressed (see discussion Tobias 1997; Moss and Brooke 2000; Covas et al. 2005; Bushby 2006, and in Sect. 6.3).

### Fossil fields

Not all observed stellar magnetic fields owe their existence to contemporary dynamo action. As noted above, the characteristic Ohmic decay time for large-scale magnetism in a star is typically of order Gyr or more. A relic field, produced for instance as part of the star formation process or by dynamo action on the pre-main-sequence phase, might therefore persist throughout the entire main-sequence lifetime of all but the least massive stars. In the Sun, and in other stars with surface convection zones, it is reasonable to assume that the characteristic decay time for the field would be very much less than this (large-scale) Ohmic decay time: provided the field does not entirely quench the convection, the convective eddies will tend to structure the field on progressively smaller scales, ultimately leading to decay on some comparatively rapid “eddy diffusion” time. (We might crudely estimate this time as $\tau \sim L^2 /\eta _{\mathrm{ed}}$, with *L* the initial spatial scale of the field and $\eta _{\mathrm{ed}}$ the product of typical velocities and lengthscales for the eddies; the point is that this decay time depends on the details of the velocity field, not on the microscopic conductivity as such.) But not all stars have surface convection, and we may suppose that observable fields in such stars might be “fossils” of an earlier process. The magnetic Ap/Bp stars, discussed briefly in Sect. 4.5, are usually thought to be of this type; similarly, the magnetic fields observed in white dwarfs and neutron stars, though perhaps produced at earlier stages of the star’s evolution by dynamo action of some sort, are probably not today maintained by any dynamo process and are thus also in some sense “fossilized”. Several recent reviews have discussed aspects of such fields; see, in particular, the recent review by Braithwaite and Spruit (2015) for an overview of field evolution in non-convective stars. We note here only a few brief points regarding the strengths such fields might reach, their stability over time, and some aspects of their appearance at the stellar surface.

#### How strong should fossil fields be?

In the fossil field scenario, the field observed today is the relic of processes that occurred in the star’s past: that is, its strength may principally encode information about dynamics that occurred some time ago (where “some time” must still be less than the overall Ohmic diffusion time) rather than anything occurring today. The field strengths that could be reached in principle are quite high: for example, simulations of the collapse of magnetized molecular clouds, adopting reasonable initial values of the mass-to-flux ratio (see, e.g., Mestel 1999) can yield strengths of 10–100 kG in the proto-stellar core (e.g., Bate et al. 2014). How such strong fields would interact with convection occurring on the pre-main-sequence is unclear, but for example (Moss 2003) argued that at least some of the field implanted by the star formation process would survive to the main sequence. There is no obvious reason why the fields inherited from this process would depend significantly on rotation rate (though an indirect dependence from the influence of Hayashi-phase convection may be possible), in accord with the observation that the magnetism of Ap/Bp stars does not vary strongly with rotation rate (Sect. 4). At the opposite extreme, it is not entirely clear what would set the minimum possible strength of fossil fields. Braithwaite and Cantiello (2013), for example, argued that the very weak fields (of less than 1 G strength) observed in some massive stars might be “failed fossils”, remnants that are still dynamically relaxing. What sets the field strength in any specific star—and in turn determines whether it exhibits the whole host of “Ap” phenomena—is not clear, and could in principle depend on “initial conditions” (i.e., the properties of the protostar, the cloud out of which it formed, and so forth) or on subsequent evolution. For examples of the (rather complex) interactions possible, see Mestel et al. (1988), Moss et al. (1990), or Wei and Goodman (2015). Most recently, Gaurat et al. (2015) have investigated the dichotomy between the strong “Ap-type” fields and the sub-gauss magnetism observed in other cases, and argued (similarly to Aurière et al. 2007; Lignières et al. 2014) that the lower bound of observed field strengths in stably stratified stars might arise essentially from a stability condition: strong enough fields are stable and persist, while weaker ones decay quickly. In all cases, a central role is played by whether the field configurations are stable for intervals comparable to the main-sequence lifetime of the star, so we turn to that topic next.

#### Evolution and stability of fields

**Fig. 22:**
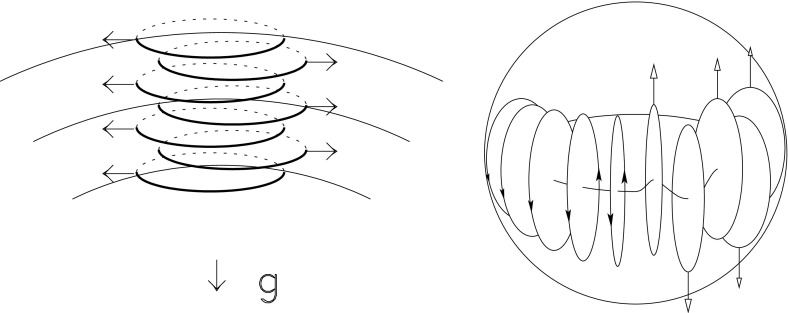
Cartoons illustrating the instability of purely toroidal and poloidal fields. In the purely toroidal case (*left*), the field may be thought of as a series of magnetic rings that can “slip”; in the purely poloidal case the behavior resembles that of bar magnets that can rotate. Images reproduced by permission from Braithwaite (2007) and Spruit (1999), copyright by ESO

A central challenge to the idea of fossil fields in stars, as recognized decades ago, was to find a field configuration that is stable for extended intervals: trivial field configurations (purely toroidal, or purely poloidal) were known analytically to be unstable on an Alfvén time (see Tayler 1973; Markey and Tayler 1973; Wright 1974; Flowers and Ruderman 1977; Acheson 1979; Pitts and Tayler 1985). A recent review is provided by Braithwaite and Spruit (2015). (To persist, the field must also be in dynamical equilibrium—i.e., all the forces acting on it must balance—but this is generically less difficult to arrange. The search for stability/instability presumes that some initial equilibrium exists, and examines the response to small perturbations around that equilibrium.) Some insight into why purely toroidal and poloidal fields are unstable may be afforded by Fig. 22, adapted from (Spruit 1999) and (Braithwaite 2007). As noted by Braithwaite, in the case of a purely toroidal field (left), we may think of the field as a series of magnetic “rings”, each exerting pressure on the other; this pressure can cause some of the rings to “slip”, in the same way that disks in your backbone can slip, or a stack of dishes can too-easily clatter to the ground. Meanwhile a star with a purely poloidal field (right) may be thought of as consisting of two bar magnets that are free to rotate; if the magnets start out aligned, they will tend to rotate so that they are anti-aligned, and the stellar magnetic field in the non-rotating case turns out to behave similarly. In both cases the result is instability. Rotation tends to stabilize the system somewhat, but analytical and numerical work suggest this just modifies the growth rates of the instability (but does not stop it). The solution, as explored analytically in Prendergast (1956) and Wright (1973), and first explicitly demonstrated by numerical simulations in the work of Braithwaite and Spruit (2004), is a mixed toroidal–poloidal configuration. In the simulations by Braithwaite and collaborators, a variety of initial field configurations quickly evolve towards this mixed configuration; the simplest example is that of a single flux tube, threaded by a poloidal field. See, e.g., Braithwaite and Nordlund (2006), Braithwaite (2009), Duez and Mathis (2010), Akgün et al. (2013), and the discussion in Sect. 6.5.

If differential rotation is initially present, then in principle the energy in this flow might be tapped, to amplify an initially weak magnetic field. The idea, as outlined in Spruit (2002), is that the MHD instabilities outlined above provide a way to “close the dynamo loop” even in a stably stratified region: differential rotation stretches poloidal field into toroidal, but these toroidal fields are ultimately unstable to the pinch-type (Tayler) instability, which in turn gives rise to poloidal fields. (Magnetic buoyancy instabilities should also occur at sufficiently strong field strengths, of course, but in the presence of strong stratification (Spruit 1999, 2002) argued the Tayler instability would arise first.) These might then be stretched anew, allowing the dynamo to progress, ultimately at the expense of the kinetic energy in the differential rotation, unless this is actively maintained by some other agent. Various technical complications, largely beyond the scope of this review, may render this mechanism somewhat more complex than it at first seems. One issue, as noted for example in Zahn et al. (2007), is that the linear winding of non-axisymmetric fields by differential rotation can only produce a non-axisymmetric field; if one wishes to create a “mean” field specifically (i.e., an axisymmetric poloidal or toroidal field), there must still be a net emf $\langle {\varvec{\nabla }}\times (\mathbf{v'} \times \mathbf{b'}) \rangle$, where the $\mathbf{b'}$, $\mathbf{v'}$ are now associated not with (say) convection but with the fluctuating (non-axisymmetric) fields induced by the Tayler instability. This axisymmetric component is often regarded in the dynamo literature as crucial; if it is not regenerated, then clearly the process has failed to act as a “mean field” dynamo. In general, this “mean field” version of the Tayler–Spruit dynamo will require extra ingredients—e.g., helicity—that may not always be present. Still, the prospect of dynamo action in such regions, whether on large or small scales, is extraordinarily enticing, and so the problem has been studied in considerable detail by subsequent authors; for some comparatively recent analytical analyses (with comparison to mean-field dynamo theory), see for example Rüdiger et al. (2016), Rüdiger et al. (2012). For the conceptually related problem of determining the mean emf arising from magnetic buoyancy instabilities, see for example Davies and Hughes (2011). Ultimately, to help elucidate the circumstances under which such dynamo action may occur, the character of the resulting fields, and their consequences (e.g., for angular momentum transport), many authors have turned to numerical simulations; we outline some results of these calculations in Sect. 6.5.

### Flux emergence and stellar spots

Sunspots have been observed for centuries but it is only during the modern era that a clear link between their darkness and the presence of strong magnetic fields inhibiting convective heat transport has been made. Historical records date back to the 17th century with Galileo’s first observations. Continuous efforts to collect and to create a coherent set of sunspot records have recently converged to a new time series (Hoyt and Schatten 1998; Usoskin 2013; Clette et al. 2014; Svalgaard and Schatten 2016) covering more than 4 centuries.

Currently it is believed that the origin of magnetic sunspots is due to the emergence of magnetic flux ropes created by turbulence and shear, either in the tachocline as often assumed (Parker 1955b, 1993) or in the near surface shear layer (Brandenburg 2005). An alternative to the rise of magnetic flux rope has been proposed by Stein and Nordlund (2012). They consider the rise of a uniform horizontal field through a convective layer and observe the formation of intense field concentration akin to a spot. It remains to be seen if such a scenario can lead to the formation of a penumbra around the magnetic spot and flow like the evershed effect (Rempel 2015).

Complex sunspot groups of mixed polarities associated to active regions are ideal locations for eruptive events such as flares or CMEs. Understanding flux emergence through the convective granular surface into the chromosphere and the associated sunspot magnetic topology and dynamics is thus key to better characterize solar activity. Helioseismology has recently been used to detect sunspots just before their emergence (Gizon and Birch 2005; Kosovichev 2009; Gizon et al. 2010; Birch et al. 2010; Ilonidis et al. 2011) and thanks to holography techniques, the far side of the Sun is being probed on a daily basis (Lindsey and Braun 2000; Braun and Lindsey 2001; González Hernández et al. 2013).

3-D Numerical simulations of the emergence of magnetic ropes in either local (Abbett et al. 2000; Fan et al. 2003; Archontis et al. 2004; Murray et al. 2006; Isobe et al. 2006; Martínez-Sykora et al. 2008; Hood et al. 2009; Toriumi and Yokoyama 2010; Archontis et al. 2013; Rempel and Cheung 2014; Takasao et al. 2015; Martínez-Sykora et al. 2015) or global settings (Jouve and Brun 2009; Jouve et al. 2013; Weber et al. 2011; Pinto and Brun 2013) have been performed by many groups to understand sun spot formation. In earlier studies the concept of thin magnetic flux tube was used (Spruit 1981; Spruit and Ballegooijen 1982; Spruit and Roberts 1983) and magnetic ropes were rising in a quiescent atmosphere. In the more recent numerical simulations, fully developed convective flows act on the magnetic structures, resulting in more complex evolution and spatial structuring of the emerging flux.

**Fig. 23:**
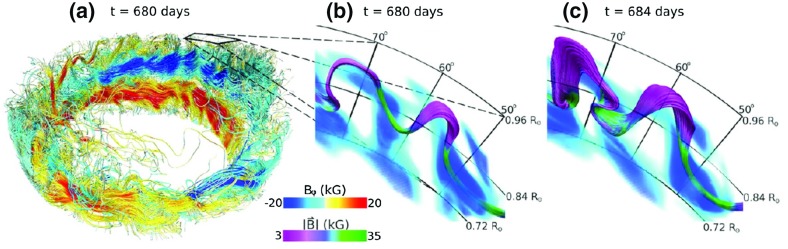
First self-consistent simulation of a convective dynamo generating simultaneously a large scale magnetic field and buoyant omega-loop like magnetic structures, thought to be at the origin of starspots (Nelson et al. 2011, 2014). Shown on the *left* is the longitudinal component of the magnetic field (with *red* denoting positive polarity) rendered with magnetic field lines that form a large scale magnetic wreaths around the equator. In the *middle* and *right panel*, zoom on the evolution of a buoyant magnetic omega-loop over 4 days (*colored* using the amplitude of the total magnetic field). We note the clear radial rise of the structure

In a series of papers, Nelson et al. (2011, 2014) have discussed the first convective dynamo that generates self-consistently rising magnetic loops from large scale magnetic wreaths. This is key as the idealized thin flux tube approximation and regular magnetic ropes that are often used in the numerical experiments cited just before are unlikely to exist in the Sun (Tobias et al. 2011). On the contrary, in self-consistently dynamo generated toroidal flux structures, the magnetic field is rather fibril and corrugated. We illustrate such instance in Fig. 23. A detailed analysis suggests that low magnetic diffusivity and low Rossby number are key ingredients for such a “spot-dynamo” to develop (Brun et al. 2015a).

We refer the interested reader to the Living Review by Fan (2009) for a thorough discussion of the physical processes associated to flux emergence and for a detailed accounting of the various theoretical and numerical studies done on this topic.

Sunspots can be used as prototypes of star spots as discussed in detail in Schrijver (2002). Solar surface flux transport or photometric models have been used to reproduce the solar magnetic flux and light modulations over the 11-year cycle and can be extended to other stars (e.g., Wang et al. 1989b; Schrijver 2001; Schrijver and DeRosa 2003; Krivova et al. 2003; Lanza et al. 2003; Dikpati 2011, and references therein). Indeed by analogy to the dimming of light that sunspots create as they pass on the surface of the Sun, light modulation in photometric observations of stars have been associated to starspots. Inspired by solar photometric models, surface spot modelling simulations have been developed to reproduce such light curve modulations due to rotation and surface star spots (Lanza et al. 2006; Mosser et al. 2009; Lanza 2016). It is an ill-posed problem. Many configurations of spot number, size or distribution can reproduced a given light curves, but Monte-Carlo techniques or Bayesian techniques can help to find the optimal solutions. Stellar photometric data with satellites such as COROT, *Kepler* and soon Tess and Plato have extensively used such spot modeling. These models do not generally seek to understand the physics (formation, structure and evolution) of star spots, in contrast to the 3-D MHD simulations discussed above. Still they can provide useful information on stellar spot distribution (size, number, location). For instance, coupled with the analysis of transiting planets that can occult starspots, they bring new constraints (Silva-Valio et al. 2010).

Around sunspots there are bright faculae that dominate the overall luminosity budget such that the Sun is actually brighter at cycle maxima (Foukal et al. 1991; Spruit 2000; Pap and Fox 2003; Domingo et al. 2009; Fröhlich 2012; Ermolli et al. 2012). Understanding how the dark sunspot/bright faculae ratio evolves as a function of stellar parameters (Chapman and McGuire 1977; Radick et al. 1998; Messina et al. 2003; Lockwood et al. 2007; Shapiro et al. 2014) is key when studying stellar activity and searching for exoplanets through transits or Radial Velocity methods (Moutou et al. 2005; Oshagh et al. 2013; Dumusque et al. 2014, and references therein). In some extreme cases they can actually cover most of the stellar surface. The blockage of the heat that it implies does not change the star’s structure except possibly for low mass fully convective stars (Mullan and MacDonald 2001; Chabrier et al. 2007).

**Fig. 24:**
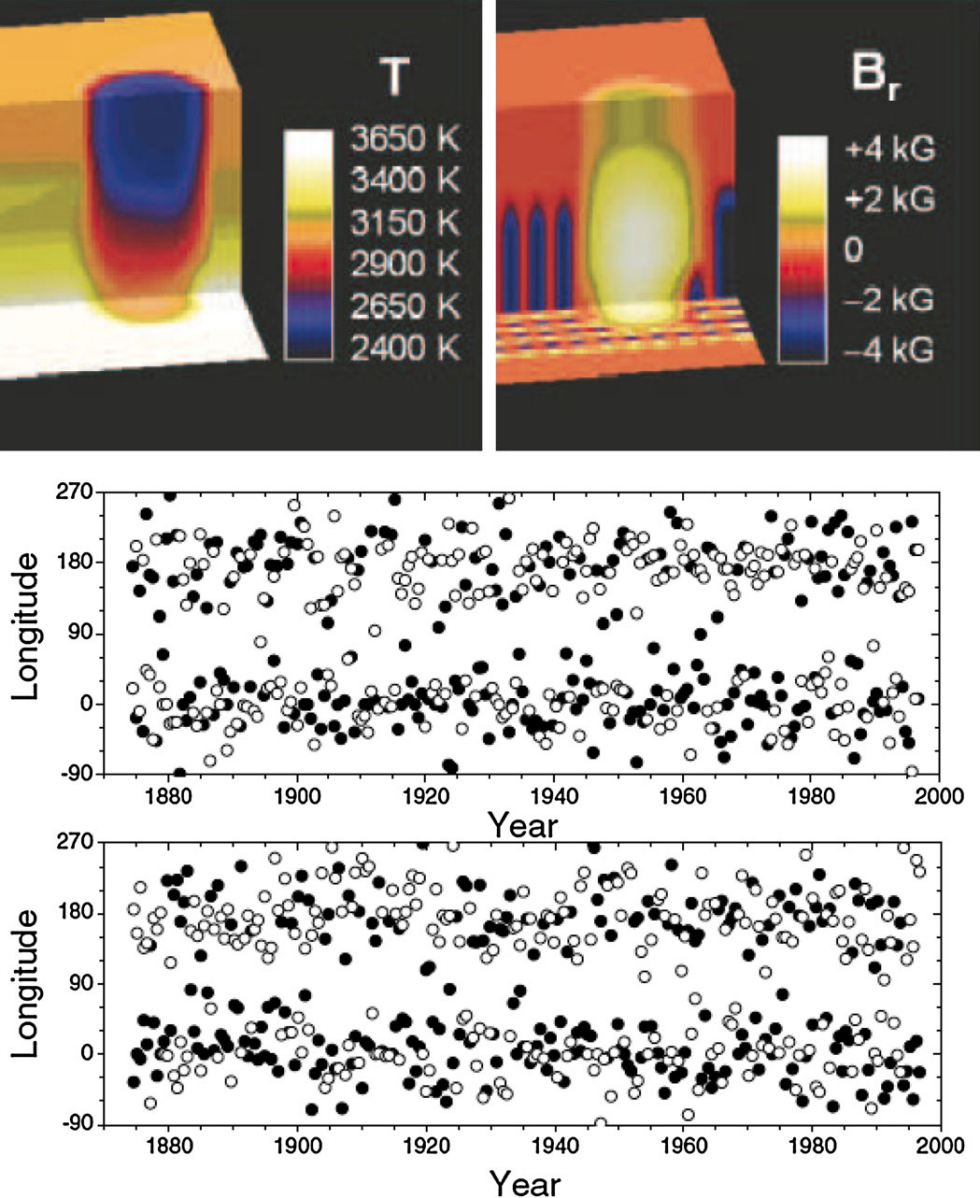
*Top* inversion of the 3-D structure of a starspot on AU Mic using spectropolarimetric technique at various line depth formation. A field of 3.5 kG (assumed to be radial) and and a temperature deficit of more than 1000 K are compatible with observations. Image reproduced by permission from Berdyugina (2011), copyright by ASP. *Bottom* longitudinal position of solar active regions over 130 year for the northern (*top*) and southern (*bottom*) hemispheres. *Filled* and *open circles* represent the dominant and secondary active longitudes respectively. We note in each hemisphere two bands roughly 180 degree of longitude apart, with altering intensity levels known as the flip-flop phenomenon. Image reproduced by permission from Berdyugina et al. (2006), copyright by ESO

Other important questions naturally arise: How intense and large can starspots be? Can they emerge at different latitudes, closer to the polar region than in solar case? Spectropolarimetric maps have recently brought partial answers to these questions. For instance young active stars (which spin rapidly) seem to harbor large polar spots (Strassmeier 2001; Schrijver and Title 2001). Strassmeier (1999) even reported, in the evolved K0III primary star of XX Tri, a giant star spot larger than the whole Sun and more than 10,000 times larger than the largest sunspot ever recorded. Hence all evidence indicates that starspots are ubiquitous in active stars and that they can be detected at all latitudes, with a tendency for fast rotators to harbor polar spots.

In order to understand how the latitude of emergence varies with stellar parameters, Işık et al. (2011) have coupled a flux emergence simulation to a mean field dynamo model to study solar-like star and the influence of rotation. They confirm that when the rotation of the star is fast, star spots emerge at high latitude, forming large polar caps. This is due in part to the cancellation of the horizontal component of the buoyancy force by the Coriolis force acting on the rising magnetic ribbon, resulting in a vertical rise, parallel to the rotation axis of the star (see for instance Schüssler et al. 1996; Jouve and Brun 2007a).

As with solar active regions, complex starspot systems can lead to intense flaring events and even possibly to superflares (Shibata and Magara 2011; Maehara et al. 2012). We refer to Berdyugina (2005), Strassmeier (2009) for recent overviews of starspots, their size, lifetime, ocurrence and even their radial structure by using various spectral lines to probe various heights within the stellar atmosphere. We show in Fig. 24 an example of an inversion of the vertical structure of a star spot (see Berdyugina 2011).

We also refer to the work of Berdyugina and Usoskin (2003) for an analysis of active longitudes (e.g., where sunspot/starspot emergence seems favored)) in solar and stellar cycles. For instance, a quick change of 180$^\circ$ in longitude of stellar activity has been observed. This must come about when both activity sites reach an equivalent level of intensity in the observed field. It has been named the flip-flop phenomenon. In the Sun, such flip-flop phenomenon, while weaker than in young stars, has a cycle period of about one third the 11 year cycle. The active longitude needs to be tracked by substracting off the drift due to the surface differential rotation. In Fig. 24 (bottom panels) we show a reconstruction over the last 130 years of active longitudes almost 180 degree apart. A non-axisymmetric dynamo mode, $m=1$ instability of toroidal magnetic field or stroboscopic effects, the latter being favored, may be at the origin of this phenomena (Moss 2004; Usoskin et al. 2007; Usoskin 2013).

### Magnetic effects on coronal activity and winds

As stars evolve on the main sequence a complex feedback loop operates between their level of magnetic activity and their rotation rate. Through magnetic wind braking solar-like stars spin down as they age old solar-like stars being on average slow rotators. Skumanich (1972) showed that the rotation rate of solar like stars follows the following law: ${\varOmega }_*(t) \propto t^{-1/2}$ (at least until reaching the solar age, where it may break down van Saders et al. (2016)).

Observations of open clusters further revealed that the large spread in stellar rotation rates observed in young systems is significantly reduced by the age of the Hyades (650 Myr) and by the age of the Sun, it has mostly vanished. This relation between the spin rate and a star’s age is known as *gyrochronology* (Barnes 2003, 2010). Since dynamo action in solar-like star is intimately linked to the rotation of the star (Moffatt 1978; Weiss 1994), as stars spin down they change their level of magnetic activity and their magnetic geometry (Gregory et al. 2012; Vidotto et al. 2014a; Folsom et al. 2016). The change of magnetic field amplitude and possibly geometry over the secular evolution of stars has been named *magnetochronology* in echo to the term used for rotational evolution. The change of field geometry in stars has a direct impact on their braking rate since it modifies the properties of their wind as well as the location of the Alfvén radius $r_A$, e.g., the radius beyond which the stellar wind speed exceed the local Alfvén speed $v_A=B/\sqrt{4\pi \rho }$. Indeed it can be shown that angular momentum loss is directly proportional to the square of the Alfvén radius $\dot{J}=\dot{M} r_A^2 {\varOmega }_*$, with $\dot{M}$ the stellar mass loss (see discussion in Schatzman 1962; Weber and Davis 1967; Matt and Pudritz 2008; Réville et al. 2015a).

We have discussed in the previous sections in detail how stellar dynamos operate and we will summarize the recent findings through nonlinear numerical simulations in the next sections. Here we wish to discuss briefly the current status of stellar wind models, in particular of solar-like stars for which the main driver is thermal pressure, and how magnetic field amplitude and geometry influence the corona and the torque applied by stellar winds.

Solar-like stars on the main sequence evolve on long time scales; the solar main sequence lifetime, for example, is of the order 10 Gyr. Computing such long temporal (secular) evolution with multi-D codes is still usually impossible or impractical. Instead, we must rely on 1-D stellar evolution models to describe the structure, chemical and rotational evolution of stars on secular time scales. Multi-D models can aid the study of specific phases of this evolution by describing, sometimes in great detail, dynamical nonlinear processes and (in the specific context of stellar winds) by providing scaling laws and torque formulations. By incorporating such laws into “toy” models, it may be possible to assess what physical mechanisms are essential to explain the evolution of the rotation of stars. For instance, MacGregor and Brenner (1991) have proposed a simple two-zones stellar angular momentum evolution model that has proven to be quite useful. In such framework, solar-type stars are divided into two spherical concentric domains: an outer turbulent convective envelope coupled to an inner stably stratified core. Both are allowed to rotate independently around a rotating axis aligned with the *z* axis of a 3-D (*x*, *y*, *z*) cartesian system. Both possess their own angular velocity ${\varOmega }$ and moment of inertia *I*. For both zones, structural evolution and magnetized winds can build a differential rotation in the radial direction. Their angular momentum is respectively $J_{\mathrm{env}}=I_{\mathrm{env}}{\varOmega }_{\mathrm{env}}$ and $J_{\mathrm{core}}=I_{\mathrm{core}}{\varOmega }_{\mathrm{core}}$. To evaluate the amount of angular momentum exchange ${\varDelta } J$ between the two zones needed to have uniform rotation ${\varOmega }_{*}$, we need to characterize the inital and final states of both zones. The initial state is straightforwardly written: $J_{\mathrm{init}}^e= J_{\mathrm{env}}$ and $J_{\mathrm{init}}^c= J_{\mathrm{core}}$. From total angular momentum conservation e.g., $J_{\mathrm{init}}^c + J_{\mathrm{init}}^e = J_{\mathrm{final}}^c + J_{\mathrm{final}}^e$, we deduce that the final state is: $J_{\mathrm{final}}^e= J_{\mathrm{env}} + {\varDelta } J = I_{\mathrm{env}} {\varOmega }_{*}$ and $J_{\mathrm{final}}^c= J_{\mathrm{core}} - {\varDelta } J = I_{\mathrm{core}} {\varOmega }_{*}$. Substitution yields:

16 $$\begin{aligned} {\varDelta } J = \frac{I_{\mathrm{env}}J_{\mathrm{core}}-I_{\mathrm{core}}J_{\mathrm{env}}}{I_{\mathrm{core}}+I_{\mathrm{env}}}. \end{aligned}$$

In the case where an external torque (for instance coming from a magnetized stellar wind) is applied to the stellar surface, the angular momentum evolution of the two-zones stellar model can be written as (MacGregor and Brenner 1991):

17 $$\begin{aligned} \frac{dJ_{\mathrm{core}}}{dt}= & {} - \frac{{\varDelta } J}{t_{\mathrm{c}}} \end{aligned}$$

18 $$\begin{aligned} \frac{dJ_{\mathrm{env}}}{dt}= & {} \frac{{\varDelta } J}{t_{\mathrm{c}}} - \frac{J_{\mathrm{env}}}{t_{\mathrm{w}}}, \end{aligned}$$

with $t_{\mathrm{w}}$ representing the wind braking timescale and $t_{\mathrm{c}}$ the hypothetical coupling time scale between the two zones (possibly due to the simultaneous action of physical processes such as waves, magnetic fields, turbulence, waves, or stresses). These equations further consider that the applied surface torque is instantaneously transmitted to the base of the convective envelope.

**Fig. 25:**
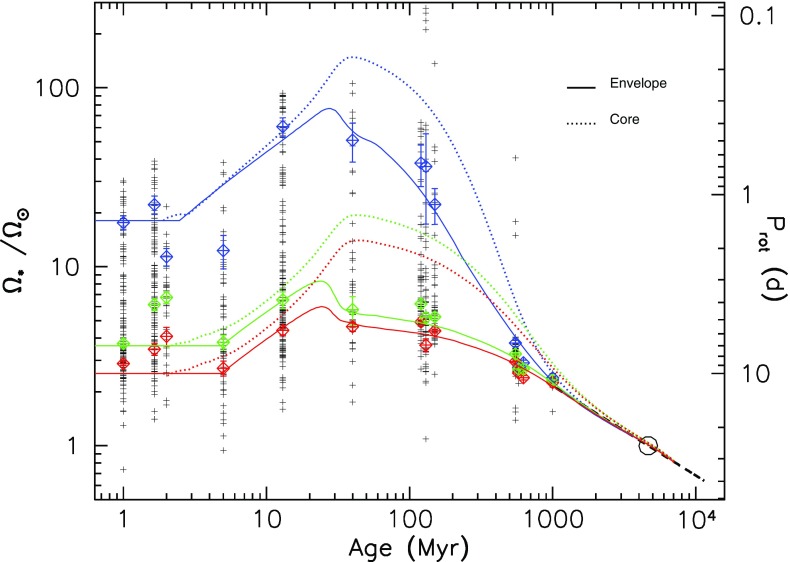
Stellar rotation history shown using observations of open clusters and 2-layers stellar rotation models. The *solid* and *dash lines* correspond to the convection and radiative interior rotation evolution. 3 initial rotation rates have been considered to represent the initial large spread of stellar rotation rates. We note the convergence of the rotational evolution curves by the age of the Sun (shown as an *open circle*). Image reproduced by permission from Gallet and Bouvier (2013), copyright by ESO

These two-zones models have been successfully used to assess the required coupling time scale between the radiative interior and the convective envelope in solar-like stars over the course of their evolution to explain the rotational evolution of stars and the core-envelope coupling in young open cluster stars (MacGregor and Brenner 1991; Keppens et al. 1995; Krishnamurthi et al. 1997; Allain 1998) and (for recent developments see Denissenkov et al. 2010; Bouvier 2013; Gallet and Bouvier 2013; Oglethorpe and Garaud 2013; Zhang and Penev 2014). These studies converge towards time scale of the order of tens to hundreds of Myr (see Fig. 25).

**Fig. 26:**
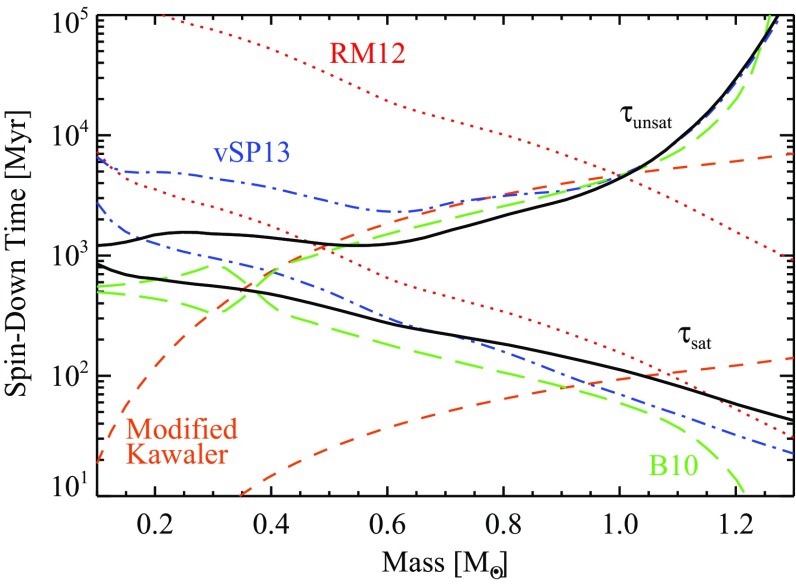
Stellar spin-down time scale in Myr for saturated and unsaturated rotation state versus stellar mass. Various models are shown, starting from that of Kawaler (1988) using an *orange dash line*, Reiners and Mohanty (2012) (*red dotted line*), Barnes (2010) (*green long dash line*), Saders and Pinsonneault (2013) (*blue dash dotted line*) and Matt et al. (2015) (*solid black line*). Note that 3 group of curves (*green*, *blue* and *black*) out of 5 are showing the same overall behavior: a longer spin down time scale for increasing stellar mass in the unsaturated state and by contrast a decreasing time scale in the saturated rotational regime. Image reproduced by permission from Matt et al. (2015), copyright by AAS

Another important property deduced from observations of the rotation rate of stars in open clusters is that their spin down time scale seems to depend on stellar mass. For instance, it is observed that F-type stars spin down faster than M-type stars until they reach the converged sequence (e.g., when the spread in rotation rates in the open cluster has become very small) as defined in Barnes (2003, 2010). On that converged sequence, when stars are no more in the saturated rotation regime, the braking time scale seems to be larger for F-type stars. Such a change of behavior could for instance be explained by different levels of braking efficiency by stellar winds. In Fig. 26 we illustrate that complex state of matter by showing various theoretical or observational trends for stellar wind torque found in the literature, e.g. Kawaler (1988), Reiners and Mohanty (2012), Barnes (2010); Saders and Pinsonneault (2013), Matt et al. (2015).

These models help understanding the rotational evolution of stars in the large, but for instance for low mass stars some difficulty remains (Brown 2014). Recent asteroseimology studies using *Kepler* data, have shown that *gyrochronology* may break for old stars, possibly due to a less efficient wind braking (Davies et al. 2015; van Saders et al. 2016). Large systematic changes in field intensity and geometry may be the source of this phenomena (Réville et al. 2015a, b; Vidotto et al. 2016; Garraffo et al. 2015a).

**Fig. 27:**
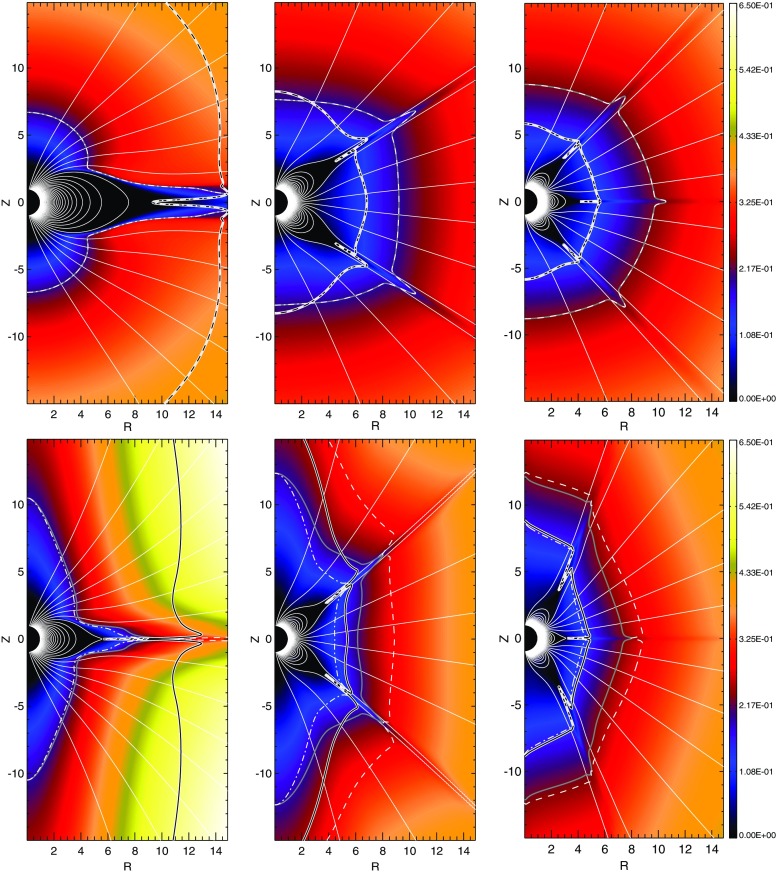
Influence of rotation and magnetic geometry on stellar wind structure and velocity profile. From *left* to *right*: We show as color contours of the poloidal wind velocity (normalized to the solar surface Keplerian speed of 437 km/s) the solution for Dipolar, Quadrupolar and Octupolar geometries at 2 rotation rates (field lines are shown as *white solid lines*). *Top row* slow rotators, *bottom row* fast rotators. Notice how the Alfvén surface (*white line with black core*) changes, getting closer to the star near the equator and further away near the poles due to magnetic towering effects. Note also the collimation of the magnetic field line at high latitude due to the pressure gradient of the longitudinal field. Finally note higher is the multipole closer is the Alfvén surface at the equator. Image reproduced by permission from Réville et al. (2015a), copyright by AAS

In order to better describe the angular momentum transport within and outside stars multi-D simulations are a useful complementary tool. Solar MHD wind simulations have been developed for several decades, generalizing the equatorial model of Weber and Davis (1967) and solving numerically the 2-D polar solution following the work of Sakurai 1985. Most studies have assumed either a split monopole or a dipole (Kawaler 1988; Keppens and Goedbloed 1999; Matt et al. 2012). However as we have seen in Sect. 2, observations of stellar magnetic fields clearly reveal many more modes such as quadrupole, octupole, etc. More recent studies have thus considered complex, multipolar field geometries (Matt and Pudritz 2008; Cohen et al. 2009; Jardine et al. 2010; Cohen and Drake 2014; Réville et al. 2015a, b; Garraffo et al. 2015b; Vidotto 2016). This has lead to new formulations of the spin down torque through stellar wind (Réville et al. 2015a). In Fig. 27 we represent stellar wind solutions for various field geometries (dipolar, quadrupolar or octupolar) or rotation rates (solar-like or fastly rotating young suns).

It can be seen that the Alfvén surface changes significantly:

- It gets closer to the star as the field geometry becomes more complex. This is easily understood by noting that the amplitude of an octupolar field decreases much faster with radius than the dipole, so that a stellar wind with fixed driving reaches the Alfvén speed sooner (i.e., closer to the star) in the multipolar case.
- It also moves closer to the surface due to the magneto-centrifugal effect that contributes more and more efficiently to accelerate the wind the faster the star rotates. This effect is just analogous to the motion of a bead free to move on a swinging rope: it will tend to move to its end with a speed that will increase as the rope rotates faster and faster.

**Fig. 28:**
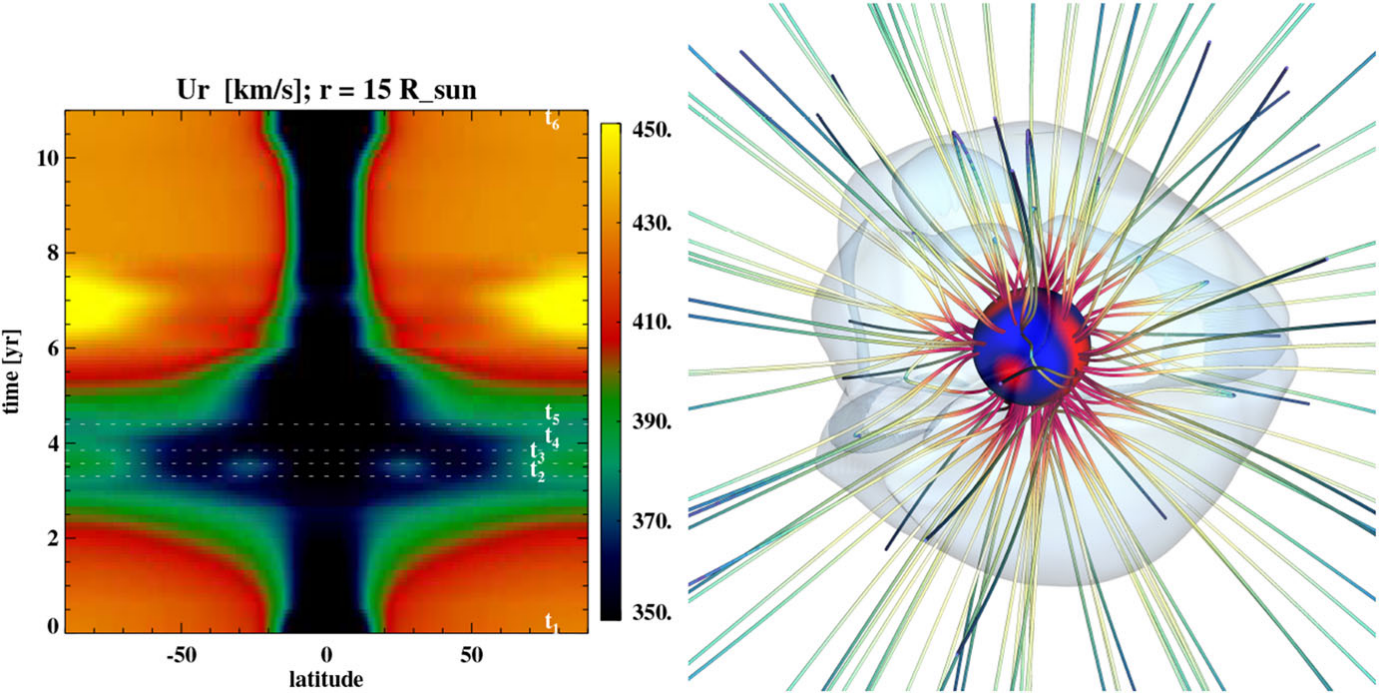
*Left* 2.5-D simulation of the solar corona and wind along a 11-year magnetic cycle computed with a mean field solar dynamo model. Shown is the solar wind speed computed at 15 solar radius, with *dark tones* denoting slow speed. Image reproduced by permission from Pinto et al. (2011), copyright by AAS. *Right* 3-D simulation of the stellar wind of HD189733 using realistic spectropolarimetric maps as boundary condition (Strugarek et al. 2014; Fares et al. 2013). Magnetic field lines are *color-coded* by the field amplitude and the Alfvén surface represented by the *light blue* surface

More complex field geometry also makes the location of coronal holes moves to lower latitudes and will modify the location of fast and slow wind streams. Generalisation of solar wind models to various stellar spectral type using a large range of global stellar parameters have been undertaken in 2.5D by Suzuki et al. (2013), Johnstone et al. (2015), Réville et al. (2015a). With as many as 60 stellar wind models, the parameter study cover several orders of magnitude in field amplitude and geometries and rotation rates getting close to break up values. More recently 3-D stellar wind simulations have been performed, often by including spectropolarimetric map as surface boundary conditions. They reveal a convoluted Alfvén surface and even ultra fast stellar winds originating from latitudinally and longitudinally extended coronal holes. (see, e.g., Vidotto et al. 2009; Cohen and Drake 2014; Vidotto et al. 2014b; Strugarek et al. 2015, 2015b; Réville et al. 2016, and Fig. 28 (right panel)).

Another important ingredient of realistic stellar wind model is the physical description of the acceleration region, e.g., the chromospheric layer and how heating and cooling sources and treated. In most MHD wind models based on Parker’s initial description of an expanding isothermal coronal, the temperature is fixed to a value around 1.3 to 2 *MK* and the polytropic index $\gamma$ is set to 1. Polytropic generalisations have been derived and implemented (Keppens and Goedbloed 1999) allowing use of a value of $\gamma$ that is slightly higher (1.05 to 1.1). This results in terminal wind speed around 400 to 600 km/s which is often a bit low to describe the fast stream of the solar wind (Johnstone et al. 2015).

Using a small value of $\gamma$ is a numerical recipe, adopted in order to avoid considering realistic heating and cooling sources; but we know that this is not what is occurring in the chromosphere and low corona of stars. Their winds are better described by complex heating (for instance, by Alfvén wave energy injection) and cooling functions (due to conduction or radiative loss for instance) and by using a polytropic index closer to its adiabatic value. Hence, a better description of the thermodynamics of the low corona and transition region is key. Some first attempts to do so have been reported in Schwadron and McComas (2003), Suzuki and Si (2006), Cranmer and Saar (2011) and references therein.

Stellar winds also change on short time scales, as for instance in the Sun during the rising and declining phases of the 11-year cycle (see Fig. 3), when the number and location of active regions, streamers and coronal holes change continuously. PFSS-like models easily reconstruct the large scale coronal magnetic field geometry, by setting an open source surface at 2.5 solar radius. This value is actually found to change during the solar cycle and for various stellar parameters (Lee et al. 2011; Réville et al. 2015b). It is also shown that Potential field source surface extrapolation of the coronal field overestimates the expansion factor ($f=\frac{A1}{A0} \left( \frac{r0}{r1}\right) ^2=\frac{B0}{B1} \left( \frac{r0}{r1}\right) ^2$; with $A0,\, A1$ the flux tube surface area at the surface and far in the wind, $B0,\, B1$ the magnetic field at the same locations and $r0,\, r1$ the surface and distant radius) which is one of the key ingredients for determining the stellar wind terminal velocity, see, e.g., Wang and Sheeley (1990), NOAA’s WSA-Enlil model (Arge and Pizzo 2000; Mays et al. 2015) and Pinto et al. (2016). More accurate solar wind models have also been computed over an 11-year cycle, showing that the Alfvén radius and torque exerted by the solar wind changes by a factor of 3 between the minimum and maximum phases of the activity cycle (Pinto et al. 2011, 2016; Réville et al. 2016). As shown in Fig. 28(left panel) these coupled dynamo-wind models reproduce qualitatively well the variations seen in IPS radio maps of Tokumaru et al. (2010) and Manoharan (2012), and with the estimations by Wang and Sheeley (2006) using ULYSSES data and semi-empirical methods. A recent analysis of 5 years of IBEX satellite data also indicates that solar wind speed variation with respect to the heliographic latitude are compatible with the change of solar magnetic field geometry generated by dynamo action along the 11-year cycle (McComas et al. 2014). Note that attempts to reconstruct the solar wind conditions over the past four centuries have also been pursued by Owens et al. (2017).

## Simulations of stellar magnetism and rotation

**Fig. 29:**
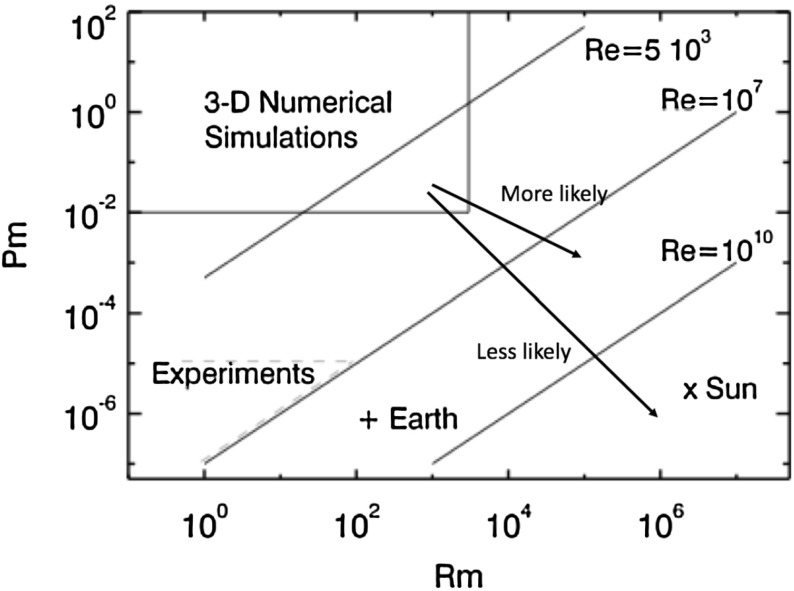
Magnetic Prandlt versus magnetic Reynolds numbers parameter space diagram. Numerical simulations are in the *upper left corner*, while laboratory experiments are in the *lower left*. The Sun is in the *lower right corner*, the most extreme one. More likely and less likely route for future numerical simulations are tentatively indicated

A star’s magnetism is, as we have seen in previous sections, the result of many competing processes. Convection, rotation (differential or otherwise), buoyancy, a complex array of surface effects, and stratification all play roles in some stars, and in many cases it is not possible to sort out analytically just how these will all combine to generate the magnetism. The character of the field—its strength, its temporal and spatial variability, its morphology, and so forth—are constrained by dynamo theory, but (usually) not uniquely predicted by it. Any theoretical model must also contend with the intrinsically chaotic nature of some of the processes that build the magnetism: even if there is one day a universally accepted theory of the 22-year solar cycle, there will always be “solar weather”, and so too on other stars. Faced with these difficulties, many workers in the field have turned to numerical simulations of the governing equations as a tool for understanding the types of fields that might be built in given situations. Though these simulations have problems of their own—in particular, they must for numerical reasons operate in parameter regimes very far from those that prevail in stars as shown in Fig. 29—they can nonetheless serve as useful tools for testing basic conceptual models of field generation, for interpreting observations at some level, and ultimately for building intuition about the processes by which stars build magnetic fields. In this section, we describe some of these simulations and the ways in which they have altered or confirmed our understanding of the dynamo process.

### Unifying physics and methods

At a certain level of abstraction, all stars share certain unifying physical features that likely control the production of magnetic fields: they rotate, they are spherical, they are generally very good conductors throughout most of their interiors. Many groups have therefore turned to simulations that explore field generation in idealized objects sharing these basic properties, in the hope that many features of the idealized problem may prove to be robust, while necessarily ignoring other attributes peculiar to one type of star or another. Other models have chosen to focus on specific objects or problems in more detail, capturing (for example) surface granulation with great fidelity, while missing other aspects (e.g., the overall spherical geometry). We will discuss examples of both types of simulations below, but have chosen to organize our discussion by object (i.e., by the spectral type of the object the simulations are supposedly addressing), while recognizing that many of the same physical principles are present in all these simulations (as they are in all stars). We have chosen this organisational approach partly because so many different elements change in going from one type of object to another—aspect ratio, location of the convection zone, level of energy input, etc.—that results derived in one context are not always relevant or useful in another. Further, many authors have chosen to frame their simulations as being relevant to one type of object or another, rather than as abstract fluid mechanical problems, and our categorisation below reflects this. We must caution, though, that while the basic results of any given simulation are not usually controversial (most of the dynamo codes in use today are solving the MHD equations under similar approximations), their relevance to stars or planets is less clear-cut. We will return to this issue in specific instances below.

First, we recall the most basic properties that, from a fluids perspective, separate one star from another. Though essentially the same processes operate at some level in all convective stars, the balance between them changes. Particularly great effects appear to come from the geometry of the system, from the relative influence of rotation relative to other effects (buoyancy driving or viscosity, for example), and from stratification. In the Sun, for example, convection occurs in a shell occupying the outer 30% of the star; rotation probably plays an important but not utterly dominant role in establishing the field; the stratification is fairly strong (i.e., convection extends over many density scale heights from the tachocline to the photosphere). In a fully-convective M-dwarf, by comparison, the same elements are all present but their relative importance is altered: convection occurs in the full sphere (and is weaker, since it is required to carry less energy outwards), rotation is usually much more significant in the dynamics, and the stratification is quite strong. Other, subtler effects related to heat transport can play roles as well: in young stars and very low-mass objects, for example, the luminosity is partly from gravitational contraction and is a strong function of time, and we might expect this to lend some peculiarities to the dynamo process. (This, for example, means that a fully convective M-dwarf is not precisely analogous to a pre-main-sequence star, though the two share many similarities.) The relative roles of radiative diffusion and convection also vary with mass—in the Sun, for example, mixing length theory (and the simulations below) suggest that the efficient envelope convection carries almost all of the Sun’s flux outwards within the envelope, with radiative diffusion playing a negligible role there; meanwhile in M-dwarfs, radiative transport is in principle capable of transporting a significant fraction of the stellar luminosity in some regions, even though the interior is fully convective. (Put another way: even an isentropic low-mass stellar interior would, assuming radiative opacities from a typical 1-D stellar model, have a non-negligible radiative flux in some regions.) These broad differences help motivate our discussion of simulations below.

### Overview of computational approaches

Before describing the results of these simulations, a few comments on the numerical methods and codes used to produce them are appropriate. Historically, many workers studying turbulent flows have turned to *spectral methods* (e.g., Gottlieb and Orszag 1977; Canuto et al. 1988; Hussaini and Zang 1987), in which the flow variables are represented by a weighted sum over certain basis functions, with derivatives then obtained using appropriate recursion relations (see, e.g., discussion of this and other numerical methods in Rogallo and Moin 1984). Broadly, these have long been attractive because of their excellent convergence properties: for smooth functions they converge exponentially as the number of modes is increased (in contrast to, e.g., finite-difference methods, whose convergence scales with the grid spacing to some power). However, they are typically less well-suited to problems with sharp discontinuities (e.g., shocks), and can become less attractive as the problem size becomes very large (in which case the transforms between physical and spectral space can dominate the computational workload). Still, for convection in main-sequence stars or planets—which remains comfortably subsonic in most instances—spectral methods remain very popular, and many of the results quoted below employ this basic technique, though finite-difference and finite-volume methods are also in use. (Conversely, many codes developed for broader astrophysical use go to great lengths to capture shocks or other discontinuities, but do not converge as rapidly with increasing resolution. For a recent summary of some of these, see Hopkins (2015).) A brief description of several of the codes in broad use today can be found in Sect. 6.1, and a more thorough summary can be found in Brun et al. (2015b) (their Sect. 3).

A central tenet of fluid dynamics is that the solutions to many seemingly disparate problems “collapse” to the same solution when viewed in terms of certain nondimensional parameters: e.g., for flow in a pipe, the individual values of velocity *u*, length *l* and viscosity $\nu$ are less relevant than their combination as a Reynolds number $Re = ul/\nu$. The same is true in principle for the simulations surveyed below; what matters for the flow field are the values of *Ra*, *Ek*, and so forth (see Table 1 for definitions), and indeed many simulations are conducted using non-dimensional codes and rescaled after the fact to provide some contact with a given physical object. (This procedure is straightforward for standard problems, e.g., Rayleigh–Bénard convection with an imposed temperature gradient, but can be much less clear-cut in other cases.) Viewed in this way, current “state of the art” global simulations, for example, attain Ekman numbers of order $10^{-6}$, Rayleigh numbers of order $10^9$, and Reynolds numbers of order a few 1000. Clearly these values are each orders of magnitude below those that prevail in stellar interiors, so much of the “art” (and most of the debate) of stellar convection simulation lies in assessing which results in numerically accessible regimes are likely to be robust even when the relevant control parameters are changed enormously. In our summary of simulations below, we have generally chosen to present, first and uncritically, what different simulations have shown, and only later to comment on *why* so many different solutions have been found, and what this implies for the magnetism of real stars. Such commentary can be found, for example, in Sect. 6.1.3, in the closing portions of Sects. 6.3 and 6.4, in Sect. 6.5.4, and in Sect. 7.

### The Sun

The Sun, and fluid convection in stars and planets generally, was one of the earliest targets of numerical simulation in astrophysics, and an early application of numerical MHD specifically. From the 1960s onward, using the equations of MHD solved on a computer, researchers have sought to understand both global features of the Sun’s magnetism—why it has an 11-year cycle, why sunspots propagate in latitude, why the equator rotates faster than the poles—as well as more specific aspects of the observed field. This array of research tasks has required a commensurate array of computational approaches: some models choose to focus on a localized region and incorporate radiative transfer (allowing remarkably detailed comparison with observations), while others have adopted a coarser description of the dynamics (allowing simulations that extend over larger spatial and temporal intervals). We will focus in this review primarily on the global-scale simulations, noting only a few key results from smaller-scale (and more realistic) calculations. This is, we hasten to add, not because the latter are less useful or illuminating—indeed, the agreeement between observations and simulations of near-surface convection, for example, is stunning. Rather, these models have already been thoroughly described in other recent reviews—see, in particular, Nordlund et al. (2009)—and we see little reason to repeat this here. Further, some of the most interesting features produced by small-scale surface simulations are not yet being probed by observations of stars other than the Sun, and so (for now) fall outside the purview of this “solar-stellar connection” review; in contrast, the large-scale features of other stars’ magnetism (and the global-scale flows that accompany them) have been targets of observational scrutiny for decades.

#### Historical survey of simulations and codes

Global simulations of solar convection began with the calculations by Peter Gilman and collaborators (e.g., Gilman 1975, 1977; Gilman and Miller 1981; Gilman 1983; Glatzmaier 1985). At first the models were Boussinesq and linear; later calculations, beginning with the work of Gary Glatzmaier (Glatzmaier 1984), adopted the * anelastic approximation* (Ogura and Phillips 1962; Gough 1969), which essentially filters out sound waves but includes the overall density stratification. (For recent discussions of the anelastic approximation and other “sound-proof” methods, see the monograph by Glatzmaier (2013), and the analyses of Vasil et al. (2013), Brown et al. (2012). The flows modeled were complex and time-dependent, even if still fairly laminar. With the advent of increasing computational power, simulations began to explore flows less constrained by the effects of viscosity and (thermal and magnetic) diffusivity, and to encompass stronger density stratifications. The basic approach pioneered by Gilman and Glatzmaier has continued to flourish in the past few decades, and several codes in wide use today borrow at some level from this legacy: e.g., the Anelastic Spherical Harmonic (ASH) code (Clune et al. 1999; Miesch et al. 2000; Brun et al. 2004) was developed within Juri Toomre’s group at Colorado and has been used for dozens of papers on stellar convection; the Magic code (Wicht 2002; Gastine and Wicht 2012), used more widely in the planetary dynamo community, also descends from a version of the Glatzmaier code. A few other anelastic codes were developed independently (e.g., the Leeds code, see Jones and Kuzanyan 2009), and adopt distinct numerical methods, but follow a similar model. The recently-developed Rayleigh code (described in Featherstone and Hindman 2016a) also adopts the same basic principles as these earlier code, and is (as of this writing) planned for public release in 2017. Other groups have tackled the global-scale convection problem using fully compressible methods—see, e.g., (Käpylä et al. 2011, 2012, 2013; Warnecke et al. 2014), who model a spherical “wedge” geometry using the publicly-available Pencil code—or so called ying-yang computational grid (Kageyama and Sato 2004; Masada et al. 2013) or implicit-LES calculations (e.g., Ghizaru et al. 2010; Racine et al. 2011; Beaudoin et al. 2013; Passos and Charbonneau 2014, using the EULAG code), or low-Mach number solvers (Gilet et al. 2013). Hotta et al. (2015) have also recently calculated global solar convection simulations using a reduced sound-speed method. Broadly, there has been a pleasing concordance between the results from these different groups and codes: all agree, more or less, on the sense of angular momentum transport in various parameter regimes, all agree that both cyclical and steady solutions to the dynamo problem are possible in some cases, and so forth. Though it is sometimes difficult to compare results from the models precisely—e.g., because diffusion is an implicit consequence of the numerical scheme in some simulations but modeled explicitly in others—this basic agreement is encouraging and has been confirmed by a dedicated international benchmark (see details in Jones et al. 2011).

One may categorize global simulations of Solar-like convection the simulations below as falling into a few “evolutionary” stages: first the Boussinesq cases (e.g., Gilman 1977), then anelastic models fairly close to the onset of convection (e.g., Glatzmaier 1985), followed by increasingly complex simulations with and without magnetism (Miesch et al. 2000; Elliott et al. 2000; Brun and Toomre 2002; Brun et al. 2004; Miesch et al. 2008). In most cases these have modeled only the convective unstable envelope, but some calculations (e.g., Miesch et al. 2000; Browning et al. 2006; Ghizaru et al. 2010; Brun et al. 2011; Masada et al. 2013) have incorporated an underlying stably-stratified region as well, and/or some aspects of an overlying “atmosphere”, incorporated simply as a polytropic layer of different index (e.g., Warnecke et al. 2013).

#### The development of large-scale fields and magnetic cycles

The simulations described here seek to capture some of the large-scale attributes of solar flows and magnetic fields. But it is worth noting explicitly what we mean by “large-scale”. Every simulation resolves only a finite range of spatial scales, from the overall size of the system being modeled down to a smaller level set by numerical limitations. To claim a “large-scale” field has been generated, one would generally like to see fields with a coherence length much greater than that of the smallest (or indeed the dominant) scales of motion in the system; similarly, we must distinguish between the temporal behavior associated with “small-scale” dynamo action (which might well show chaotic reversals of polarity, on a timescale related to the small-scale flows) and orderly polarity evolution over times much longer than the convective time (but shorter than the diffusive time, if the latter were determined by microphysics alone). The Sun, of course, manages both these tasks: it builds an ordered dipole field (with spherical harmonic degree $l=1$) whereas the convective eddies visible at the surface as granulation have $l > 100$, and it displays an orderly 11-year cycle rather than a chaotic wandering (in contrast, for example, to the geodynamo). The notion of “scale separation” thus underpins much of our discussion below: indeed, many of the active debates in this field revolve around the interaction between “small-scale” and “large-scale” fields. In a numerical simulation there is not often a clear dividing line between the two, and many “small-scale” timescales (having to do with the flows) are for numerical reasons uncomfortably close to other potentially relevant timescales (e.g., the diffusion time). With this caveat firmly in place, we note some features that have emerged robustly from a variety of simulations.

**Fig. 30:**
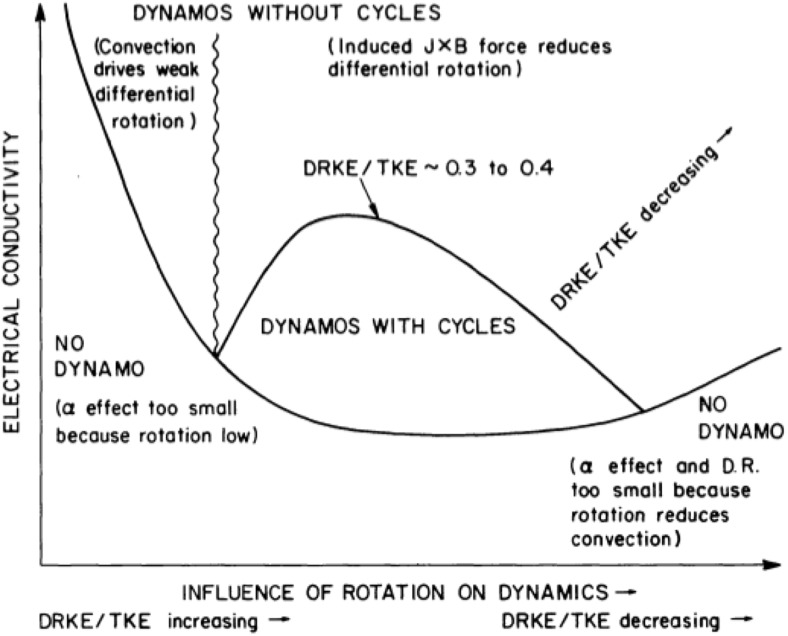
A speculative “regime diagram” for the interplay between differential rotation, conductivity, and the presence of magnetic cycles. Image reproduced by permission from Gilman (1983), copyright by AAS

The earliest simulations (Gilman and Miller 1981; Glatzmaier and Gilman 1982; Gilman 1983; Glatzmaier 1985) exhibited global-scale flows and dynamo action. Some of these simulations showed cyclical polarity reversals and latitudinal propagation. The cyclical dynamos were distinguished from non-cyclical ones partly by whether strong differential rotation was present: simulations in which the zonal flows were too weak, either because of Lorentz force feedbacks or dissipative effects, tended to have more chaotic magnetism. Figure 30 exhibits a speculative regime diagram, from Gilman (1983), based on some of these results. In a sense, one of the achievements of subsequent simulations has been to test this possible regime diagram, confirming it in places and not in others (as discussed in more detail below).

Field propagation in the cyclical simulations was towards the poles—in agreement with the prediction of the classic Parker–Yoshimura sign rule (Parker 1955a; Yoshimura 1975; Stix 1976) for this combination of kinetic helicity and differential rotation, but opposite to what is observed in the Sun. The cycles also tended to be either irregular (Gilman and Miller 1981) or, if orderly, to have periods that were much shorter than the observed solar cycle (e.g., Gilman 1983; Glatzmaier 1985). Later global models captured increasingly turbulent flows, and greater scale separations between the overall geometry and the smallest resolvable length and time scales, but the result was not always greater agreement with the observed Solar field. Instead, some of the most turbulent models (Brun et al. 2004) exhibited magnetism that reversed in polarity chaotically, over brief (few hundred day) intervals, and which exhibited relatively little large-scale organization. In these simulations, the mean (axisymmetric) fields were about 2–3% of the total magnetic energy, representing about 10% of the kinetic energy relative to the rotating frame. Boundary conditions clearly played a role—for example, simply running simulations akin to those of Brun et al. (2004) but with different bottom boundary conditions (Brown 2009) resulted in more ordered fields.

**Fig. 31:**
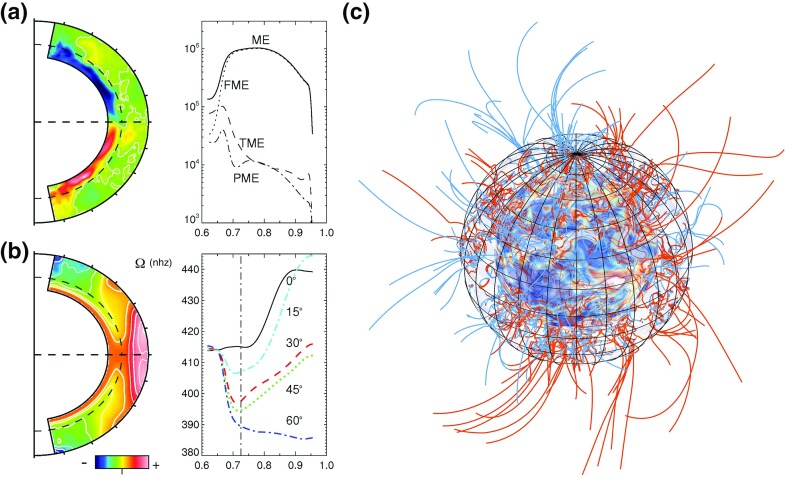
**a** Averaged toroidal field in the dynamo model published in Browning et al. (2006); **b** Differential rotation in same case and **c** magnetic field line reconstruction within the tachocline, convective zone and in a vacuum atmosphere (potential extrapolation using PFSS package adapted to ASH)

Motivated by the “interface dynamo” paradigm discussed in Sect. 5, some simulations also attempted to capture the transport and amplification of magnetic fields within the solar tachocline, albeit in a highly simplified fashion. The models of Browning et al. (2006), Browning et al. (2007), for example, included both the convective envelope and a stably stratified region below it. A simplified “tachocline” was essentially imposed, by forcing the stably stratified region towards solid-body rotation while allowing differential rotation to be established self-consistently within the convection zone. The magnetism in these simulations was intense and small-scale within the convection zone, but was accompanied by somewhat larger-scale, more organized structures (with clear antisymmetric parity) below its base. A sampling of these results is shown in Fig. 31, which displays the differential rotation, radial variation of azimuthally-averaged “mean” fields, and magnetism (with an extrapolation to a potential field source surface) in these simulations. These simulations did not, however, show any reversals of polarity at all, much less an orderly cycle. Later simulations by Paul Charbonneau and collaborators, employing the implicit large-eddy code EULAG, and likewise incorporating both a convection zone and a stable layer below it, (Ghizaru et al. 2010; Racine et al. 2011; Beaudoin et al. 2013), did yield clear magnetic cycles: the simulations show large-scale fields that reverse in polarity over timescales of about 36 years. The fields imprint through both the convective envelope and part of the stable region below it, and are strongest just below the interface between these regions. These represented, at the time, the closest contact any global simulation had yet made with the cyclical solar dynamo. But several major discrepancies with observations persisted: most significantly, no equatorward migration of the magnetic field was obtained, and the poloidal and toroidal fields appeared to oscillate in phase (whereas in the Sun they are phase-lagged). Other simulations including a simulated tachocline have likewise produced cyclical fields—see, e.g., Masada et al. (2013), Mabuchi et al. (2015) and Guerrero et al. (2016). Guerrero et al. (2016), in particular, compare simulations with an underlying stable layer to those without, and find that the former evolve on much longer timescales than the latter. Below (and in Sect. 6.2), we comment briefly on *why* the simulations sampled here yield a range of different behaviour, including cycles in some cases and steady fields in others.

#### Some recent developments and general principles

A wide variety of simulations, intended to model global-scale Solar convection and magnetism in various ways, have (as summarized above) yielded magnetic fields with large-scale spatial and temporal organization. Here we discuss a few broad issues raised by such simulations, and highlight some particularly recent developments that bear on these issues.

First, note that while some of the simulations quoted above suggest that a tachocline of shear may be *helpful* for building large-scale organized fields, it is equally clear that (within the parameter regimes probed by many global-scale simulations) organized fields are sometimes possible * without* this layer, too. Examples (going back to the early work by Gilman and collaborators) abound. In Brown et al. (2010) and Brown et al. (2011), for example, discussed in more detail in Sect. 6.2, strong bands of toroidal field are generated amidst anelastic simulations of a solar-like convection zone (rotating more rapidly than the Sun). As another recent example, Käpylä et al. (2012) found cyclic polarity reversals and clear equatorward propagation, both on decadal timescales, in simulations that modeled a “wedge” of a spherical convective envelope.

Why some of these simulations have cycles and others do not, and what sets the period of any cycles that are present, is not yet well understood. Broadly, it appears that flows subjected to stronger rotational constraints have an easier time building ordered fields that (in some cases) are cyclic; in a dimensional code with explicit viscosity, “stronger rotational constraints” means either increasing the rotation rate or decreasing the buoyancy forcing (whether applied via a fixed flux boundary condition, or via a fixed entropy or temperature contrast across the layer). Because some codes model dissipation implicitly, determining the effective “Rayleigh number” is not always an easy matter, and this complicates comparison of results produced with different codes. For example, some of the differences between the cyclical “Solar” calculations noted above (Ghizaru et al. 2010; Racine et al. 2011; Beaudoin et al. 2013) and the non-cyclical models (Browning et al. 2006) may arise because the influence of rotation is (by virtue of the numerical methods adopted) somewhat stronger in the former simulations. See Strugarek et al. (2011) for a more detailed analysis, and also discussion below.

More generally, the simulations noted above produced some “large-scale” field component, and in some cases this large-scale field even behaves roughly as suggested by mean-field theory: see, for example, the analyses of Racine et al. (2011), Warnecke et al. (2016), Simard et al. (2016). That is, it shows some of the symmetry properties expected from simple mean-field models, it exhibits cycles and latitudinal propagation that obey some form of the Parker–Yoshimura rule, its production is linked (in part) to the helicity of the turbulence, and so forth. But all other things being equal, more complex flows often produce more complex fields: i.e., one might expect to have some magnetism on every scale where there is flow. Determining whether the nonlinearly saturated state in the numerical simulations, which are capturing only the largest scales of motion, bear much resemblance to the state that would result at much higher *Rm*—and if so, why—is not an easy matter.

There has been reason for some skepticism about whether the results of such simulations are relevant to the Sun. For example, in well-resolved simulations the mean field (as measured relative to, say, the equipartition strength, $\langle B \rangle / B_{\mathrm{eq}}$ is typically a strong and declining function of *Rm*: see, e.g., Schrinner et al. (2012), Karak et al. (2014), Simard et al. (2016), Warnecke et al. (2016) in the global context, Brandenburg (2008) in the context of a (Cartesian) Beltrami flow, or Cattaneo and Hughes (2006), Cattaneo and Hughes (2009), Hughes and Proctor (2009), Hughes et al. (2011), Favier and Bushby (2013) in the context of Cartesian convection. Further, the large-scale field in the simulations often equilibrates on a diffusive time (see, e.g., Brandenburg 2001). Some of the results are reminiscent of the theoretical expectation of “catastrophic” $\alpha$-quenching (Vainshtein and Cattaneo 1992), in which the *flux* generated by dynamo action is a negligible fraction of the magnetic *energy.* Extrapolation to the enormous *Rm* values attained in stellar interiors would then imply only negligible mean fields could be built. While such quenching might be alleviated by fluxes of magnetic helicity (from one portion of the domain to another, or through boundaries)—see, e.g., Low (2002), Hubbard and Brandenburg (2012), Blackman and Hubbard (2014), Ebrahimi and Bhattacharjee (2014), review in Blackman (2015)—these fluxes are, at least in the simulations that are currently tractable, often small compared to other effects. Moreover, it is generically true that we expect small-scale dynamo action to occur at sufficiently high *Rm*, yet such dynamo action isn’t even a part of standard mean-field-theory; nor is dynamo action on very small scales explicitly captured in many numerical simulations that resolve the “global” scales. (In MFT, as discussed above, the small-scale field is linked to the large-scale one: if $\langle B \rangle =0$, then the small-scale field vanishes as well. If there is a small-scale dynamo, though, then this need not be the case.) In at least some cases, it is clear that the small-scale dynamo may “overwhelm” the large-scale one, with any residual large-scale field just a transient sum over uncorrelated smaller fields (Cattaneo and Hughes 2009). How the small scale fields interact with large-scale ones in more realistic environments, with shear, overall rotation, and open boundaries, is far from clear.

**Fig. 32:**
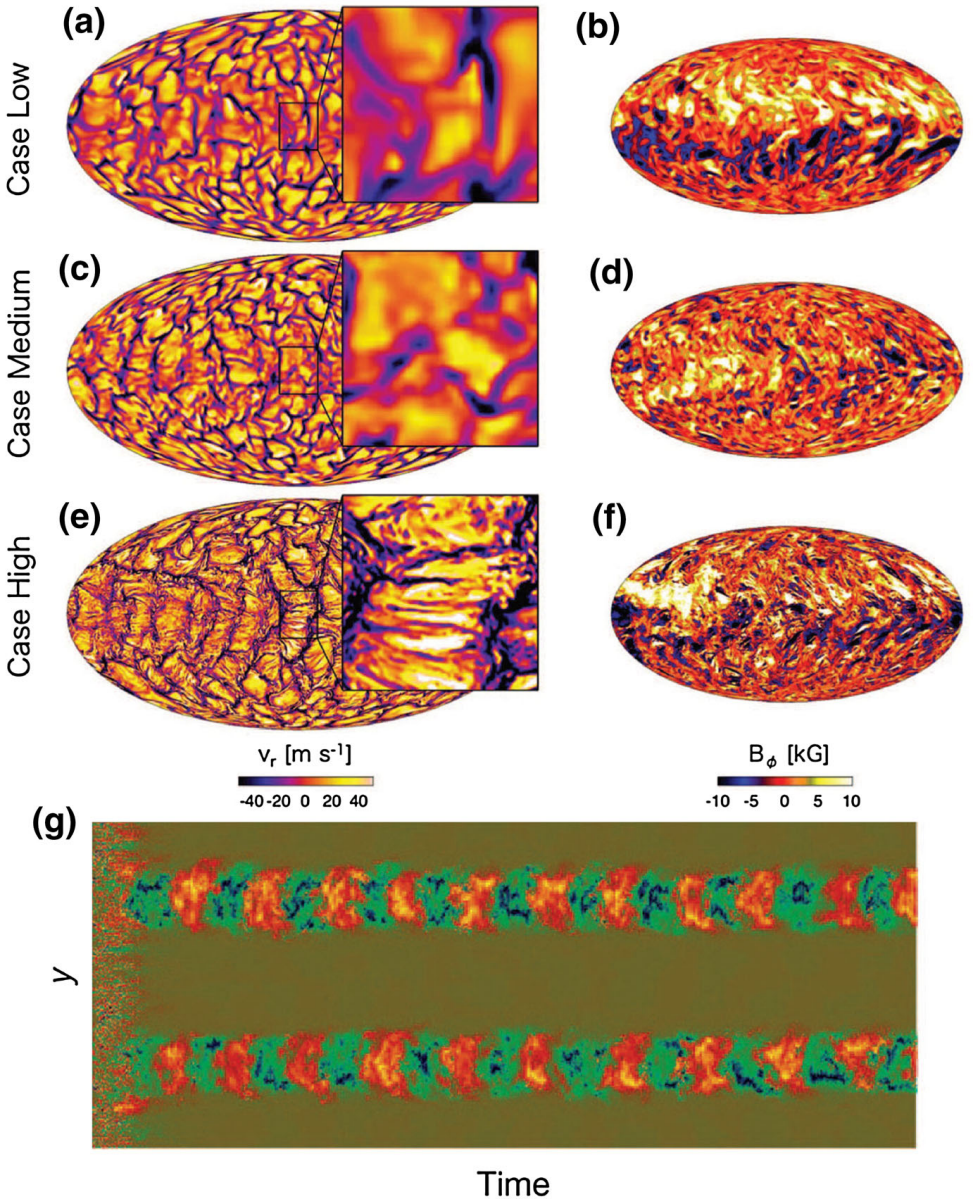
**a** Emergence (panel B), disappearance (D), and re-emergence (F) of large-scale magnetic structure in simulations of varying turbulence degrees and resolutions (for low and medium cases $Nr \times N_{\theta } \times N_{\phi } = 64 \times 96 \times 288$ and $256 \times 384 \times 1152$ for high resolution case) (Hotta et al. 2016). Shown are color contours using Mollweide projection of the radial velocity (with a zoom illustrating the increasing small scale aspect of convective flows) near the surface (*left panels*; A, C, E) and of the longitudinal component of the magnetic field near the base of the convective envelope (*right panels*; B, D, F). **b** Panel G: Large-scale, shear-driven dynamo waves in the kinematic regime at high *Rm*, shown is the toroidal field (*reddish tones* represent positive polarity) as a function of time and latitude (*y*) in this Cartesian shear-dynamo simulation (Cattaneo and Tobias 2014)

But the future of solar dynamo theory is probably not as dark—nor present simulations so divorced from reality—as some of these results might suggest. On the simulation side, Hotta et al. (2016) have recently presented simulations in which large-scale fields emerge when the flow is relatively laminar (which in their ILES code, is achieved by running at lower resolutions), diminish when the flow is more complex (higher resolution), and then—quite surprisingly—appear prominent again in their most turbulent calculations (highest resolution). These are sampled in Fig. 32. They interpret this as arising because the highest-resolution cases exhibit vigorous small-scale dynamo action, which then reacts back on the small-scale flows—essentially damping the “turbulent diffusivity” that otherwise acts to diminish the large-scale magnetism. The parameter regimes reached in these calculations are, however, still somewhat more extreme than can be achieved in most global (full-sphere) simulations. Meanwhile Cattaneo and Tobias (2014), Tobias and Cattaneo (2013) have shown, in the specific context of kinematic dynamo action by helical flow and large-scale shear, that shear can act to “suppress” the small-scale fluctuating dynamo, allowing large-scale growing modes (which are overwhelmed at high *Rm* in the non-shearing case in their example) to survive even at high *Rm*. These are also sampled in Fig. 32. Taken together with some of the results quoted above, these results suggest that all coherence is not lost as the simulations march towards higher *Rm*, and indeed that in some cases higher *Rm* might help *enable* large-scale dynamo action rather than act as an impediment to it.

To summarise: One theme that emerges from much of the above is that strong rotation (as opposed to merely *some* rotation to break symmetry) and shear are generically very helpful, and perhaps essential, to large-scale dynamo action as observed in the Sun and other stars. Many specific details about the strength of fields, their spatial morphology, and their time variability are still uncertain, but several of the basic results are not in serious dispute, and are reproduced by independent codes and groups studying disparate physical regimes. To wit: (1) More rapid rotation promotes large-scale field generation. This might seem unhelpful in the present context (we know how rapidly the Sun rotates!); but because the influence of rotation depends also on the vigor of convection, there is still considerable uncertainty about how strong a role rotation really plays, even in the Sun (see, e.g., discussion of convective amplitude in Featherstone and Hindman (2016b)). (2) The role of shear is more complex: it can act to build toroidal fields directly, but may also influence small-scale dynamo action and can disrupt the building of ordered dipole fields (as discussed more in Sect. 6.4). (3) At the present time, the results of dynamo simulations are still somewhat dependent on numerical parameters (resolution or diffusivity) that are orders of magnitude away from those in real stars. A quantitative, predictive theory that encompasses all these results is not yet available. In the following sections, we will see how these dynamical processes play out in models of other stars as well.

### Young stars

During the pre-main sequence phase stars go through important structural and global changes. Aside from the appearance of a radiative interior in solar-like stars by the time they reach the ZAMS, stars undergo a complex rotational history (see Sect. 4.3 and Figs. 11 and 25). It is well known that most young stars are fast rotators and very active with clear activity indices such as Ca ii H & K lines or high X-ray luminosity with respect to their bolometric luminosity (see Sect. 4.3Skumanich 1972; Pizzolato et al. 2003; Bouvier 2013).

**Fig. 33:**
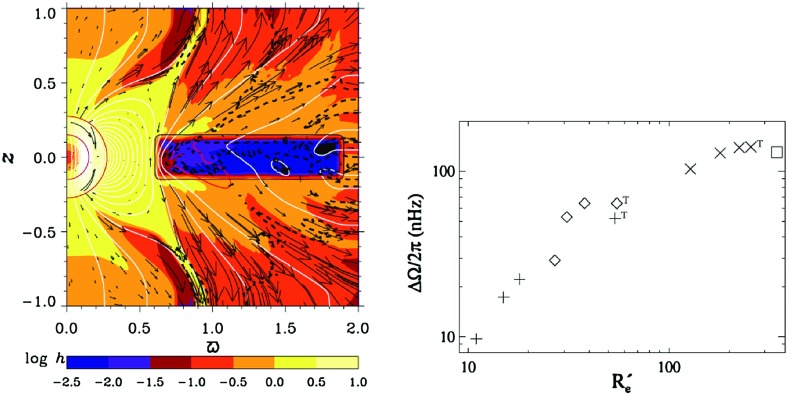
*Left* 2.5 D simulation of the coupling between a stellar dynamo and a magnetized accretion disk. Image reproduced by permission from von Rekowski and Brandenburg (2006), copyright by Wiley. *Right* scaling of differential rotation contrast versus Reynolds number in a model of a young solar-like *star*. Image reproduced by permission from Ballot et al. (2007), copyright by AAS

What impact does the high rotation rate have on turbulent convection, mean flows and dynamo action in stars?

Several groups have studied this topic with high performance numerical simulations (Ballot et al. 2007; Brown et al. 2008, 2010, 2011; Käpylä et al. 2011; Matt et al. 2011; Guerrero et al. 2013; Gastine et al. 2014; Käpylä et al. 2014; Guerrero et al. 2016). These simulations share common features with simulations of solar-like stars discussed in Sect. 6.3 and low mass stars in Sect. 6.4. Young stars rotate fast and for some period of their infancy are fully convective.

**Fig. 34:**
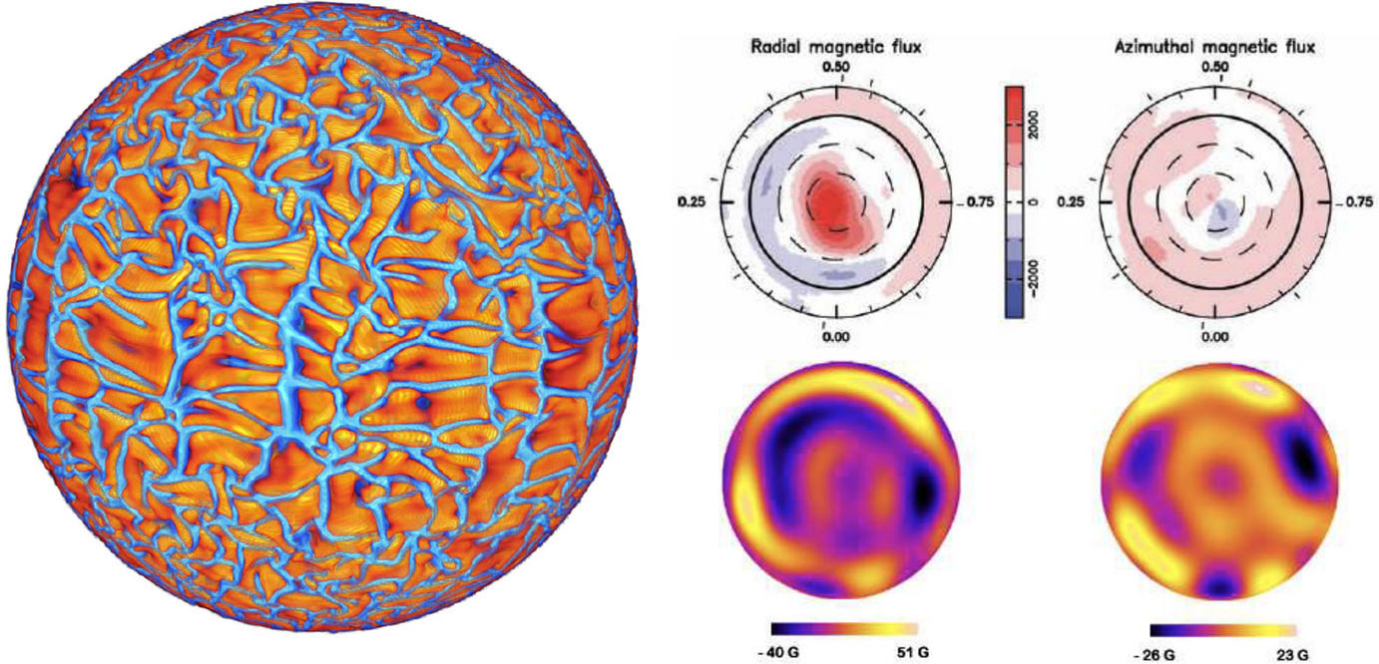
*Left* surface radial convective velocity for a young, rapidly rotating star, *red tones* correspond to upflows, from Bessolaz and Brun (2011a); *right* 3-D dynamo simulations of a young solar like-star (BPtau) and comparison with observed field (Bessolaz and Brun 2011b)

Most of the modeling efforts regarding the role of magnetic field in the PMS phase of stars involve considering their magnetic interaction with their accretion disk (Romanova et al. 2003; von Rekowski and Brandenburg 2006; Zanni and Ferreira 2013). In the 2.5-D work of von Rekowski and Brandenburg (2006) a first attempt to couple mean field dynamo with accretion physics has been undertaken in order to consider more complex and time dependent magnetic field than a pure static dipole (see Fig. 33). But generally speaking, little has been published regarding ab-initio 3-D MHD simulations of dynamo and convective states in young stars, if we wish to explicitly make a distinction with fastly rotating solar-like stars on the main sequence as discussed in Sect. 6.3. The few exceptions are:

- Ballot et al. (2007) have modelled various case of young stars with thick convective envelope and fast rotation rates. They found that differential rotation amplitudes in the models are sensitive to the degree of turbulence of the convection zone, a more turbulent state yielding a stronger differential rotation, but that effect tends to saturate (see Fig. 33, right panel). They also find that ${\varDelta } {\varOmega }$ scales not linearly with ${\varOmega }_*$ the rotation of the star, hence leading to a relatively weaker constraint for faster rotation rate, as observed (see Sects. 4.3 and 6.3). Likewise they find that meridional circulation amplitude remains at best constant but the main trends seems to indicate that it decreases in strength with ${\varOmega }_*^{\beta }$, with $\beta \sim 0.5$ or so. The differential rotation profile becomes more cylindrical for faster rotation, even though the thermal wind is strengthening, but not enough to compensate the increased spin rates.
- Bessolaz and Brun (2011a) have looked at the influence of the aspect ratio on turbulent convection and resulting mean flows in a young star, see Fig. 34 left panel. They show that larger aspect ratio yield more solar-like differential rotation (e.g., prograde equator/slow poles), such that earlier in the PMS phase the star is most certainly prograde but that state could change as the surface convective envelope shrinks for more massive F-stars as they arrive on the ZAMS.
- Bessolaz and Brun (2011b) further simulated the young star BPtau, a $0.7 \, M_{\odot }$ star rotating about 4 times the solar rate see Fig. 34 right panel. They showed that in order to have a weak dipole as observed by spectropolarimetric techniques one must choose carefully the set of fluid parameters.

During the fully convective phase of PMS stars, dynamo action is building intense magnetic fields. As the radiative interior grows from non-existent to about 70% of the star in the case of the Sun, what is left from dynamo action in that stably stratified core remains unclear. Moss (2003) argues that more massive stars tend to conserve their fossil field more easily than later type stars for which turbulent convection motions have more time to tangle the field to small scales, hence speeding up their Ohmic diffusive decay. More recent work by Arlt (2014) and Emeriau-Viard and Brun (2017) indicate that mixed poloidal/toroidal field may survive this major structural evolution of the star.

### Solar-like stars

**Fig. 35:**
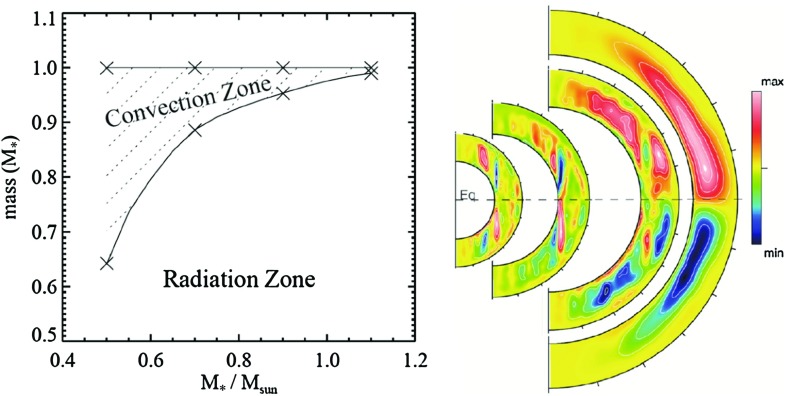
Models of solar-like stars: *Left* 1-D stellar model computed with the CESAM code (Morel 1997) showing the mass contained in the convective envelope of solar-like stars versus stellar mass, computed for 4 mass bins: 0.5, 0.7, 0.9 and 1.1 $M_{\odot }$. *Right* *Color contours* of the meridional streamfunction achieved in stellar convection models of G-K stars rotating at the solar rate realized with the ASH code. The images have been scaled to take into account the relative stellar radius difference between a G0 and K7 star. *Red tones* correspond to counter-clockwise circulation. Image reproduced by permission from Matt et al. (2011), copyright by Wiley

All solar-like stars possess a convective envelope whose thickness and mass content varies significantly. In F-stars ranging from 1.1 to 1.5 $M_{\odot }$ it is very shallow and contains very little mass (less than 1%). In K and early M dwarfs ranging from 0.9 to 0.5 $M_{\odot }$ it is deep and contains a more significant fraction of the stellar mass (cf. Fig. 35, left panel). Solar analogues ranging from 0.9 to 1.1 $M_{\odot }$ have extended convective envelopes but these usually contains little mass, of the order of a few %.^4^

This large variation of mass content and aspect ratio has direct consequences for heat and angular momentum transport in the convective envelope of solar-like stars, as shown for instance for the meridional circulation realized in 4 different modelled stars in Fig. 35 (right panel). We note that the latitudinal extent and the number of circulation cells per hemisphere vary significantly from one model to another (see also Featherstone and Miesch 2015). As we will now see this is due primarily to the relative influence of the Coriolis force on the convective flow.

As noted earlier in this review, a straightforward way to appreciate quantitatively this difference is to use Mixing Length Theory (Böhm-Vitense 1958), which states that the convective velocity in stellar envelope is proportional to the cubic root of the stellar luminosity $L_*$: $v_{\mathrm{conv}} \propto \root 3 \of {\frac{L_*}{\bar{\rho }_{cz} R_*^2}}$, with $\bar{\rho }_{cz}$ an averaged density and $R_*$ the stellar radius. We know from classical stellar evolution (Kippenhahn et al. 2013) that stellar luminosity, radius and mean density vary with stellar mass such that: $L_* \sim M_*^4$, $R_* \sim M_*^{0.9}$ and $\bar{\rho }_{cz} \sim M_*^m$, with $m<0$ (since the convection zone becomes shallower and shallower as the mass of the star increases from 0.5 to 1.5 $M_{\odot }$ leading to a strong decrease of the averaged density in the stellar convection zone). Given how these 3 quantities vary with stellar mass, convective flows are more vigorous (by at least a factor of 10) in an F-star than in M-dwarfs. This has direct consequences on the internal dynamics of solar-like stars as the influence of rotation on angular momentum and heat redistribution within the convective envelope will be different. This can be easily assessed by computing the fluid Rossby number $R_{of} \sim v_{\mathrm{conv}}/2{\varOmega }_* R_*$ (see Brun et al. 2017, for a detailed discussion). For values greater than 1, one expects the rotational influence to be weak (as in the G0 star case) whereas for small value of $R_{of}$ (for the 3 other cases shown) it is expected to be strong.

**Fig. 36:**
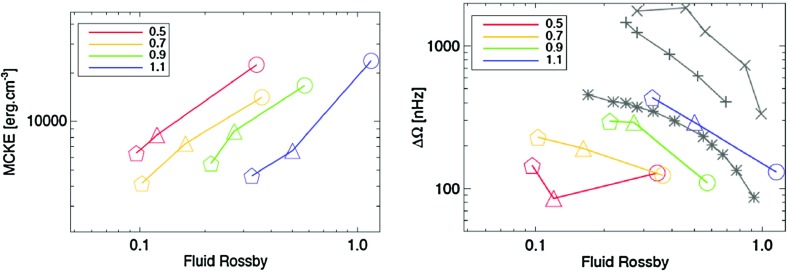
Trends of the energy in meridional circulation (MCKE; *left panel*) and of latitudinal differential rotation contrast ${\varDelta } {\varOmega }$ (*right panel*) with respect to the fluid Rossby number (Brun et al. 2015b, 2017). Models with 0.5, 0.7, 09 and 1.1 $M_{\odot }$ come from Brun et al. (2017) (*colors* as indicated in figure), 1.0 $M_{\odot }$ (*star symbols*) from Brown et al. (2008), 1.2 $M_{\odot }$ (*plus symbols*) and 1.3 $M_{\odot }$ ($\times$ *symbols*) from Augustson et al. (2012)

As discussed in Sect. 4.3, there are several observational trends that one could attempt to recover with numerical simulations of solar-like stars assuming these are robust enough:

- larger latitudinal surface differential rotation contrast for F stars than K dwarfs
- a magnetic cycle period that depends on rotation rate (though the exact nature of this dependence is still debated)
- as in young stars, the faster solar type stars rotate the more intense and toroidal are their magnetic fields

We now discuss if numerical simulations have recovered such trends and how they can help us explaining them.

Using mean field dynamo models, Jouve et al. (2010) have shown that a wide class of current solar dynamo models, e.g., based on the Babcock–Leighton type, have different behavior depending on the choice of meridional circulation profiles as well as on how its amplitude is assumed to depend on stellar rotation rate. The main reason is linked to the large dependence on meridional flow velocity in such models, e.g., $P_{\mathrm{cyc}} \propto v_{mc}^{-0.9}$ (Dikpati and Charbonneau 1999). So unless one invokes multi-cellular flows, turbulent pumping or enhanced magnetic diffusion (Guerrero and de Gouveia Dal Pino 2008; Yeates et al. 2008; Do Cao and Brun 2011; Hazra et al. 2014), such models tend to have a longer magnetic cycle period since all 3-D convection simulations (Ballot et al. 2007; Brown et al. 2008; Augustson et al. 2012; Brun et al. 2017) show that $v_{mc} \sim {\varOmega }_*^{-0.45}$ as illustrated in the left panel of Fig. 36. In other words, as stars spin faster they tend to have slower meridional flows, and hence a longer magnetic cycle period. One hence needs to short-circuit the advection path to reconcile theory with the trends deduced for instance in the HK observational survey. Recent analysis are starting to question the existence of two distinct branches (Reinhold et al. 2017).

**Fig. 37:**
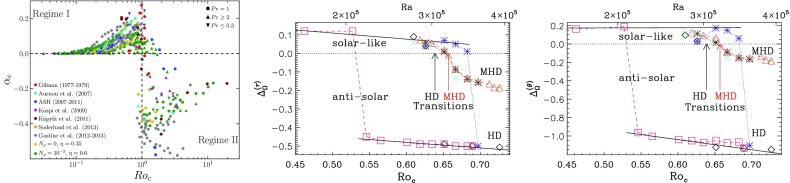
Solar-like—Antisolar-like differential rotation transition in 3-D numerical simulations of rotating global convection. *Left* image reproduced by permission from Gastine et al. (2014), copyright by the authors, showing a transition value for the Rossby number around 1.0. *Right* image reproduced by permission from Karak et al. (2015), copyright by ESO, distinguishing purely hydrodynamic simulations from dynamo ones and showing the radial and latitudinal angular contrast. We note the influence of dynamo action (MHD vs HD simulations) on the angular velocity amplitude and profile and some hysteresis shifting the transition value of the Rossby number (see also Varela et al. 2016; Fan and Fang 2014)

As of today, there are too few 3-D nonlinear dynamo simulations (see below) that possess a regular magnetic cycle to be able to assess the sensitivity to parameter change of the cycle period. There is, however, evidence that the large scale unicellular meridional circulation often assumed in conceptual Solar dynamo models is unlikely to carry over to other solar-like stars, since 3-D global stellar convective models often exhibit many meridional circulation cells per hemisphere (cf. Fig. 35; except for slowly rotating stellar models with large Rossby number). Further, as seen in previous sections and inverted by local helioseismic methods (Haber et al. 2002; Zhao et al. 2013), multiple meridional circulation cells may also occur in the the Sun, hence such complex meridional profiles are not unexpected.

Another important trend to explain for stars is their differential rotation profile (internal and surface) and how it varies with spectral types. As discussed in Sect. 5, we know that stellar rotating convection zones will yield non uniform rotation profiles. But what states do they settle into?

Several authors have recently worked on this question through high performance 3-D numercial simulations of global convection in a spherical domain (see, e.g., Ballot et al. 2007; Brown et al. 2008; Matt et al. 2011; Kimura et al. 2011; Käpylä et al. 2011; Augustson et al. 2012; Guerrero et al. 2013; Gastine et al. 2014; Käpylä et al. 2014; Karak et al. 2015; Simitev et al. 2015; Mabuchi et al. 2015; Brun et al. 2017, and references therein).^5^ They all show that the differential rotation profile is directly linked to the effective Rossby number of the simulation (see also the pioneering work by Gilman and Glatzmaier 1981). We show in Fig. 37 a recent study that summarises the most recent numerical simulations of global stellar convection. We see that anti-solar differential rotation state (slow equator, fast poles) occur at large effective Rossby number whereas solar-like differential rotation state (fast equator, slow poles) occurs at low effective Rossby number. Broadly, one might say that in the slowly-rotating limit the convection tends towards having specific angular momentum that is nearly conserved by fluid parcels as they rise or fall, leading to “anti-solar” flows; that is, a parcel moving out from the rotation axis tends to slow down if it conserves angular momentum. As the rotation rate is increased, though, systematic torques are set up within the fluid that can lead to outward/equatorward deposition of angular momentum.

**Fig. 38:**
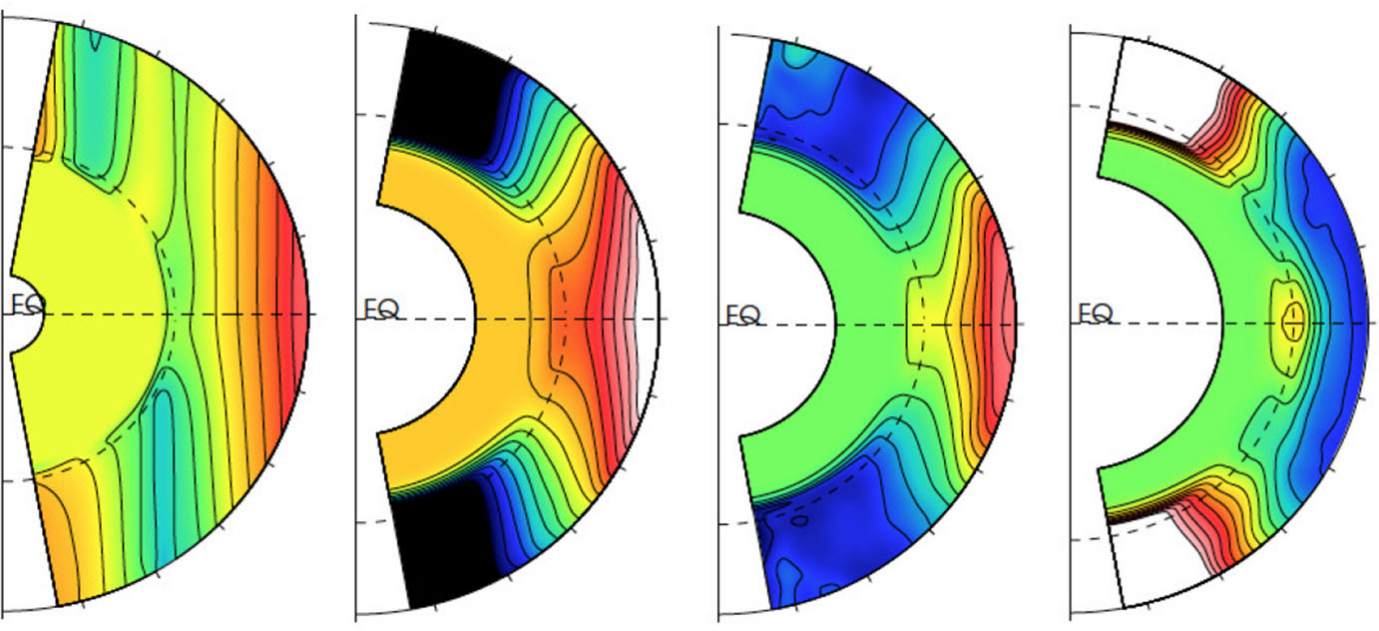
Differential rotation profile for various aspect ratio and rotation rate (Rossby number) (Brun et al. 2015b, 2017). Three stellar masses and three rotation rates are being shown. From *left* to *right*: *Color contours* showing ${\varOmega }(r,\theta )$ for a 0.5 $M_{\odot }$ star rotating at 5 ${\varOmega }_{\odot }$, 0.9 $M_{\odot }$ star rotating at 3 and 1 ${\varOmega }_{\odot }$ and 1.1 $M_{\odot }$ star rotating at ${\varOmega }_{\odot }$. Prograde rotation is shown in *red/white colors*. Image reproduced by permission from Brun et al. (2015b), copyright by Springer

In the fast rotation regime the differential rotation does not retain its monotonicity, but develops a banded profile, with alternating prograde and retrograde zonal jets as in Jupiter and Saturn (Gastine et al. 2013b; Brun et al. 2015b, 2017). We illustrate in Fig. 38 these various rotation profiles: banded and prograde, solar-like and anti-solar like.

As discussed in Sect. 4, observations seem to indicate that the amplitude of stellar differential rotation is more sensitive to the stellar spectral type than to the rotation rate (Barnes et al. 2005; Balona and Abedigamba 2016). Larger differential rotation contrasts are found in F-type stars compared to M-type stars. Both these global trends are recovered in numerical simulations as can be seen in Fig. 36 (right panel) and discussed in detail in Brun et al. (2017). In the figure, we see that for fixed Rossby number, more massive stars have a larger latitudinal differential rotation contrast ${\varDelta } {\varOmega }$. We also see that for smaller Rossby number (hence larger rotation rate), ${\varDelta } {\varOmega }$ is larger. Here depending on the observational studies considered (see Sect. 4.3), the theoretical trend is in good quantitative agreement (Saar 2011) or the variation (exponent *n*) found too large (Barnes et al. 2005). Qualitatively, MHD simulations show less variation than hydrodynamic cases versus stellar rotation, and in this sense are closer to the observational data (Karak et al. 2015; Guerrero et al. 2016) and Varela et al. (2016). This comes about from the feedback from the Lorentz force on the mean flow. Maxwell stresses tend to inhibit efficient angular momentum transport by Reynolds stresses yielding a weaker ${\varDelta } {\varOmega }$ (see discussion in Brun 2004; Fan and Fang 2014).

**Fig. 39:**
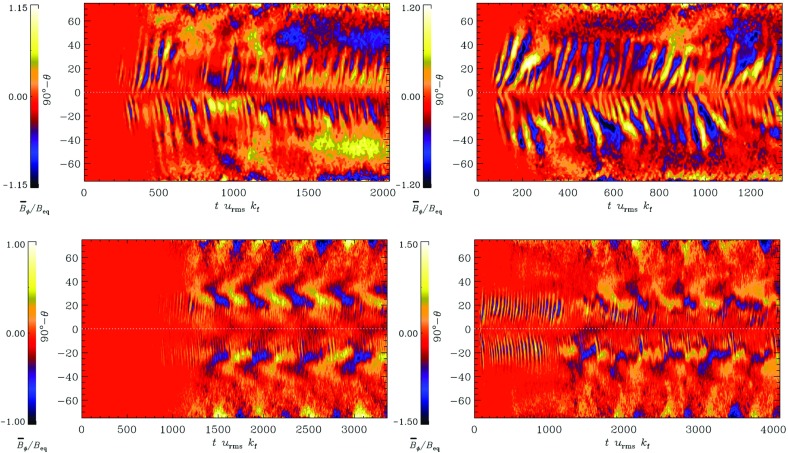
Butterfly diagram (time—latitude plot) of toroidal magnetic field in various dynamo simulation of solar-like stars. We note the change of the dynamo wave direction, from poleward at low stratification values (*top row*) to equatorward for stratified models (*bottom row*, ${\varDelta }_r \rho > 30$). Image reproduced by permission from Käpylä et al. (2013), copyright by AAS

By further exploring the parameter space, several recent studies (Käpylä et al. 2013; Nelson et al. 2013; Augustson et al. 2015; Simitev et al. 2015) have shown that low Rossby number dynamo solutions often possess interesting cyclic behavior. In Fig. 39 we show various realisations of cyclic 3-D dynamo solutions (Käpylä et al. 2013). We note that some solutions possess a poleward dynamo branch while other possess solar-like equatorward dynamo branch at mid to low latitudes. In their study Käpylä et al. (2013) advocate the role of stratification to get the latitudinal sense of propagation of the dynamo wave to change to the opposite latitudinal direction. They indicate that the higher stratification shifts the location of $\omega$ and $\alpha$ effects in the convective envelope favoring their spatio-temporal phasing and yielding more realistic cyclic equatorward propagating dynamo. In Warnecke et al. (2014) they further analyze the solution and find that Parker–Yoshimura rule holds in these cases (Parker 1955a; Yoshimura 1975). However, it is worth noting that all these simulations have been computed with a magnetic Prandtl number *Pm* of order 1. See also Käpylä et al. (2017) for recent dynamo simulations performed with various diffusivity ratios.

**Fig. 40:**
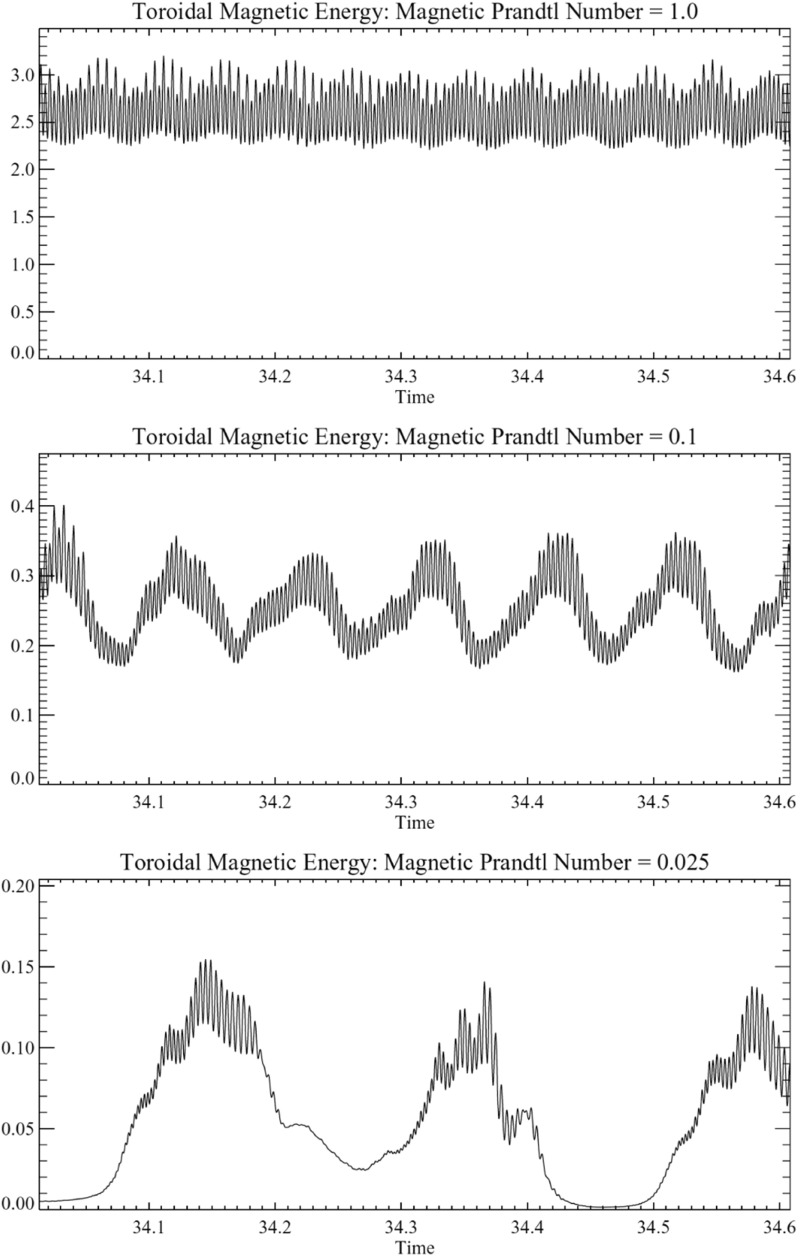
Various dynamo states as a function of the magnetic Prandlt number *Pm*. From *top* to *bottom*: $Pm=1.0, 0.1, 0.025$. We clearly see the chaotic modulation of the 11-year cycle as *Pm* is lowered. Image reproduced by permission from Bushby (2006), copyright by the authors

**Fig. 41:**
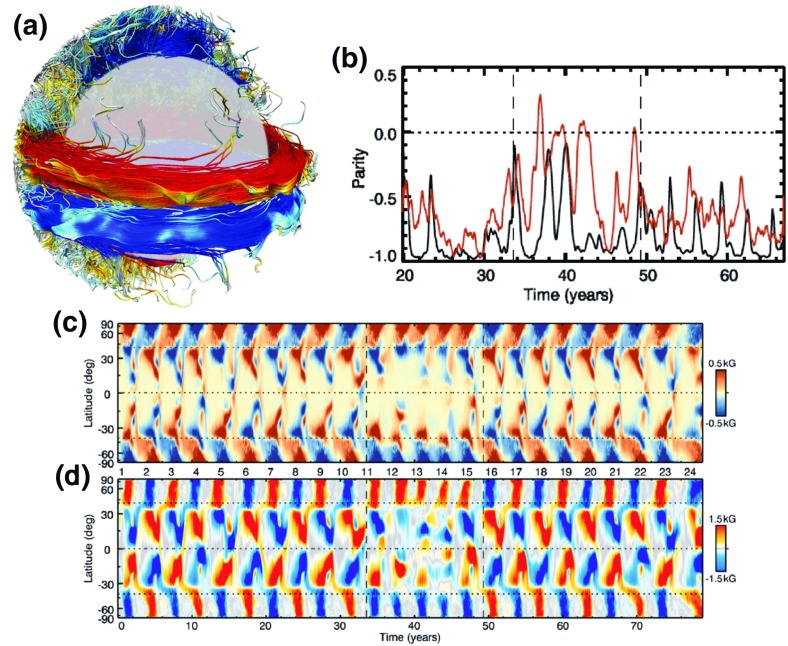
ASH simulations of a cyclic dynamo for a solar-like star rotating at 3 times the solar rate (Augustson et al. 2015). *Top left* 3-D rendering of the toroidal magnetic field displaying two magnetic wreaths of opposite polarity. *Top right* evolution of the magnetic parity (symmetric vs anti-symmetric state with respect to the equator at two depths (0.96 $R_*$ *black curve*, 0.75 $R_*$ *orange curve*)) during the period of grand minima, showing that the symmetric (quadrupolar-like) modes dominate as was observed in the Sun during the Maunder minima (Ribes and Nesme-Ribes 1993; Sokoloff and Nesme-Ribes 1994; Tobias 1998). *Bottom* Butterfly-like diagram (of respectively the radial magnetic field $B_r$ (*top panel*) and longitudinal magnetic field $B_{\phi }$ (*bottom panel*)) over 24 cycles showing a period of much lower activity akin to a grand minima. Image reproduced by permission from Augustson et al. (2015), copyright by AAS

It is well known in the dynamo community that *Pm* is another important parameter to study (see, e.g., Schekochihin et al. 2004, 2005; Ponty et al. 2005; Iskakov et al. 2007), particularly in stellar convective envelopes where it is often very small (of order $10^{-3} \text{ to } 10^{-6}$). We can gain insights by turning to nonlinear mean field dynamo studies such as those of Tobias (1997), Moss and Brooke (2000), Bushby (2006). These mean field dynamo models take into account the retroaction by the large-scale Lorentz force (also known as the Malkus–Proctor effect) and have shown that low magnetic Prandtl dynamo models ($Pm < 0.025$) yields interesting intermittent/chaotic states akin to grand minima period (see also recent work by Weiss and Tobias (2016)). This is due to the various magnetic, velocity and diffusive time scales that leads to a highly time dependent behavior when there are far apart as it is the case for low *Pm* number. We illustrate the occurrence of such intermittent dynamo states in Fig. 40, where large chaotic modulation of the 11-year cycle are shown for the lowest value of *Pm*.

By implementing a SLD (Slope-Limited Diffusion) treatment on viscous dissipation, (Augustson et al. 2015), were able to study 3-D low *Pm* low Rossby dynamo simulations and found interesting cyclic and intermittent dynamo states. In Fig. 41 we show various representations of that solution. This solution also possesses regular cyclic dynamo action ($P_{\mathrm{cyc}}\sim 3.4$ years), equatorward propagation of the dynamo wave at mid-latitudes as in (Ghizaru et al. 2010; Racine et al. 2011; Käpylä et al. 2012, 2013; Karak et al. 2015). Further, this low *Pm* simulation also possesses an intermittent state of lower magnetic energy (reduction by a factor of 3) as illustrated in Fig. 41 right panel.

The systematic influence of a tachocline in stellar dynamo has been studied recently in (Masada et al. 2013; Guerrero et al. 2016), following the work of Browning et al. (2006). The presence of a tachocline helps organizing the magnetic field at the base of the convection zone as discussed in Sect. 6.1 above.

Overall, multi-D numerical simulations of convection and dynamo in solar-like stars have recently made tremendous progresses. Most observational trends are recovered qualitatively, if not necessarily quantitatively, and cyclic dynamo solutions are now within reach in 3-D global convection simulations. Left to future work is a full assessment of how dynamo action and the magnetic cycle period are controlled (including grand minima) as stellar parameters are changed. We have seen that the large scale mean flows vary significantly so we expect the magnetic activity to do the same; recent publications (as surveyed here) confirm that this is indeed the case.

### Low-mass stars

In comparison to the vast array of models that have attempted to capture elements of the solar dynamo, the literature on dynamos in much lower-mass stars (or brown dwarfs) is rather limited. In the past few years, there has been a growing awareness that dynamos in these objects may have more in common with those in gaseous planets than with dynamos in (say) upper-main-sequence stars. Below, we briefly review both the sparse literature on low-mass stars specifically, and note some of the most significant parallels with ongoing work in the planetary dynamo community.

The first global-scale simulations of stratified convection (as opposed to mean-field models) to specifically target a fully-convective low-mass star were by Dobler et al. (2006). Their models considered fully convective spheres using a Cartesian grid-based finite-difference code (the Pencil code, used widely for other problems in astrophysical MHD). Their simulations exhibited “antisolar” differential rotation, with the equator rotating slower than the poles; the equilibrated field strength of the dynamo was of order equipartition with flows near the surface. The fields contained structure over a range of spatial scales, with the largest-scale field seemingly quite dominant. The models were only weakly stratified, with the central density a factor of about three greater than that at the photosphere, and the influence of rotation (as quantified by the Rossby number) was comparatively mild.

Later, Browning (2008) conducted anelastic simulations of the interior of a 0.3-solar mass M-dwarf (i.e., adopting an initial 1-D stratification consistent with such a star). These models included a stronger density stratification (with the surface density about a hundredth that in the interior), and considered a range of different turbulent diffusivities and resolutions, effectively spanning models that ranged from very laminar convection to reasonably complex flows (with Reynolds numbers based on the large-scale flows of order at most a few hundred). The resulting dynamo-generated fields typically attained strengths of order equipartition (relative to the rotating frame), in this case implying fields of a few kG strength. Differential rotation was established in hydrodynamic cases (with a solar-like pattern of fast equator and slow poles) but wiped out in the MHD ones, mainly as a result of strong Maxwell stresses exerted by the magnetism. The spatial structure of the magnetism was fairly complex, exhibiting structure over a broad range of scales; the mean fields (referring in this case to a longitudinal average) accounted for up to about 20% of the magnetic energy in some cases, but the majority of the field energy was on smaller scales. We attributed several elements of the simulations to the strong influence of rotation on the dynamics: the rotation rate considered in these models (equal to the solar angular velocity) implied Rossby numbers far below unity throughout most of the stellar interior, implying that rotation played a much stronger role than in typical “solar” simulations (which typically had $Ro \sim 1\text{- }0.1$).

Most recently, Yadav et al. (2015a) presented calculations that reached somewhat lower values of the relevant diffusivities than in Browning (2008), and encompassed even stronger density stratifications. Thus, for example, rotation is even more important in their calculations (relative to viscosity) than in earlier ones, as quantified by the comparatively low values of Ekman number attained; they also reached higher values of the magnetic Reynolds number (*Rm*) in portions of the computational domain than in prior works. (Radiative diffusion—i.e., conduction—is still responsible for carrying a large fraction of the heat flux in their simulations, though.) The resulting dynamo-generated field consisted of a stable axisymmetric dipole, coexisting with smaller-scale magnetism near the surface. Intriguingly, Yadav et al. also constructed synthetic observational data from their simulations and analyzed these using the Zeeman Doppler Imaging technique (as described in Sect. 4). They find that the resulting ZDI “image” consistently shows strong polar spots, and little or no toroidal magnetism; overall, the strength and morphology of the magnetism are similar to what is inferred observationally on some fully convective stars.

**Fig. 42:**
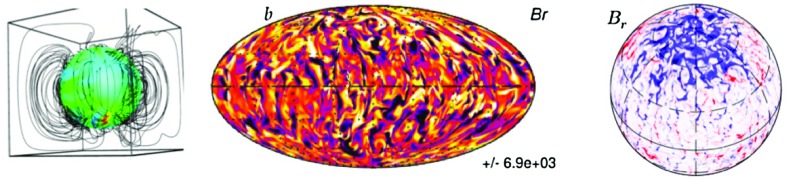
A sampling of results from global-scale 3D simulations of convection in fully convective low-mass stars. **a** Field line renderings in the models of Dobler et al. (2006). **b** Radial field sampled near the stellar surface in one of the simulations of Browning (2008), shown in Mollweide projection. **c** Radial field near the surface in a simulation of Yadav et al. (2015b). Images reproduced by permission, copyright by AAS

These three distinct sets of global simulations are sampled in Fig. 42, which displays a fieldline rendering from Dobler et al. (2006) at left, the radial field on a particular spherical shell from Browning (2008) (center), and (right) the radial field near the surface in the calculations of Yadav et al. (2015a).

Finally, as a complement to these global-scale simulations, Weber and Browning (2016) have recently conducted “thin flux tube” simulations of fully convective stars, examining the evolution of fibril fields that are presumed (in their model) to be produced in the interior by dynamo action. Their simulations use methods borrowed from the large body of work in the Solar context that has employed this approximation—see, e.g., Sect. 5.5 of this review and Fan (2009), Weber et al. (2011), and references therein, for a description of the method, its limitations, and application to the Sun. They find that in the absence of strong interior differential rotation, such fields tend to emerge near the stellar poles, unless they are generated very near the stellar surface. (If strong enough internal shear is present, the rising flux tubes can instead emerge closer to the equator.) Thus, the polar spots inferred at the surface of M-dwarfs might conceivably arise either from global-scale dipole fields that locally diminish convective heat transport (as in Yadav et al. 2015b), or from a collection of smaller-scale flux tubes that are built in the interior and emerge (as in Weber and Browning 2016), or a combination of both.

**Fig. 43:**
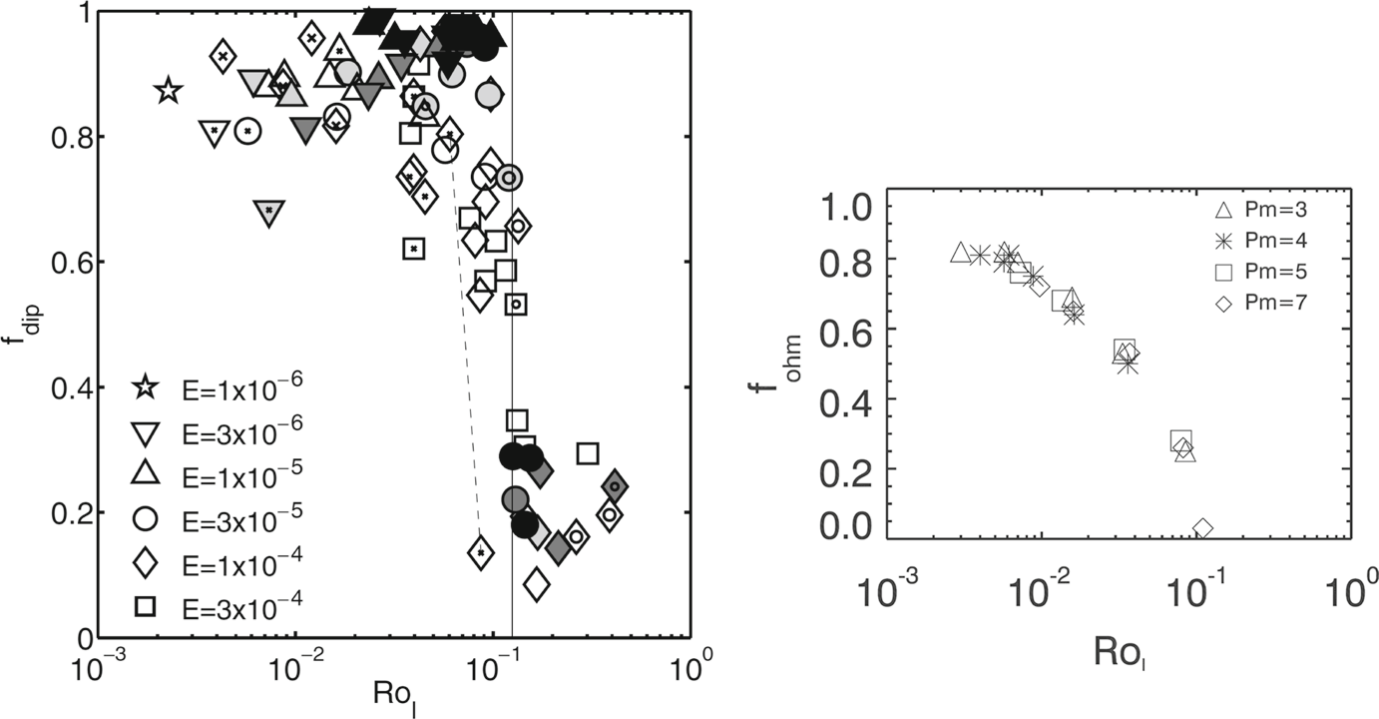
*Left* “Dipole fraction” of the magnetic field (see text) as a function of rotational influence, in Boussinesq simulations of Christensen and Aubert (2006). *Right* fraction of power dissipated Ohmically, as a function of rotational influence, in a sample of Boussinesq simulations (Schrinner 2013). Images reproduced by permission, copyright by the authors

#### Parallels with planetary dynamo simulations

In many respects, dynamo action in fully convective stars is probably as akin to what occurs in gaseous planets as it is to dynamo action in more massive (Solar-like) stars. The geometry, the strong role of rotation, and the relatively leisurely convective flows all resemble the planetary regime as much as the Solar one. These parallels only go so far: for example, a typical early M-dwarf is still an extremely good conductor throughout its interior (i.e., the magnetic Reynolds number is high almost everywhere), whereas in many planetary contexts the values of *Rm* attained may be more modest (see, e.g., Grote and Busse (2001), Sasaki et al. (2011), Roberts and King (2013) in regards to the geodynamo; Gastine et al. (2013b) in regards to Jupiter), at least in some regions of the interior. Still, we draw here on simulations of planetary dynamos as examples of the dynamics that can occur when convection, rotation, and magnetism meet in a deep spherical domain.

One of the clearest and most compelling results concerns the influence of rapid rotation on the geometry of the dynamo-generated magnetism. In Boussinesq simulations intended to model planetary dynamos, Christensen and Aubert (2006), for example, found that the “dipole fraction” of the magnetism (defined there as the ratio of the dipole field strength to the field strength summed over degrees $l=1-12$) was a strong fraction of rotation, as quantified by a modified Rossby number. These results are sampled in the left panel of Fig. 43, with $Ro_l$ defined there as $Ro \times \frac{\bar{l_u}}{\pi }$, where $\bar{l_u}$ is the mean spherical harmonic degree of the kinetic energy spectrum. In these parameter regimes—namely, unstratified convection with fixed temperature contrast—more rapid rotation clearly leads to more dipolar fields. Similar results were reported earlier by Sreenivasan and Jones (2006), who effectively varied the influence of inertia by altering the Prandtl number (at fixed *Ra* and *Ek*). There is still no (to our view) particularly compelling theory of why the dipole fraction in these simulations scales with rotation rate in this way. Another interesting aspect of the problem was highlighted by Schrinner (2013), who examined the ratio between Ohmic and total dissipation; the fraction going into Ohmic dissipation increases with rotation rate in his (Boussinesq) calculations, and this in turn implies a form of “rotation-activity” relationship in the models. This behavior is sampled in Fig. 43 (right panel). Finally, it is also clear that simulations in this regime (i.e., with convection distributed throughout a deep interior) can exhibit magnetic cycles in some cases—see, for example, Schrinner et al. (2012).

Yet another interesting aspect is the notion of “bistability”, in which simulations at the same parameters—but having different initial conditions—can exhibit very different final field configurations. See, for example, Morin et al. (2011), Simitev and Busse (2009), and Roberts (1988) for background theory and discussion. Some authors have argued that similar “strong-field” and “weak-field” branches might be realized in the stellar context (e.g., Gastine et al. 2013a; Kitchatinov et al. 2014), with this dichotomy giving rise to the observation that some particularly low-mass M-dwarfs seemingly exhibit different field strengths and morphologies at very similar rotation rates and masses (see Sect. 4, and Morin et al. 2008, 2010).

**Fig. 44:**
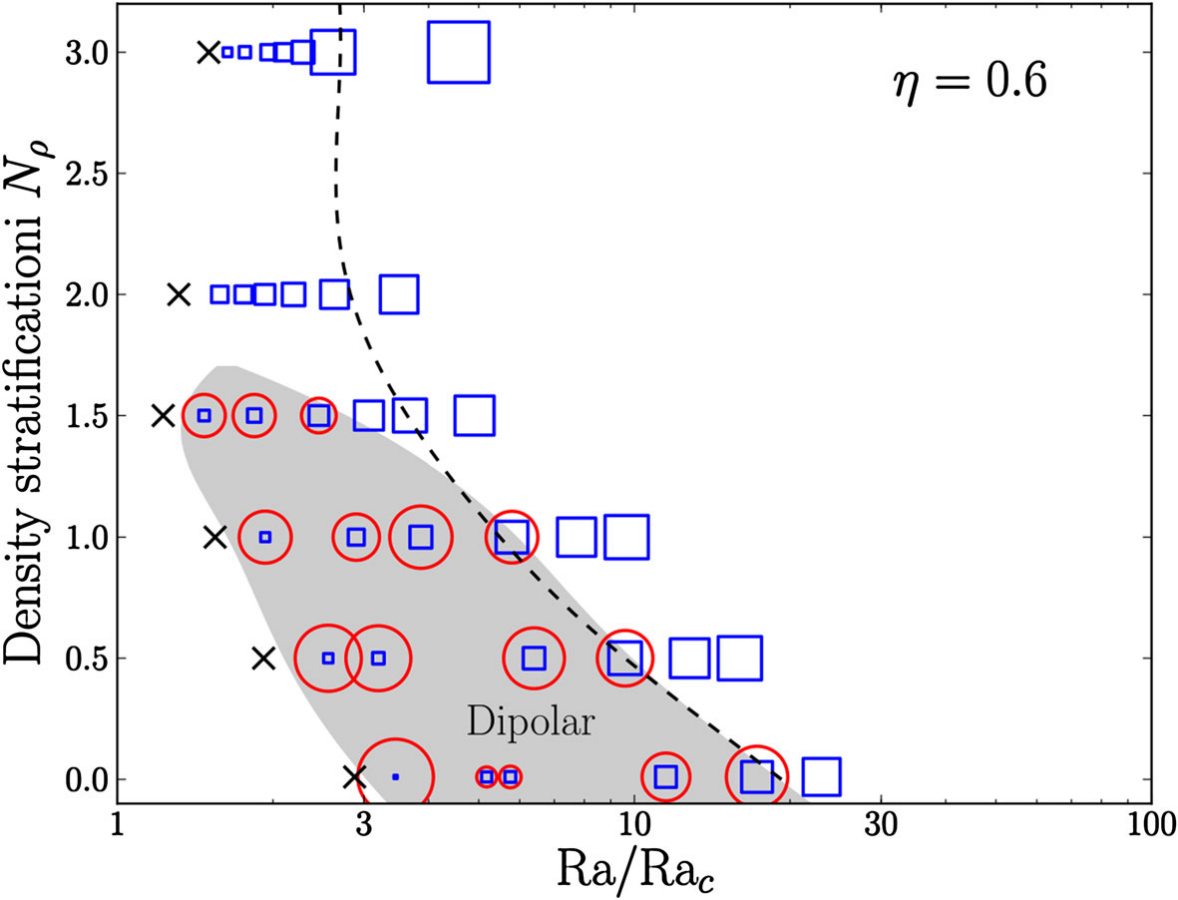
Simulations showing dipolar and multipolar morphologies, as a function of supercriticality and density stratification. *Grey region* shows regime in which predominantly dipolar solutions are found; *blue squares* correspond to multipolar dynamos, *red circles* to dipolar ones, and *black crosses* to decaying solutions. Image reproduced by permission from Gastine et al. (2012), copyright by ESO

But it is also clear that the magnetism is influenced by stratification, by the criticality and vigor of the convection, by the geometry, and by interaction with a zonal flow. An example of this is sampled in Fig. 44, taken from Gastine et al. (2012), which assesses the field morphology in a series of anelestic calculations with varying supercriticalities and density stratifications. In the parameter regime sampled here, dipolar solutions are found only at modest supercriticalities and low density stratifications; the “window” for dipolar solutions narrows as either the stratification or the criticality of the convection are increased. (Gastine et al. (2012) argue, though, that at the much lower Ekman numbers that prevail in astrophysical objects, dipolar solutions might well be possible even at high supercriticalities and in the presence of strong density stratifications—as later demonstrated to some extent in the simulations of Yadav et al. (2015a).) Other papers have examined the influence of the mass distribution (e.g., Raynaud et al. 2014), the geometry (e.g., Goudard and Dormy 2008), and the the zonal flow (see, e.g., Duarte et al. 2013) in planetary contexts.

Clearly, many different effects conspire to influence the magnetism: strong rotation helps build ordered (dipolar) fields; strong stratification, or high levels of turbulence (as encapsulated in various ways by *Re*, *Rm*, and *Ra*) can counteract this to some extent. How these combine in the asymptotically high-*Ra*, high-*Re* regime is not totally clear; perhaps more troublingly, it is not always clear even which numerical simulations (among those that are tractable today) most accurately probe this regime. As one example, it is currently possible to conduct calculations at high *Rm*, or to run calculations in which rotation dominates over inertial forces, but it is difficult to do both at the same time. (Because the Rossby number *Ro* is the ratio of inertial to Coriolis terms, and the Reynolds number is the ratio of inertial to viscous terms, it is computationally easier to address the low-*Ro* limit—rapid rotation, relative to inertia—if the Reynolds number is also low, and vice versa.) For partly historical reasons, many “stellar” simulations have chosen to push towards the high-*Ra*, high-*Re* regime, but this then usually implies that rotation plays only a moderate role in the dynamics; meanwhile many “planetary” calculations have pushed to much lower *Ro*, while usually considering flows that are closer to the onset of convection (i.e., lower $Ra/Ra_c$) and less turbulent (lower *Re*, *Rm*, etc). But simulations are now starting to bridge the gap between these different regimes, and we believe that the most significant findings noted here—including the strong role of rotation, mediated by stratification and turbulence—are likely to be robust.

### More massive stars

Stars of more than about 1.2 solar masses have convective cores, overlying stable shells, and in some cases thin near-surface convection zones as well. In this section we discuss some aspects of the *core convection* occurring in these stars, and the dynamics of the thick stable layer above it, relegating most analysis of convection occurring in the *envelopes* of such stars to Sect. 6.3. (The envelope convection is, after all, solar-like in geometry, even if dissimilar in some other respects.)

These regions of core convection rotate, and are hot enough to be excellent conductors, so a generic expectation is that they will also act as dynamos. (Whether such dynamos might have any observable impact at the surface is another matter, as discussed below.) Here we highlight some features of the convective flows in such objects, and the resulting magnetism, as revealed by simulations. We also briefly summarize simulations dealing with some aspects of the overlying stable region: namely, the relaxation of “fossil” fields there, possible dynamo action associated with various MHD instabilities, and the transport of momentum and energy by waves.

#### Core convection simulations: aspects of flows and fields

Early 3-D simulations of (hydrodynamic) core convection in massive stars were presented in Kuhlen et al. (2003), using a version of Glatzmaier’s anelastic code, and by Browning et al. (2004) using a version of the ASH code (previously applied to Solar-like stars, as discussed above). Earlier 2-D simulations were conducted by Deupree (2000), and mean-field dynamo models were calculated by Charbonneau and MacGregor (2001). The first 3-D simulations incorporating magnetism were published in Brun et al. (2005). Later 3-D models, including some aspects of the interaction between core convection dynamos and a pre-existing stable magnetic field within the radiative layer, were conducted by Featherstone et al. (2009). Dynamo action in the cores of more massive B-type stars has recently been examined in the anelastic 3-D simulations of Augustson et al. (2016). Meakin and Arnett (2007) have studied core convection in an interior “wedge” of an even more massive (23 solar-mass) star, and Gilet et al. (2013) have studied massive star core convection using the low-Mach number Maestro code. Other authors have also considered 1-D models of massive stars to follow the buoyant rise of magnetic structures (based on the thin flux tube approximation, see Sect. 5.5 and Spruit 1981) from the edge of the convective core up to the stellar surface MacGregor and Cassinelli (2003), MacDonald and Mullan (2004). The latter found that the magnetic structures are likely to rise only very slowly towards the surface, making it unlikely that the observed surface fields of Ap/Bp stars could result from the core dynamo.

The core convective flows are strikingly different from those realized in the near-surface convection zone of Solar-like stars (Sect. 6.1) or in low-mass stars (Sect. 6.3). On the one hand, the flow is quite vigorous: simulations suggest that typical flow speeds for large-scale convective flows might approach, for example, 50 m s$^{-1}$ in the convective cores of A-type stars (e.g., Browning et al. 2004), 100 m s$^{-1}$ in more massive B-stars (Augustson et al. 2016), and about $10^{3}$ m s$^{-1}$ in the simulations (of a 15 solar-mass star) in Gilet et al. (2013). These values are broadly consistent with the simple MLT estimate that velocities should scale as $v \propto (F{/}\rho )^{1/3}$: in the center of a massive star, the flux is high (relative to the Sun or an M-dwarf) and, perhaps less intuitively, the density is fairly low (compared to the center of a lower-mass star). (The central density of a B-type star is about 20 g cm$^{-3}$, whereas that in an M-dwarf is roughly sixfold higher.) These rapid velocities suggest that in many cases the flows are only weakly influenced by rotation, since the Rossby number is then greater than unity except in objects rotating at at least a few percent of the breakup velocity. On the other hand, the flows are larger-scale (both relative to the size of the system and in absolute terms) than in near-surface convection, with the convection here appearing as broad upflows and downflows of low spherical harmonic degree. This is in keeping with the large density scale heights that prevail in these regions; further, there is little evident asymmetry between upflows and downflows (again in sharp contrast to what is observed at the surface of the Sun), since neither strong density stratification nor radiative cooling effects (which can lead to narrower downflows) are present.

The flows can transport angular momentum as well as heat, and in some cases this this leads to pronounced differential rotation. Browning et al. (2004), for example, showed that the convective cores tended to have angular velocity increasing outwards in simulations (of A-type stars) with a strong rotational influence, whereas slower rotators had differential rotation of the opposite sign. Similar results were reported by Augustson et al. (2016) in the context of B-star magnetism. Broadly, these findings are consistent with trends found in simulations of solar-like stars by Gilman and collaborators in the 1970s and 1980s (see Sect. 6.1), as well as a variety of more recent simulations summarized by Gastine et al. (2014).

The core convective motions do not, in general, stop precisely at the point where the entropy stratification becomes stable. They possess momentum, and so can continue into what, in a progenitor 1-D structure model, would have been stably stratified, establishing regions of penetration and overshooting (see Zahn 1991; Brummell et al. 2002). The extent of the overshooting, and the extent to which it modifies the background stratification, typically depends on a variety of factors including the filling factor of overshooting flows and their Peclet number. In most semi-analytical models, the extent of the overshooting region is typically taken to be a fraction of a pressure scale height at the core boundary; broadly, we would summarize the simulations as being consistent with this, but many uncertainties (about the stratification within this region, its overall extent, and its dependence on latitude) remain. Surprisingly few simulations have addressed the problem of overshooting from convective cores specifically (i.e., overshooting against the direction of gravity and in the direction of decreasing density): see, e.g., the early work described in Roxburgh (1993), Roxburgh and Simmons (1993) and discussions in Browning et al. (2004), Viallet et al. (2015). Much more numerical work has focused on the case where the stable region underlies the convective one—see, e.g., Freytag et al. (1996), Tobias et al. (2001), Brummell et al. (2002), Rogers and Glatzmaier (2005), Rogers and MacGregor (2010), Brun et al. (2011)—or on the launching of gravity waves by such overshooting motions (as discussed below).

**Fig. 45:**
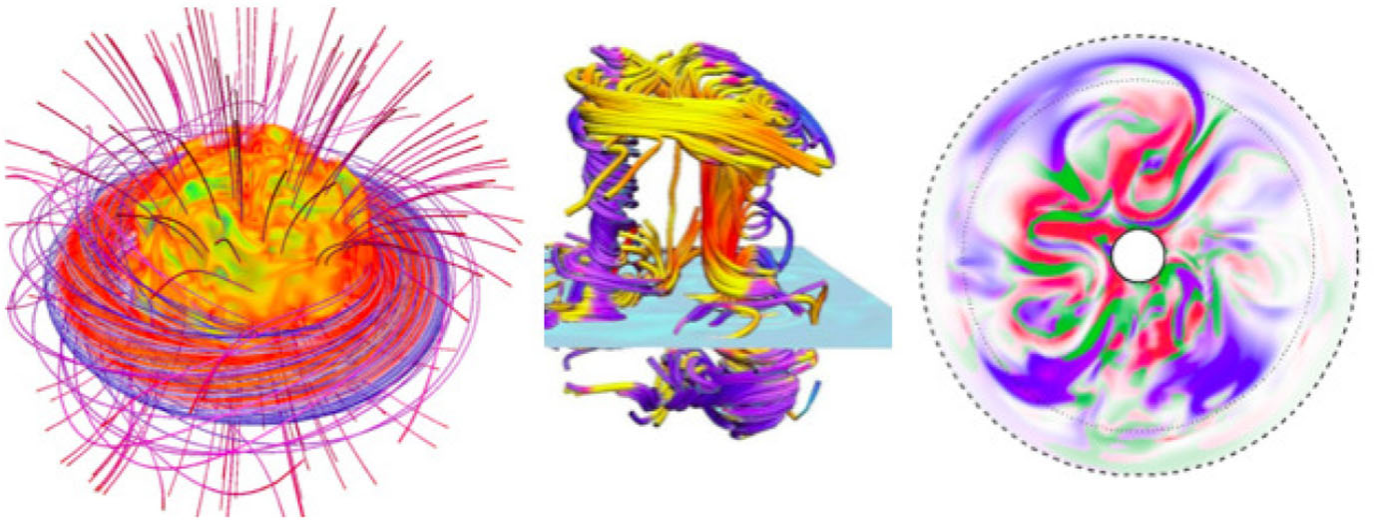
Magnetic fields and flows in a simulation of dynamo action by core convection in an A-type star. *Left* overall magnetic field line rendering, showing the toroidal field present in the radiative envelope and the accompanying poloidal field threading the convective core. *Middle* streamlines of columnar flows, with blue tones indicating northward motion and *yellow* indicating southward. *Right* kinetic energy (*red*) and magnetic energy (*blue*) in the equatorial plane at a particular instant in the simulation, viewed from the pole. Image reproduced by permission from Featherstone et al. (2009), copyright by AAS

The core convective flows readily act as magnetic dynamos. Some aspects of field structure and evolution are discussed in (Brun et al. 2005), Featherstone et al. (2009), and Augustson et al. (2016). The overall strength of the magnetism, in the parameter regimes probed in these simulations, is typically within a factor of a few of equipartition with the kinetic energy (relative to the rotating frame). This may depend on the properties of the initial field, though, and stronger fields may be possible in some instances. In Brun et al. (2005), for example, the magnetism attained energy densities comparable to the flows; but meanwhile Featherstone et al. (2009), who imposed an additional magnetic field component intended to represent the “fossil” field that might be present in the radiative envelope, the dynamo re-equilibrated to a different state in which the magnetic energy was about ten times the equipartition value. The initial “fossil” field in the Featherstone et al. (2009) simulations represented only a relatively small perturbation to the magnetic * energy* of the core dynamo, but was a large perturbation to the *flux* produced by that dynamo. Some of the flows and fields produced in the calculations of Featherstone et al. (2009) are sampled in Fig. 45. In particular, we note that the large scale nature of the convective flows, and the zonal flows that are present in some parameter regimes, can combine to yield magnetism with substantial “large”-scale components; here, though, the largest scales possible are similar to the scale of individual convective eddies, so in that sense the system might be regarded as a kind of small-scale dynamo. (Its temporal behavior, for example, is typically chaotic rather than showing orderly polarity reversals or propagation.) Featherstone et al. (2009) found some dependence of the geometry of the field on the initial field geometry: simulations with an initially large-scale “fossil” field ultimately built larger-scale structures amidst the convection than those started from a small-scale seed field (Brun et al. 2005). This is somewhat reminiscent of the “strong-field branch” noted above in the context of planetary magnetism, but the correspondence is not exact and many factors probably influence the field strength ultimately achieved by the dynamo. Augustson et al. (2016), for example, find super-equipartition states achieved without the addition of any initial large-scale “fossil” component. In numerical terms, these super-equipartition states imply peak field strengths exceeding a mega-gauss in the core of a B-type star (Augustson et al. 2016). In these super-equipartition systems, the field strongly suppresses the zonal flows of differential rotation, but strong convection persists. The field survives at these strengths, without overly “quenching” the flows that generate it, partly by being spatially and temporally segregated in time from the strongest flows (see Fig. 45): i.e., the field *does* locally suppress the convective flows, but by then the strongest flows have moved on (and are building field elsewhere). It is, as one of the authors has noted, a little like a debtor staying one step ahead of his or her creditors (Juri Toomre, private communication).

#### Evolution of magnetism in stable layers

The evolution of fields and flows in the stable envelope, meanwhile, has been studied in simulations by Braithwaite and Spruit (2006) and follow-on papers, as described below; see review by Braithwaite and Spruit (2015). As discussed in Sect. 5, field evolution in such regions is in large part mediated by the action of various MHD instabilities, and by the interaction of these with the flows (e.g., differential rotation). In some cases these simply shape a pre-existing field, whereas in others it may be that dynamo action is possible. Various authors have investigated these interactions using 2-D and 3-D simulations; among them, we note the papers by Duez et al. (2010), Zahn et al. (2007), Arlt and Rüdiger (2011), and the very recent simulations of Jouve et al. (2015) and Gaurat et al. (2015).

Broadly, the evolution of the magnetism in these simulations confirms many of the analytical expectations highlighted in Sect. 5: purely toroidal or poloidal fields are unstable, and the field evolves rapidly towards a mixed poloidal–toroidal state (Braithwaite and Nordlund 2006). If differential rotation is initially present, this represents a potential source of free energy, which can in some cases be tapped to amplify an initially weak magnetic field (Spruit 2002); whether this ultimately results in self-sustaining dynamo action is still a matter of some debate. Braithwaite (2006) presented “proof of concept” simulations modeling a stably stratified Cartesian domain, intended to model a small section of a star along the rotation axis, and found dynamo action driven by the interaction between differential rotation and instabilities of the toroidal field. Zahn et al. (2007) modeled full spherical domains within the anelastic approximation, and did not find self-sustaining dynamo action; neither did Gellert et al. (2008), modeling the (cylindrical) Taylor–Couette problem. But modeling the full dynamo loop (as envisioned by Spruit 2002, and refined in later papers—see, e.g., discussions in Zahn et al. (2007), Rüdiger et al. (2016), Ibáñez-Mejía and Braithwaite (2015)) in a spherical geometry is numerically quite challenging, and it is likely that numerical effects still play a significant role in determining whether the “dynamo loop” can be closed in any given simulation. More generally, the simulations have suggested that the interaction between the different instabilities and flow fields present—including the Tayler instability as envisioned in Spruit (2002), but also (for example) the magnetorotational instability (MRI) and the magnetic buoyancy of toroidal fields—can be quite intricate. Jouve et al. (2015), for example, conducting 3D simulations in an unstratified spherical shell, have shown that in their parameter regime the MRI is always favored over the Tayler instability; in their models (as in previous work on proto-neutron stars by Masada et al. 2006) with strong differential rotation, the MRI-driven poloidal field is wound up, leading to a significant enhancement of angular momentum transport in some cases. Of course, real massive star cores do have stable stratification, and it is very likely that this will change which modes are preferred in any given instance (since radial motions are then strongly suppressed by the stratification, as noted in (Spruit 1999, 2002). Finally, though not directly intended to model massive stars, we also note that other authors have modeled the same instabilities in other contexts; e.g., Rüdiger et al. (2015) highlight the angular momentum transport achieved by the magnetic fields in a stable, cylindrical geometry subject to various differential rotation profiles.

#### Waves in the stable envelope

In the stably stratified envelopes of massive stars, buoyancy acts as a restoring force: parcels of fluid displaced upwards quickly find themselves denser and cooler than their surroundings, and so sink; in general the result is an oscillation with a frequency limited by the Brunt-Vaisalla frequency of the medium. Such *gravity waves* have been studied for decades in the context of Earth’s atmosphere (see, e.g., Plumb and McEwan 1978; Baldwin et al. 2001), and have also been extensively analyzed for their possible role in stars, whether as a means of transporting angular momentum (see, e.g., Goldreich and Nicholson 1989; Kumar and Quataert 1997; Zahn et al. 1997; Talon et al. 2002; Rogers and Glatzmaier 2006; Alvan et al. 2014) or as a source of mixing (e.g., García López and Spruit 1991). In the context of massive stars, such motions are likely to be excited both by turbulent overshooting from the core, and by shear stress from the convection. A variety of numerical simulations have attempted to gauge the properties and consequences of these waves. A good recent example is Rogers et al. (2013), who also provide a cogent summary of previous work on the subject; we here summarize only a few main points.

The overall energy in gravity waves excited by Reynolds stresses in the convection is nontrivial, and they can transport a significant amount of angular momentum. The power in waves, integrated over all frequencies and wavenumbers, is expected in classic models to be of order the luminosity carried by convection times the Mach number of the flows (see Goldreich et al. 1994; Kumar and Quataert 1997); but see (Lecoanet and Quataert 2013) for situations where the power in waves can be substantially greater than this. The wave amplitude should have some frequency dependence: in the case of excitation by Reynolds stresses, the waves ought to know something about the turnover time of typical convective eddies; if excited mostly by overshooting plumes, we might (for example) expect some spatial and temporal dependence arising from the properties of the (small) region where motions are buoyantly braked. The radiative damping of the waves is also dependent on their frequency in the local rest frame of the fluid: the damping length scales with the frequency of the wave (to the third or fourth power, depending on whether the horizontal or vertical group velocity is most relevant), so waves at high frequency have a greater dissipation length (i.e., propagate a greater distance before damping). If the waves propagate through a differentially rotating medium, they are Doppler shifted, changing their frequency and hence their damping length. The interaction between these effects can in some cases lead to the amplification of mean flows. Suppose, for example, that waves are excited at the core-envelope interface of a massive star and propagate upwards, and that the angular velocity of the medium increases somewhat with radius. In this case prograde waves will be Doppler shifted to lower frequency, whereas retrograde waves are shifted to higher frequencies; the prograde waves will then go a shorter distance than the retrograde waves. The waves deposit angular momentum where they are damped, so here positive angular momentum would be deposited near the core (by the prograde waves shifted to low frequency) and retrograde angular momentum would be deposited further out, leading to stronger shear.

**Fig. 46:**
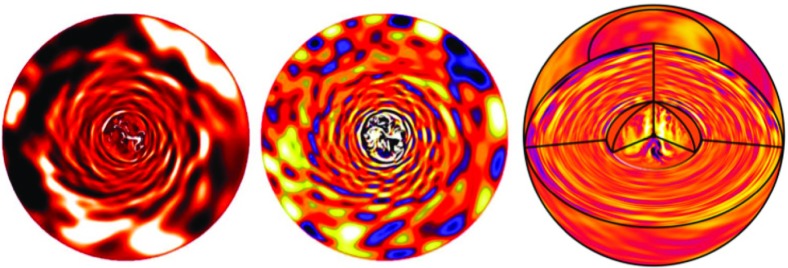
Gravity wave excitation in simulations of massive stars. Shown are temperature and vorticity perturbations (*left*, *middle*) in simulations from Rogers (2015). Image reproduced by permission, copyright by AAS. *Rightmost panel* (adapted from Augustson et al. 2016) shows volume projection of radial velocities; the *inner sphere* shows the convective core, and the *outer sphere* captures a portion of the overlying stable envelope

The establishment and strengthening of shear by the waves, and some of their possible implications for massive stars, have been studied numerically in depth by, e.g., Rogers et al. (2013), Rogers (2015), Rogers et al. (2012). Some of their results (from Rogers 2015) are sampled in Fig. 46. Though strictly applicable to waves in water, we also note the simulations of Lecoanet et al. (2015) as providing some striking numerical evidence for the validity of simple models of bulk excitation (and damping) of the waves in this regime. Wave-driven transport in massive stars has also recently been considered analytically by Fuller et al. (2014), Fuller et al. (2015), and other analytical aspects of gravity wave propagation in rotating stars have been studied by Prat et al. (2016).

#### Summary and possible implications

The simulations summarized here, together with basic theory, allow us to draw a few conclusions about the dynamics occurring in the interiors of massive stars. The cores of these stars host vigorous convection, which can act effectively as a magnetic dynamo. As a consequence, we expect that every main-sequence massive star possess interior magnetism, and in many cases the field strengths reached may be quite high. For example, a field that is within a factor of a few of equipartition (with the convection) in the core of a B-type star could reach strengths of more than $10^6$ G (as in the simulations Augustson et al. 2016). The convective flows overshoot into the surrounding stable envelope, mixing material and (together with the core convection itself) exciting gravity waves that propagate through the envelope. The complex interaction of these waves—with shear, with magnetism and rotation, and with each other—will certainly transport angular momentum and energy within the star; but the exact amplitude and spectrum of the waves, and the form of the rotation profile that ultimately arises as the end state of this interaction, remains somewhat uncertain. Magnetism within the stable layer itself is likely ruled mostly by the evolution of the MHD instabilities noted above (Tayler, MRI) that act, for example, to convert initially purely toroidal fields to mixed poloidal–toroidal ones; if there is differential rotation present, this in turn can amplify the fields so produced, and dynamo action is likely possible in some cases. This process, too, has broad implications for angular momentum transport within massive stars on and off the main sequence, and so in turn for their evolution. Several authors have, for example, employed various analytical or semi-analytical prescriptions for the angular momentum transport by a possible Tayler–Spruit dynamo, or by gravity waves, and studied the implications for, e.g., core rotation (Cantiello et al. 2014; Fuller et al. 2015; Wheeler et al. 2015) and the evolution of massive stars approaching core collapse (e.g., Quataert and Shiode 2012; Shiode and Quataert 2014).

Our ability to forecast what all this implies for observations at the stellar surface is more limited, and many uncertainties remain. The strong magnetic fields generated in the core might begin to rise buoyantly through the envelope, but (because this region is stably stratified) this rise occurs slowly, mediated by radiative heating into the rising flux tubes (MacGregor and Cassinelli 2003). Further, as noted in MacDonald and Mullan (2004), compositional gradients that are likely to be present in the star can act to slow this rise, so that it may be difficult for tubes to arrive at the surface within a main-sequence lifetime. If they did survive to the surface, it is not at all clear what form the surface field would then take, since we currently have no effective theory of how often, where, or in what multitudes such flux tubes might be produced within the core. Given these issues, and the nature of the surface magnetic trends discussed in Sect. 4, most workers have concluded that the surface fields are likely fossil fields, with no active link to the core dynamo. In this case, the surface field arises essentially as the end state of the instabilities described here (and in Sect. 4.5, and as reviewed recently in Braithwaite and Spruit 2015), interacting with rotation. (See Sect. 5.4 for an account of various possibilities.)

Similarly, the gravity waves induced at the core-envelope boundary may well have observable consequences at the surface. Some intriguing possibilities have been explored, for example, by Rogers et al. (2013) and Rogers (2015)—in particular, they argue that angular momentum transport by the waves generically leads to differential rotation between the core and envelope, and that the surface rotation rate might oscillate as a consequence of the shear-layer-oscillation established by the waves. This is turn would imply that the surface rotation rate (as measured by other methods) changes with time, and in particular does not always track the overall interior rotation rate well; in this light, some otherwise puzzling findings (e.g., the misalignment of some hot Jupiters around their host stars) may be partly a consequence of just measuring the “wrong” rotation rate (i.e., the transient signal induced by wave transport near the surface).

Finally, we note that recent asteroseismic observations have begun to probe massive star interiors as well, though in most cases these observations have dealt with somewhat more evolved stars. Stello et al. (2016), for example, find that many stars that possessed convective cores exhibit suppressed dipole acoustic modes; in light of the modeling of Fuller et al. (2015), who showed that such modes can be trapped and reflected within the interior in the presence of strong magnetism, Stello et al. suggest that powerful magnetic fields lurk in the interiors of these stars.

## Perspectives

Just how does a star like the Sun build its magnetic field? Despite decades of effort, we still do not have a complete answer to this question. But we have a number of clues—gleaned from observations of the Sun, from theory and simulation, and increasingly also from study of other stars. Below, we briefly summarize some of these clues, as reviewed in the previous sections, and close with a short sampling of open questions. As with the rest of the review, our summary is not intended to be particularly comprehensive, but merely to serve as a signpost to problems we would be delighted to see resolved in the coming years.

A few broad, qualitative conclusions are worth repetition here. In many stars, *convection* is implicated by both observations and theory as the means by which observable magnetic fields are built. This is evident, for example, in the frequency of observed magnetism in stars with convective envelopes, and the relative rarity of such fields in other (more massive) stars (see Sect. 4); in principle this could arise from an indirect dependence of field properties on the convection—for example, through the influence of differential rotation built by the flows, acting in concert with magnetic instabilities—but in many cases the direct action of the convective flows is probably crucial. From a theoretical point of view, convection itself is a prototypical example of a turbulent flow, capable in many circumstances of acting as a *magnetic dynamo* that converts kinetic energy into magnetic (as described in Sect. 5.2).

Rotation also plays a key role in the magnetism—and more general life—of many stars, as again established by both observations and theory. The existence of a strong correlation between rotation and surface magnetic activity, and likewise the slow spindown of main-sequence stars (through angular momentum loss via a magnetized stellar wind) are both facets of this; in principle, both provide powerful constraints on dynamo theory. We know from such observations that the Sun once spun more rapidly than it does today, and was more magnetically active—but its future is somewhat less certain, with different groups currently reaching different conclusions about the rate of spindown (and the behavior of magnetic activity) in older Sun-like stars. On a somewhat more detailed level, spectropolarimetry and Zeeman broadening measurements have indicated that the surface magnetic field—rather than just the chromospheric or coronal activity, for example—also respond strongly to rotation, suggesting a dynamo origin for the rotation-activity correlation.

Though convection and rotation are pivotal in many circumstances, they are of course not the only processes that can lead to observable stellar magnetism. Not all stellar magnetic fields observed today are generated by dynamo action: in the most massive stars, long-lived surface fields are most likely the remnants of fields from earlier epochs, decaying away over Gyr. (Even in these objects, though, vigorous core convection surely builds its own magnetism; the remnants of this are now arguably being probed by asteroseismology in red giants, as in the very recent work of Stello et al. 2016.) In other stars, dynamo action might arise not from convection, but from the action of MHD instabilities coupled to differential rotation, as described in Sect. 6.5; although the detailed operation of such dynamos remains a topic of debate, it seems clear that field growth in non-convective regions is possible in some circumstances.

At a more quantitative level, although the *origins* of stellar magnetism may be clear, we still do not have a particularly good (predictive) understanding of what sets its strength, morphology, or temporal properties (i.e., whether a given star has magnetic cycles, or the period of such cycles). Theory has of course provided many clues, and numerical simulations are now beginning to probe parameter regimes that are “interesting”, in the sense that phenomena other than dissipation are playing leading-order roles in the dynamics; we briefly summarize these here. Broadly, it is clear again that rotation is critical, as highlighted for example both by stellar and by *planetary* dynamo simulations (see Sect. 6.4) in which more rapid rotation tends to lead to more dipolar magnetic fields, all else being equal. (By this, we mean more than the rotation-as-symmetry-breaking mechanism that has long been present in models of mean field generation; rather, rotation in these systems seemingly organizes the flow in such a way as to allow system-scale fields.) Shear can also play a strong role, both as a direct agent of field amplification and also perhaps through its influence on other processes (like small-scale dynamo action). The strength of the fields generated by dynamo action is, as we have discussed, not easy to estimate in general; many different proposed “scaling laws” exist (see Sect. 5.2.1), with these largely reflecting different plausible balances among the different agents (rotation, buoyancy, shear, etc) that act to shape the field. It is fair to say, though, that most numerical simulations in a stellar context give equilibrated magnetic fields within a factor of a few of equipartition with the convection; stronger fields appear to be possible in systems (like planets) that rotate especially rapidly. The development of temporal variability—i.e., the presence or absence of cycles—is still not particularly well understood; however, many published simulations now exist that at least display cyclical activity (and spatial propagation) at moderately turbulent parameter regimes, and we are optimistic that a clear understanding of what delineates cyclical from steady solutions will emerge soon. Similarly, while many more detailed properties of dynamo “waves” in such simulations remain a topic of debate (see Sect. 5.3), the increasing number of cyclical specimens in the “dynamo zoo” (including some that display equatorward propagation like that observed in the Sun) must ultimately contribute to our understanding of the existence and properties of magnetic cycles on real stars. It is worth recalling, though, that such cycles may rely partly on physics that is not yet captured in (global) simulations: In the Sun in particular, it is becoming increasingly clear that “Babcock–Leighton” effects (involving the emergence and decay of tilted active regions at the surface) are linked to the reversal of the overall field. How such mechanisms may change in other stars, and how they interact with cycles arising by other, deeper-seated mechanisms, is not yet clear. Many more detailed theoretical uncertainties have of course been described here in the previous sections.

Any theory must be constrained and challenged by observations, so we close here with a short sampling of “open questions and challenges”. In this review, we have summarized a variety of results about the nature of surface magnetic activity, the mass loss of stars, their spindown over time, and how all these are linked to evolutionary state. The observational constraints in these areas are so rapidly improving, and so heroic in scope, that it may seem churlish to ask for better; but ask we will.

- Many powerful constraints are now coming from studies of stellar spindown. At some level, linking these observations to theory requires some estimate or prior knowledge of the mass loss rate, and at present these are very poorly known. The only “direct” measurements (as described in Sect. 4) come from the astrospheric method used by Wood et al., but it is worth repeating that even these still must assume a velocity for the wind. More measurements, at other evolutionary states, would be extraordinarily useful.
- Although spectropolarimetry is providing extraordinary new information, the subset of the surface magnetism being probed by the technique—and how, for example “cycles” probed by ZDI relate to cycles in the full field—is not always clear (to us, anyway). Very recent efforts to observe the Sun as a star using such techniques should prove very useful in this regard; more generally, we would be keen to see more measurements made in all four Stokes parameters, despite the obvious observational cost, since this potentially provides powerful new constraints. Similarly, long-term measurement with both spectropolarimetry and other more traditional “activity” tracers will help provide a more complete picture of the surface magnetism.
- In some models of the Solar cycle, and likewise of magnetism in other stars, the meridional circulation plays a critical role. Measurements of this flow—including, in the Solar case, whether it is single-celled or multi-celled in depth—are therefore of particular importance.
- Similarly, the differential rotation within stars plays a major role in virtually all models of dynamo action, whether convective or otherwise. Constraints on these from surface photometry and from asteroseismology have begun to appear, and are (from our point of view) one of the great triumphs of observational stellar astronomy in the last decade. We eagerly await more information about how these zonal flows change as a function of other parameters (like a star’s mass, age, or rotation rate). Indeed it will be interesting to see if future observations confirm a change of dynamo mode near the solar Rossby number.
- Measurements of cycle period as a function of other stellar parameters such as bolometric luminosity and metallicity have the potential to completely rule out, or otherwise strongly constrain, many models of the dynamo. Long-term modeling (in any and all tracers of the magnetism) is thus crucial. The advent of the Large Synoptic Survey Telescope (LSST), which will monitor the photometric variability of billions of stars for a decade, will be revolutionary in this regard; however, even this will probe only a subset of possible cycle periods, and ideally would be supplemented by even longer-term monitoring.

It is perhaps too easy in a review of this nature to be focused on what has been done, rather than what has actually been learned, or remains to be learned. It is worth repeating that all the simulations we have described here operate in parameter regimes very far removed from real stellar interiors, and it is not always clear which aspects of the simulated solutions are representative of actual stars. Clearly we are still missing a great many effects, and many details remain unclear. For example, no global-scale dynamo simulation performed to date has really captured the generation of the magnetic structures that are the precursors to surface active regions, though some recent work (described in Sects. 6.1 and 5.5) has begun to come closer. How strong, fibril fields are generated in the Solar interior, how these rise to the surface where they may be observed—and how “pumping” by the convection, shear in the tachocline, and other effects contribute to all this—are still uncertain. But the relentless advance of computing power, and concomitant progress in basic theory and observation, make us think that the answers to these questions may soon be within grasp.

## Footnotes

1 For an ideal gas law $P=R\rho T/\mu _M$, $\alpha _p=\delta _t=\varphi =1$.

2 Relatively to the nuclear secular evolution of stars.

3 In an MHD system the Lorentz force comes into play and one often speaks of *magnetostrophic* balance.

4 Recall that the solar convection depth is 200 Mm or 30% in radius but only contains about 2–3% of the solar mass.

5 Mean field 2-D simulations of solar-like stars based on the $\varLambda$-effect have also been performed, see for instance Küker and Rüdiger (2007), Küker et al. (2011), Küker and Rüdiger (2012).
